# Dysregulated MicroRNAs in Urinary Non-Muscle-Invasive Bladder Cancer: From Molecular Characterization to Clinical Applicability

**DOI:** 10.3390/cancers17172768

**Published:** 2025-08-25

**Authors:** Nouha Setti Boubaker, Aymone Gurtner, Sami Boussetta, Isabella Manni, Ahmed Saadi, Haroun Ayed, Livia Ronchetti, Ahlem Blel, Marouene Chakroun, Seif Mokadem, Zeineb Naimi, Mohamed Ali Bedoui, Linda Bel Haj Kacem, Khedija Meddeb, Soumaya Rammeh, Mohamed Riadh Ben Slama, Slah Ouerhani, Giulia Piaggio

**Affiliations:** 1 UOSD SAFU Unit, Department of Research, Diagnosis and Innovative Technologies, IRCCS-Regina Elena National Cancer Institute, 00144 Rome, Italy; aymone.gurtner@ifo.it (A.G.); isabella.manni@ifo.it (I.M.); livia.ronchetti@ifo.it (L.R.); 2 Urology Department, Charles Nicolle Hospital, Faculty of Medicine, University of Tunis El Manar, Boulevard 9 Avril 1938, Tunis 1006, Tunisia; dr.saadi.ahmed@gmail.com (A.S.); haroun.ayed@fmt.utm.tn (H.A.); marouenechakroun@gmail.com (M.C.); mokadem.seif@gmail.com (S.M.); mohamedali.bedoui@etudiant-fmt.utm.tn (M.A.B.); riadhbenslama@yahoo.com (M.R.B.S.); 3 Laboratory of Proteins Engineering and Bioactive Molecules (LIP-MB), Biology Engineering Department, National Institute of Applied Sciences and Technology (INSAT), University of Tunis Carthage, Tunis 1080, Tunisia; slah.ouerhani@insat.ucar.tn; 4 Institute of Translational Pharmacology (IFT), National Research Council (CNR), 00133 Rome, Italy; 5 Laboratory of Genetics, Immunology and Human Pathology, Biology Department, Faculty of Science of Tunis, University of Tunis El Manar, Tunis 2092, Tunisia; sami-boussetta@hotmail.com; 6 Pathology Department, Charles Nicolle Hospital, Faculty of Medicine, University of Tunis El Manar, Tunis 1006, Tunisia; blelahlem@gmail.com (A.B.); linda.belhadjkacem@fmt.utm.tn (L.B.H.K.); soumaya.rammeh@fmt.utm.tn (S.R.); 7 Medical Oncology Department, Salah Azaiez Institute, Faculty of Medicine, University of Tunis El Manar, Tunis 1006, Tunisia; zeineb.naimi@fmt.utm.tn (Z.N.); khedija.meddeb@fmt.utm.tn (K.M.)

**Keywords:** bladder cancer, biomarker, microRNA, prognosis, pathway enrichment, drug interaction

## Abstract

Background: Despite clinical and pathological risk tools, predicting outcomes in non-muscle-invasive bladder cancer (NMIBC), particularly high-grade (HG) cases, remains challenging due to its unpredictable recurrence and progression. There is an urgent need for molecular biomarkers to enhance risk stratification and guide treatment. Methods: We assessed the prognostic potential of eight miRNAs (miR-9, miR-143, miR-182, miR-205, miR-27a, miR-369, let-7c, and let-7g) in a cohort of ninety patients with primary bladder cancer. Expression data were retrieved from our previously published studies. Kaplan–Meier’s and Cox’s regression analyses were used to evaluate the associations with overall survival (OS), metastasis-free survival (MFS), and clinical outcomes. Principal component analysis (PCA) was performed to identify informative miRNA combinations. Target gene prediction, pathway enrichment (DAVID), and drug–gene interaction mapping (DGIdb) were conducted in silico. Results: A high expression of let-7g and miR-9 was significantly associated with better OS in HG NMIBC and MIBC, respectively (*p* = 0.013 and *p* = 0.000). MiR-9 downregulation correlated with metastasis in MIBC (*p* = 0.018). Among all combinations, miR-205 and miR-27a best predicted intermediate-risk NMIBC progression and recurrence (r^2^ = 0.982, *p* = 0.000). A functional analysis revealed that these miRNAs regulate key cancer-related pathways (MAPK, mTOR, and p53) through genes such as TP53, PTEN, and CDKN1A. Drug interaction mapping identified nine target genes (e.g., DAPK1, ATR, and MTR) associated with eight FDA-approved bladder cancer therapies, including cisplatin and gemcitabine. Conclusions: Let-7g, miR-9, miR-143, miR-182, and miR-205 emerged as promising biomarkers for outcome prediction in NMIBC. Their integration into liquid biopsy platforms could support non-invasive monitoring and personalized treatment strategies. These findings warrant validation in larger, prospective studies and through functional assays.

## Simple Summary

This research addresses the critical need for more reliable prognostic biomarkers in non-muscle-invasive bladder cancer (NMIBC), especially high-grade tumors, where current clinical tools fall short in predicting outcomes. This study investigates the prognostic potential of a panel of microRNAs (miR-9, miR-182, miR-205, miR-27a, miR-369, let-7c, and let-7g) through the integrative analyses of patient survival, molecular targets, and drug interactions. By combining bioinformatics tools with clinical data, we aimed to identify miRNAs that not only correlate with disease progression and metastasis but also reveal mechanistic insights relevant to treatment. The findings may facilitate the development of non-invasive biomarkers, enhance risk stratification, and inform targeted therapeutic strategies. This study lays the groundwork for future validation and functional research, with the potential to significantly impact personalized care in bladder cancer.

## 1. Introduction

Urinary bladder cancer (BCa) is among the most frequently diagnosed malignancies worldwide and ranks first among urological cancers, accounting for approximately 3% of all new cancer cases in both sexes [1,2]. At diagnosis, nearly 70% of patients present with non-muscle-invasive bladder cancer (NMIBC), limited to the mucosa (CIS or Ta) or lamina propria (T1). While NMIBC generally carries a favorable prognosis, with a low risk of progression to muscle-invasive disease (MIBC), it is characterized by a high recurrence rate (50–70%), necessitating long-term surveillance and repeated interventions, making it one of the costliest cancers to manage [3,4]. These tumors exhibit significant clinical, cytogenetic, and histological heterogeneity, and may present variable risks of progression even within the same stage [5]. This variability reflects the existence of two distinct carcinogenic pathways: one driven by hyperplasia, which gives rise to low-grade (LG) NMIBC and is marked by the activation of oncogenes such as FGFR3 and RAS; and another associated with dysplasia and/or carcinoma in situ (CIS), leading to MIBC through alterations in tumor suppressor genes like TP53, RB1, and PTEN. High-grade NMIBC is thought to result from the convergence of both pathways, suggesting a molecular overlap between the non-invasive and invasive forms [5,6,7,8]. Although most non-muscle-invasive bladder cancers (NMIBC) have a favorable prognosis, with a low progression rate (10–20%) and a 5-year overall survival of approximately 90%, they often recur shortly after initial resection (in 50–70% of cases). This high recurrence necessitates frequent surveillance and repeated interventions, making NMIBC one of the costliest cancers to manage. While clinical and pathological features integrated into EORTC risk tables offer some prognostic value, they remain inadequate for reliably predicting outcomes, particularly in high-grade NMIBC [9,10]. These limitations highlight the urgent need for novel molecular biomarkers to enhance risk stratification and inform treatment decisions [9,11,12,13]. MicroRNAs (miRNAs), a class of small non-coding RNAs that regulate gene expression post-transcriptionally, have emerged as promising candidates in this regard. Aberrant miRNA expression is implicated in tumor initiation, progression, and metastasis across various cancers, including BCa. Certain miRNAs also demonstrate tissue-specific activity and hold potential as diagnostic, prognostic, and predictive biomarkers. Importantly, recent studies highlight the role of miRNAs in modulating treatment response by targeting genes involved in drug efficacy, offering new perspectives for personalized therapy. Collectively, all these facts have urged the translation of miRNAs into clinical medicine [14,15,16,17,18,19,20,21,22]. Building on our previous work, we investigated the prognostic potential of a panel of eight miRNAs, miR-9, miR-182, miR-143, miR-27a, miR-205, miR-369, let-7c, and let-7g, selected based on existing evidence linking them to key oncogenic processes in BCa [23,24,25,26,27,28,29]. This study aimed to evaluate their association with overall survival, metastasis, progression, and recurrence in advanced BCa, particularly high-grade NMIBC. Additionally, in silico analyses were conducted to explore their mRNA targets, regulatory networks, implicated signaling pathways, and drug–gene interactions.

## 2. Materials and Methods

### 2.1. Dataset and miRNA Expression Patterns

Clinical data and formalin-fixed paraffin-embedded (FFPE) tissue expression profiles of miR-9, miR-143, let-7c, let-7g, miR-27a, miR-182, miR-205, and miR-369 were retrieved from the database established in our previously published studies [30,31,32,33]. Ninety BCa primary tumors and ten control samples obtained from the non-tumoral zone of cystectomy specimens used as controls were enrolled. These zones were identified on H&E-stained reference slides by two pathologists as histologically normal areas, spatially separated from the tumor, typically by at least 1 to 2 cm or clearly demarcated by normal histological structures. Corresponding unstained sections were microdissected under RNase-free conditions using the punch method, which enables the selective retrieval of tumor or non-tumor tissue cores directly from defined histological regions within the FFPE block while minimizing contamination from adjacent tissue. Areas containing tumor cells, necrosis, or dense inflammatory infiltrates were excluded.

Samples were collected from the Department of Pathology between 2015 and 2017 from patients who underwent a transurethral resection of the bladder tumor (TURBT) or radical cystectomy at the Urology Department of Charles Nicolle Hospital (Tunis, Tunisia). Patients were excluded if they had a prior TURBT, pT1 tumors that had progressed to muscle-invasive BCa (MIBC), metastatic disease, prior chemotherapy or radiotherapy, or other concomitant malignancies. Importantly, all MIBC patients were chemotherapy-naïve at the time of analysis.

The detailed clinical and pathological characteristics of the cohort are provided in Table 1.

Patients with NMIBC were stratified into risk groups based on the European Organization for Research and Treatment of Cancer (EORTC) risk tables, which estimate recurrence and progression probabilities (Table 2). Recurrence and progression outcomes were then compared to the EORTC predictive model (http://www.eortc.be/tools/bladdercalculator accessed on 31 July 2025).

All procedures were conducted in accordance with ethical standards and were approved by the Ethics Committee of Charles Nicolle Hospital, Tunis (approval date: 17 March 2016), in compliance with the 1964 Helsinki Declaration and its subsequent amendments. Informed consent was obtained from all participants.

**Figure 1:**
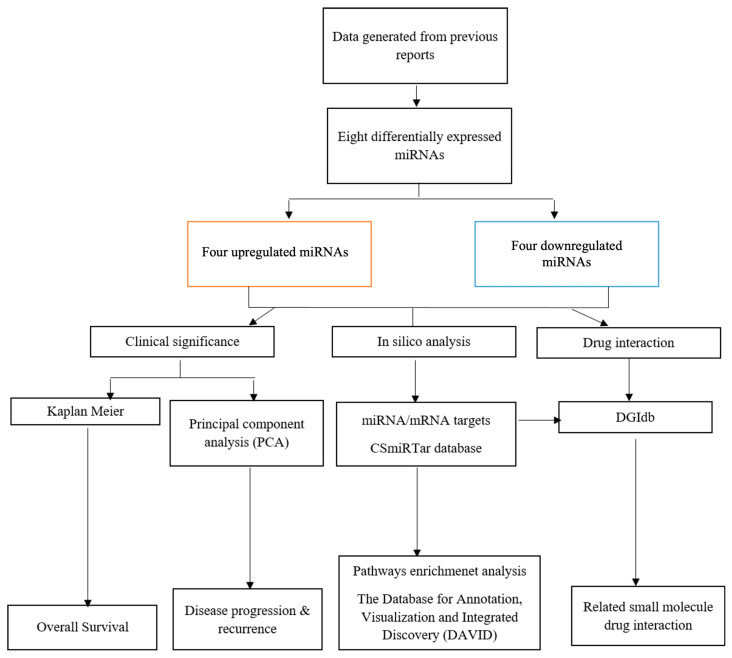
Study design flowchart.

The study design and workflow are illustrated in Figure 1.

### 2.2. Statistical Approaches for Survival and Metastasis Analysis

For each selected miRNA, an optimal fold change (FC) threshold was determined using Receiver Operating Characteristic (ROC) curve analysis in software (version 25.0, SPSS Inc., Chicago, IL, USA), and the optimal cut-off points were identified using the Youden index (sensitivity + specificity − 1) (Table 3). These cut-off values were established to distinguish between low- and high-expression groups.

Subsequently, Kaplan–Meier’s survival analysis with the log-rank test was performed to evaluate the association between miRNA expression levels and overall survival (OS), progression-free survival (PFS), and metastasis-free survival (MFS) in patients with high-grade NMIBC and MIBC. miRNA expression was treated as an independent variable, and survival curves were compared using the log-rank test. The median follow-up period was 36 months, and only patients with at least one complete follow-up record indicating vital status were included.

OS was defined as the time from surgery to death or last follow-up. PFS referred to the interval between surgical treatment and disease progression, while MFS was defined as the time from treatment to the first detection of distant metastasis or death due to metastasis.

All statistical analyses were conducted using SPSS software (version 25.0, SPSS Inc., Chicago, IL, USA), and *p*-values < 0.05 were considered statistically significant.

### 2.3. Statistical Approach for the Multivariate Analysis in Patients with NMIBC: Principal Component Analysis (PCA)

Given the heterogeneity of the dataset, principal component analysis (PCA) was employed to perform multivariate analysis. PCA reduces the dimensionality of complex datasets by generating linear combinations of variables, called principal components, that capture the greatest variance within the data [34]. This method transforms a large set of potentially interrelated variables into a smaller, more interpretable set [35]. We applied PCA to identify meaningful expression patterns among miRNAs in NMIBC patients, particularly in relation to histological grade (LG vs. HG) and EORTC-defined prognostic groups for progression and recurrence (mainly intermediate and high risk). These groups are defined by a scoring system based on six clinicopathological variables namely number of tumors, tumor size, prior recurrence rate, tumor stage, tumor grade, and the presence of carcinoma in situ (CIS). The analyses were performed using correlation matrices as the basis for component extraction.

### 2.4. miRNAs Target Gene Prediction and Functional Enrichment Analysis

The predicted and/or validated target genes of miR-9, miR-143, let-7g, let-7c, miR-27a, miR-182, miR-205, and miR-369 in urothelial BCa were retrieved from the CSmiRTar database (http://cosbi4.ee.ncku.edu.tw/CSmiRTar/), using disease-specific filters. “Urinary Bladder Neoplasms” was selected as the disease filter, and targets supported by at least two databases were retained. The targets were ranked by Average Normalized Score (ANS), which reflects the mean confidence score across databases, with a higher ANS indicating greater prediction reliability [36]. The top 30 genes with the highest ANS were used to construct a miRNA–mRNA regulatory network via Gephi 0.9.2 (https://gephi.org).

To explore the biological relevance of these target genes, functional enrichment analysis was performed using DAVID (https://davidbioinformatics.nih.gov), incorporating KEGG and BIOCARTA pathway annotations. Only pathways with a *p*-value < 0.05 were considered statistically significant.

### 2.5. miRNAs-mRNAs’ Target Gene Drug Interaction Analysis

The interactions between the mRNA targets of all investigated miRNAs and corresponding therapeutic agents were assessed using the Drug–Gene Interaction Database (DGIdb, https://www.dgidb.org/). DGIdb integrates data from multiple resources, including the DrugBank, ChEMBL, NCBI, Ensembl, PharmGKB, PubChem, clinical trials, and PubMed literature, to identify and curate known drug–gene interactions. It prioritizes these interactions using comparative scores (interaction and query scores), which were applied for ranking rather than fixed thresholds, to explore whether miRNA-regulated genes may represent viable therapeutic targets or be prioritized as candidates for drug development [37]. All miRNA–mRNA targets identified in Section 2.4 (Supplementary Table S2) were included in the analysis. Only interactions involving FDA-approved drugs for bladder cancer (https://www.cancer.gov/about-cancer/treatment/drugs/bladder) (accessed on 1 September 2023) were considered. The resulting drug–gene interaction network was visualized using Cytoscape (version 3.10.0).

## 3. Results

### 3.1. Association of the Deregulated miRNAs’s Expression to Overall Survival

**Figure 2:**
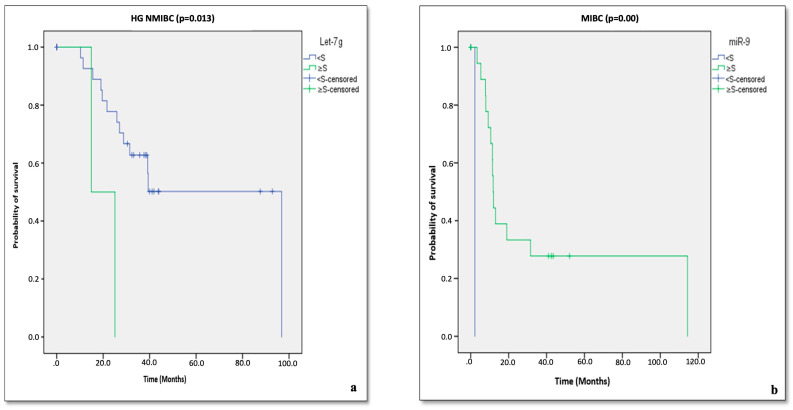
Kaplan–Meier’s survival analysis of overall survival of patients with HG NMIBC and MIBC according to Let-7g (**a**) and miR-9’s (**b**) relative expression. S—threshold value, HG NMIBC—High-Grade Non-Muscle Invasive Bladder Cancer; MIBC—Muscle Invasive Bladder Cancer.

Among the analyzed miRNAs, only let-7g and miR-9 showed significant prognostic value for overall survival (OS) in patients with HG NMIBC and MIBC, respectively (*p* = 0.013 and *p* = 0.000; Figure 2a,b). Specifically, HG NMIBC patients with let-7g expression below the threshold value (07.67) demonstrated improved OS (Figure 2a). Similarly, MIBC patients with miR-9 expression at or above the threshold (02.05) had a better survival probability (Figure 2b).

**Figure 3:**
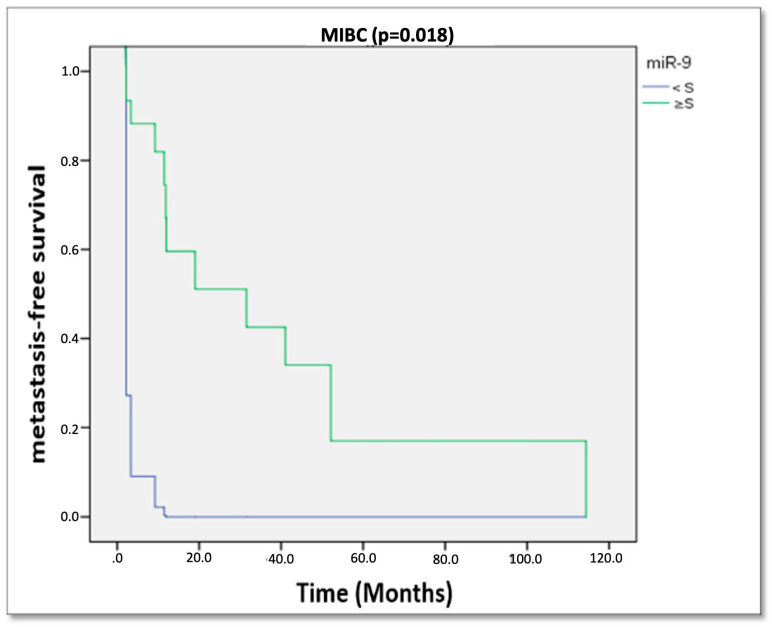
Cox’s regression analysis of the metastasis-free survival based on the expression levels of miR-9 in patients with MIBC. S = threshold value, MIBC = Muscle Invasive Bladder Cancer.

Cox’s regression analysis further indicated that none of the studied miRNAs were significantly associated with progression in HG NMIBC. However, miR-9 expression was significantly associated with metastasis in MIBC patients (*p* = 0.018; Figure 3). Notably, patients with miR-9 expression equal to or above the threshold exhibited improved metastasis-free survival.

### 3.2. PCA of the Relative Expression of the Studied miRNAs According to the Histological Grade and EORTC Scores of Progression and Recurrence

Using principal component analysis (PCA), we established a reduced multivariate model consisting of miR-143, miR-9, miR-182, and miR-205, which accounted for 73.75% of the total variance in NMIBC (Supplementary Table S1A). When stratified by tumor grade, the PCA revealed that in LG NMIBC, miR-9 and miR-182 alone explained 51.14% of the total variability (Supplementary Table S1B). In contrast, the combination of miR-143, miR-9, miR-182, and miR-205 explained 66.04% of the variance in HG NMIBC (Supplementary Table S1C).

**Figure 4:**
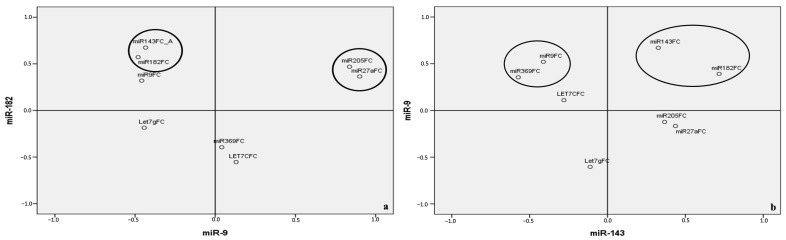
Projection of miRNAs’s expression patterns onto predefined axes according to PCA in LG NMIBC (**a**) and HG NMIBC (**b**). LG NMIBC—combination 1: [miR-205, miR-27a] (r2 = 0.958, *p* = 0.00); combination 2: [miR-143, miR-182] (r^2^ = 0,543, *p* = 0,02). HG NMIBC—combination 1: [miR-143, miR-182] (r^2^ = 0.343, *p* = 0.01); combination 2: [miR-9, miR-369] (r^2^ = 0.281, *p* = 0.03). PCA—principal component analysis; LG NMIBC—Low-Grade Non-Muscle-Invasive Bladder Cancer; HG NMIBC—High-Grade Non-Muscle-Invasive Bladder Cancer; FC—Fold change.

The projection of data onto the axes capturing the highest variance further highlighted distinct miRNA groupings. In LG NMIBC, two clusters were observed: [miR-205, miR-27a] (r^2^ = 0.958, *p* = 0.000) and [miR-143, miR-182] (r^2^ = 0.543, *p* = 0.02) (Figure 4a). Similarly, HG NMIBC was characterized by [miR-143, miR-182] (r^2^ = 0.343, *p* = 0.01) and to a lesser extent, [miR-9, miR-369] (r^2^ = 0.281, *p* = 0.03) (Figure 4b).

Further PCA based on EORTC risk classifications showed that the same four-miRNA model (miR-9, miR-143, miR-182, and miR-205) explained 64.59% and 81.1% of the variance associated with high and intermediate progression risk, respectively (Supplementary Table S1.D.1 and D.2). Additionally, this model accounted for 73.76% of the variance in intermediate recurrence risk and up to 91.58% of the total variance related to recurrence in NMIBC overall (Supplementary Table S1.E.1 and E.2).

**Figure 5:**
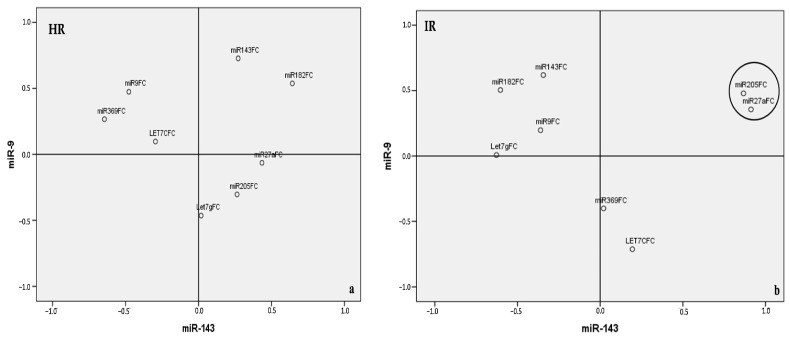
Projection of miRNAs’s expression patterns onto predefined axes according to PCA related to EORTC risk scores of progression in NMIBC. (**a**) EORTC high risk of progression group—combination 1: [miR-9, miR-369] (r^2^ = 0.284, *p* = 0.029); combination 2: [miR-143, miR-182] (r2 = 0.341, *p* = 0.01). (**b**) EORTC intermediate risk of progression group—[miR-205, miR-27a] (r^2^ = 0.982, *p* = 0.000). PCA—principal component analysis, NMIBC—Non-Muscle-Invasive Bladder Cancer; FC—Fold change; EORTC—European Organization of Research and Treatment of Cancer; HR—High Risk; IR—Intermediate risk.

**Figure 6:**
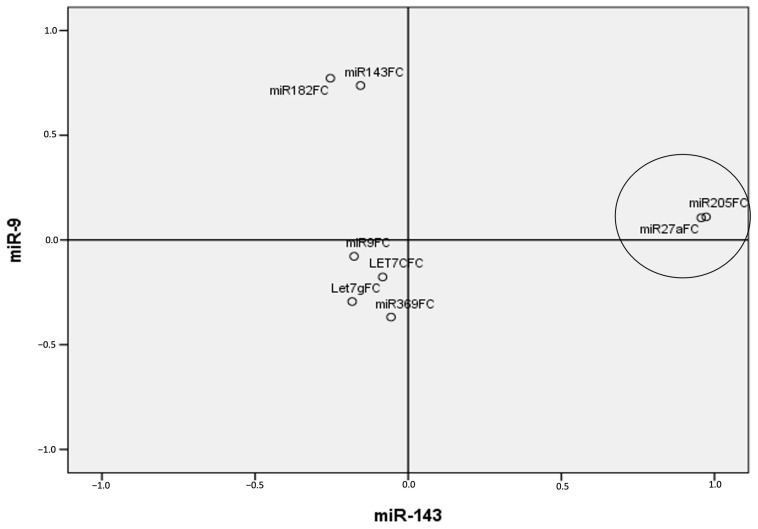
Projection of miRNA’s expression patterns onto predefined axes according to PCA related to EORTC intermediate risk scores of recurrence in NMBC. Combination 1: [miR-9, miR-369] (r^2^ = 0.250, *p* = 0.045); combination 2: [miR-143, miR-182] (r^2^ = 0.349, *p* = 0.008); combination 3: [miR-205, miR-27a] (r^2^ = 0.933, *p* = 0.000). PCA—principal component analysis, NMIBC—Non-Muscle-Invasive Bladder Cancer; FC—Fold change; EORTC—European Organization of Research and Treatment of Cancer; HR—High Risk; IR—Intermediate risk.

Finally, when projecting EORTC-defined progression and recurrence risks onto the principal axes (defined by miR-9 and miR-143), three miRNA combinations emerged as key contributors: [miR-9, miR-369], [miR-143, miR-182], and [miR-205, miR-27a] (Figure 5a,b and Figure 6).

### 3.3. Target Gene Prediction and Functional “In Silico” Enrichment Analysis

Target genes of the eight studied miRNAs were identified using CSmiRTar’s functional filters, yielding both predicted and experimentally validated targets (Supplementary Table S2A–H). Notably, let-7c, let-7g, miR-9, and miR-182 were found to regulate several tumor suppressor genes, including Cyclin-Dependent Kinase Inhibitor 1A (*CDKN1A*), Mitogen-Activated Protein Kinase Kinase 7 (*MAP2K7*), Patched 1 (*PTCH1*), Tumor Protein p53 (*TP53*), Phosphatase and Tensin Homolog (*PTEN*), Hypoxia-Inducible Factor 1 Subunit Alpha (*HIF1A*), GATA Binding Protein 3 *(GATA3*), and Glutathione S-Transferase Mu 1 (*GSTM1*), previously implicated in BCa progression, recurrence, and poor prognosis [38,39,40,41,42,43,44] (Supplementary Table S2A–D). Conversely, miR-27a, miR-205, miR-143, and miR-369 targeted oncogenes such as Death-Associated Protein Kinase *1* (*DAPK1*), Caspase 3 (*CASP3*), DNA Methyltransferase 1 (*DNMT1*), Erb-B2 Receptor Tyrosine Kinase 2 (*ERBB2*), BCL2 Apoptosis Regulator (*BCL2*), Phosphatidylinositol-4,5-Bisphosphate 3-Kinase Catalytic Subunit Alpha (*PIK3CA*), Protein Kinase Inhibitor Alpha (*PKIA*), and ABL Proto-Oncogene 2, Non-Receptor Tyrosine Kinase (*ABL2*), which are known to be dysregulated in bladder cancer and associated with aggressive tumor behavior [41,45,46,47,48,49,50] (Supplementary Table S2E–H).

**Figure 7:**
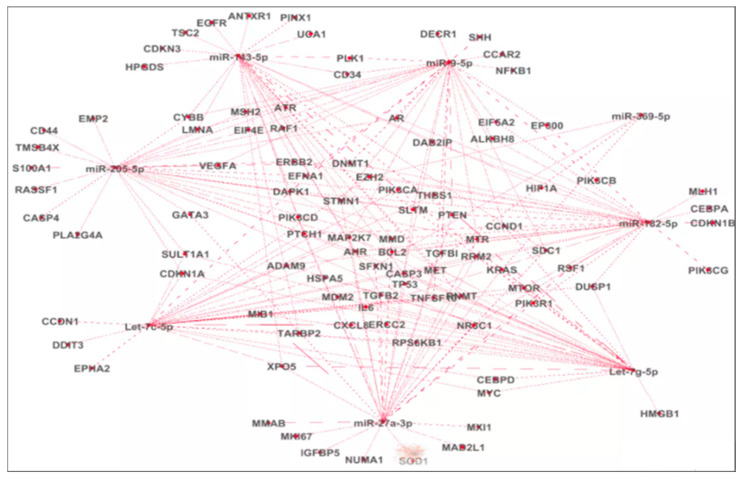
Integrated miRNAs–mRNA interaction network in urothelial Bca. miRNA—microRNA; mRNA—messenger RNA; BCa—bladder cancer; round central nodes—miRNA; peripheral nodes—target mRNA. The “line” between the central node and each peripheral node indicates an interaction between the pinpointed miRNA and its target RNA. Target genes were retrieved from CSmiRTar databases, MiRNA–mRNA interactions were visualized using Gephi v.0.9.2. We represented in this network the top thirty target genes with the highest ANS.

The miRNA–mRNA interaction network also revealed several shared targets across subsets of the candidate miRNAs, including *RRM2* (common target of miR-27a-3p, Let-7g-5p, and miR-9-5p), *XPO5* (common target of miR-27a-3p, Let-7c-5p, and miR-143), *PTEN* (common target of miR-27a-3p, miR-9-5p, and Let-7g-5p), *CCND1* (common target of miR-27a-3p, Let-7c-5p, miR-205-5p, and Let-7g-5p), *MAP2K7*, *STMN1*, *TP53* (common targets of miR-27a-3p, and miR-9-5p), *DNMT1* (common target of miR-27a-3p, miR-9-5p, and miR-143-5p), and *LMNA* (common target of miR-9, miR-182, miR-14,3 and miR-205), suggesting partial overlap in their regulatory interactions and convergence on common signaling pathways (Figure 7).

To further elucidate the biological impact of these targets, we conducted pathway enrichment analysis using the DAVID tool, applying BIOCARTA and KEGG databases and restricting the results to Homo sapiens (*p* < 0.05). The enriched pathways (Supplementary Table S3A–H) included MAPK, mTOR, FOXO, TNF, AKT, p53, apoptosis, RAP1, and ErbB signaling, which are well established in bladder cancer pathogenesis, progression, and treatment resistance [48,51,52,53,54,55].

**Figure 8:**
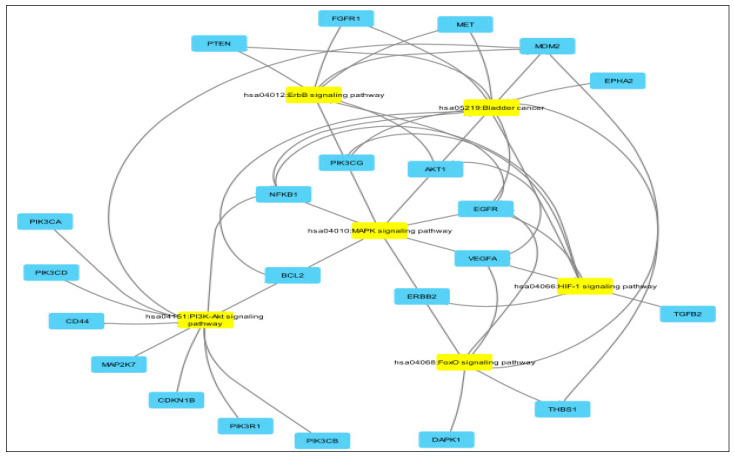
Analysis of common signaling pathways to miR-9, miR-182, miR-143, Let-7c, Let-7g, miR-205, miR-27a, and miR-369.

A pathway overlap analysis also revealed commonly enriched circuits such as PI3K–Akt, HIF-1, p53, MAPK, chemokine, and cell cycle pathways (Figure 8).

Additionally, individual miRNAs were associated with unique pathways, including let-7c/ER protein processing, miR-9/serotonergic synapse, miR-182/TGF-β signaling, and miR-143/hypertrophic cardiomyopathy and rheumatoid arthritis, further supporting their diverse functional roles.

### 3.4. miRNA–mRNA–Drug Interaction Prediction

The mRNA targets of the selected miRNAs were further explored as potential therapeutic candidates using the DGIdb database, focusing exclusively on FDA-approved drugs for bladder cancer. This screening identified eight drugs: Gemcitabine, Cisplatin, Nivolumab, Pembrolizumab, BCG vaccine, Doxorubicin, Erdafitinib, and Pemigatinib (Table 4).

**Figure 9:**
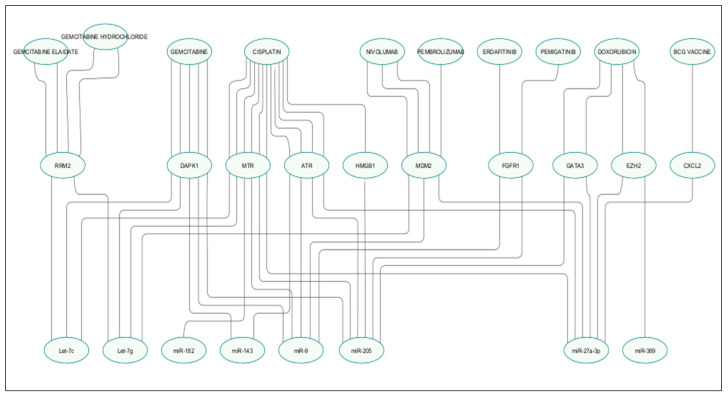
Drug–mRNA–miRNA gene interaction network. Nodes represented drugs, mRNA genes, or miRNAs. The lines represent interaction relationship between drug–mRNA gene and mRNA target–miRNA.

These agents were found to interact with nine mRNA targets—RRM2, DAPK1, MTR, MDM2, FGFR1, ATR, CXCL2, GATA3, and EZH2—which were shared or uniquely regulated by let-7c, let-7g, miR-9, miR-27a-3p, miR-143, miR-182, miR-205, and miR-369 (Table 4, Figure 9).

Notably, FGFR1, a common target of miR-9 and miR-205, was linked to Erdafitinib and Pemigatinib, both of which have been evaluated in clinical trials, underscoring the therapeutic relevance of these miRNA–mRNA–drug interactions.

## 4. Discussion

Urinary bladder cancer (BCa) remains a prevalent malignancy with significant heterogeneity in clinical outcomes and highly expensive management procedures. Approximately 75% of newly diagnosed bladder cancer cases are classified as non-muscle-invasive (NMIBC). Among these, high-grade (HG) tumors pose significant management challenges due to their unpredictable clinical course. Despite diagnostic advancements, the absence of reliable biomarkers continues to hinder effective risk stratification and personalized treatment, particularly in intermediate- and high-risk NMIBC [56,57].

In this context, miRNAs have been described as promising molecular candidates for diagnostic, prognostic, and therapeutic applications across various cancers, including urothelial carcinoma [58,59]. Nevertheless, most studies have focused on MIBC. As a result, data on miRNAs specifically relevant to NMIBC remain limited, hindering their clinical translation in this subgroup.

Given this background, we evaluated a panel of miRNAs, namely miR-9, miR-182, miR-143, miR-205, miR-27a, let-7c, let-7g, and miR-369, for their potential roles in the prognostic stratification of BCa, with a particular focus on HG NMIBC. Our findings indicate that the low expression of let-7g was significantly associated with improved overall survival (OS) in HG NMIBC (Figure 3), highlighting its potential utility as a prognostic biomarker in early-stage disease. To our knowledge, this is one of the first reports implicating let-7g in BCa prognosis, although its tumor-suppressive functions have been previously described in other malignancies [19,60,61].

Interestingly, miR-9 exhibited divergent behavior between NMIBC and MIBC. While its upregulation in HG NMIBC was linked to poor prognosis and disease progression, elevated levels in MIBC were associated with improved OS and reduced metastatic spread. This dual role underscores the importance of tumor context and suggests subtype-specific regulatory mechanisms that merit further investigation.

These findings highlight the potential of this biomarker for early diagnosis, monitoring, and prognostic assessment in BCa, particularly in HG NMIBC, which remains clinically challenging. Validating its use in liquid biopsies could reduce the reliance on invasive tissue sampling and support more personalized therapeutic strategies.

Although our results contrast with some BCa studies [23] they align with observations in acute myeloid leukemia [62]. Such discrepancies may stem from intratumoral heterogeneity, reflecting distinct histological subtypes and complex regulatory mechanisms at both the transcriptional and post-transcriptional levels (46) [63,64].

To explore the biological relevance of these miRNAs, we performed in silico target prediction using the CsmiRtar tool (Supplementary Table S2) (Figure 7). Oncogenic miRNAs (e.g., miR-9, miR-182, let-7c, and let-7g) were found to regulate a wide array of mRNA targets implicated in tumorigenesis (Figure 7), including *NFKB1*, *CCDN1*, *DNMT1*, *PIK3C* (A,B), *RAF1*, *PTEN*, *BCL2*, *MET*, *ERBB2*, and *TP53*, implicated in the initiation of urothelial tumorigenesis, patient prognosis, tumor progression, cell proliferation, and survival [48,54,65,66,67,68,69,70,71,72]. Conversely, tumor suppressor miRNAs (e.g., miR-143, miR-27a, miR-205, and miR-369) targeted key genes, such as *PLK1*, *PIK3CA*, *EGFR*, *AKT1*, *BCL2*, *HMGB1*, *MDM2*, *BCL2*, *RAF1*, and *CDKN1* (Figure 7), which have been described in the literature to initiate pathogenesis, enhance the proliferation, invasion, and migration of cancerous cells, tumor recurrence and progression, patient survival, and the prediction of the response to treatment [73,74,75].

The deregulation of these target genes is known to affect the pathways central to BCa pathophysiology, including PI3K/Akt/mTOR, RAS/MAPK, p53, FOXO, TNF, and IL-2 signaling (Supplementary Table S3) [51,52,54,55,76]. Notably, several targets were shared across multiple miRNAs, suggesting the possibility of coordinated regulatory networks with implications for tumor behavior and treatment response (Figure 8).

By integrating miRNA expression profiles with clinical parameters (e.g., grade, progression, and recurrence), we developed a reduced prognostic model for NMIBC. A four-miRNA panel (miR-9, miR-143, miR-182, and miR-205) captured 66% of the prognostic variability in HG NMIBC. In low-grade (LG) NMIBC, a simplified model based on miR-9 and miR-182 explained over 50% of the variance. These findings emphasize the biological complexity of HG NMIBC and suggest that miRNA-based models could enhance patient stratification beyond current histopathological criteria.

A particularly notable result was the identification of a miR-205/miR-27a combination associated with intermediate progression and recurrence risk in NMIBC (Figure 5b and Figure 6), a subgroup often characterized by clinical unpredictability. This pair may offer a promising marker set for the improved prediction of recurrence and progression, potentially guiding treatment intensification or closer surveillance.

**Figure 10:**
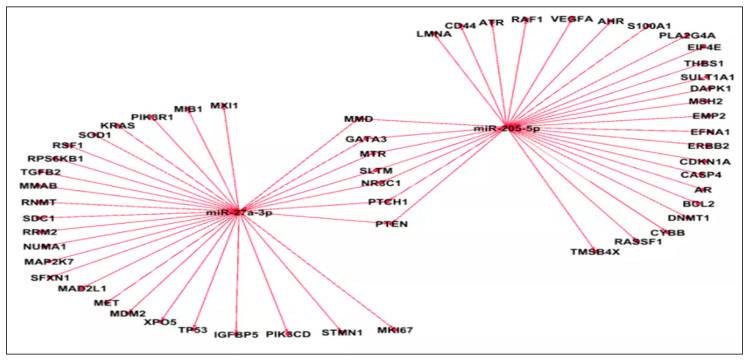
Integrated analysis of miR-205/miR-27a-mRNAs’s common and distinct targets in urothelial BCa. miRNA—microRNA; mRNA—messenger RNA; BCa—Bladder cancer; round central nodes—miRNA; peripheral nodes—target mRNA. The “line” between the central node and each peripheral node indicates an interaction between the pinpointed miRNA and its target mRNA. Target genes were retrieved from CSmiRTar databases, MiRNA–mRNA interactions were visualized using Gephi v.0.9.2. We represented in this network the top thirty target genes with the highest ANS.

Our bioinformatic analysis also suggested that these two miRNAs share common targets, such as *GATA3*, *NR3C1*, *PTCH1*, and *PTEN* (Figure 10), previously implicated in NMIBC progression and/or recurrence [77,78,79,80,81,82], raising the possibility of a synergistic regulatory mechanism, in which cooperative miRNA–miRNA interactions modulate shared oncogenic pathways [83,84,85]. Notably, three of these common targeted genes, namely *PTEN*, *GATA3*, and *PTCH1*, have already been validated as direct in vivo targets of miR-205-5p and/or miR-27a-3p, with *PTEN* confirmed as a target of both miRNAs [86,87,88,89], and GATA3 and PTCH1 experimentally validated as miR-205 targets [90,91]. Together, these findings strongly support the reliability of our in silico predictions.

Beyond prognosis, we examined the therapeutic implications of miRNA–target–drug interactions. Key therapeutic agents for advanced BCa, including cisplatin, gemcitabine, and nivolumab, interact with genes targeted by our miRNA panel (Figure 9).

ATR, a key DNA damage response kinase, is of particular interest due to its role in mediating resistance to cisplatin. It is potentially regulated by miR-143, miR-205, and miR-27a, as identified in our study. In response to cisplatin-induced DNA synthesis inhibition and G2/M cell cycle arrest, cancer cells often upregulate ATR as a survival mechanism [92]. Therefore, restoring the expression of downregulated miRNAs that target ATR, such as miR-27a-3p, miR-143, and miR-205, may enhance cisplatin efficacy and represent a promising strategy to overcome chemoresistance in advanced bladder cancer.

Additionally, our analysis highlights the interactions between key therapeutic targets, MTR, DAPK1, and MDM2, and widely used agents in advanced BCa, including cisplatin, gemcitabine, and nivolumab [93,94]. Sensitivity to gemcitabine and cisplatin appears closely linked with the Let-7 family, miR-9, miR-27a-3p, miR-182, miR-205, and miR-143, suggesting that the miRNA regulation of targets such as RRM2, DAPK1, MTR, ATR, and HMGB1 may influence chemosensitivity and serve as predictive biomarkers. Associations between Let-7g, miR-9, and miR-27a-3p with MDM2 indicate a potential role of miRNAs in modulating p53 pathway activity and the responsiveness to immune checkpoint inhibitors. Beyond chemotherapy, miRNAs targeting GATA3, EZH2, and CXCL2, namely miR-27a-3p, miR-369, and miR-205, may affect responses to doxorubicin and BCG immunotherapy, while the regulation of FGFR1 by miR-9 and miR-205 suggests a role in selecting patients for FGFR-targeted therapies. Collectively, these findings support the potential of miRNA modulation to enhance current treatments or overcome therapeutic resistance.

Finally, we acknowledge that our study presents some limitations. The small sample size may limit the robustness and generalizability of the findings. Additionally, the retrospective design may introduce bias and prevent definitive conclusions about causality. Finally, the absence of functional assays restricts the mechanistic interpretation of the miRNA–mRNA interactions proposed. Future prospective studies with larger cohorts and experimental validation are needed to confirm these results.

## 5. Conclusions

Despite the above-mentioned limitations of the current study, these preliminary results represent a valuable research track in the field of setting diagnostic and/or prognostic biomarkers for urinary BCa to improve patients’s care scheduling. The upcoming step will be to use miR-9, miR-182, miR-143, and miR-205 as biomarkers within prospective studies conducted on liquid biopsies in order to develop non-invasive diagnostic strategies, test their applicability as an aid to clinical decision-making, and uncover their mechanisms of action through functional studies.

## Figures and Tables

**Table 1:** Clinicopathological characteristics of the studied population.

Characteristics	Value (%)
Sample size	90
Age at diagnosis	70.04 ±12.15
Gender	
Male	83/90 (92.22%)
Female	7/90 (7.78%)
Tumor groups	
NMIBC	67/90 (74.44%)
MIBC	23/90 (25.55%)
Tumor histological grade	
LG NMIBC HG NMIBC	23/67 (34.32%) 44/67 (65.67%)
Tumor T stage	
Ta	22/90 (24.45%)
T1	45/90 (50%)
T2	20/90 (22.22%)
T3	3/90 (3.33%)

LG NMIBC—Low-Grade Non-Muscle-Invasive Bladder Cancer; HG NMIBC—High-Grade Non-Muscle-Invasive Bladder Cancer; MIBC—Muscle-Invasive Bladder Cancer.

**Table 2:** EORTC stratification for progression and recurrence in NMIBC.

Clinical Parameters	Score	Probability of Recurrence or Progression at 1 Year (%)	Probability of Recurrence or Progression at 5 Years (%)	Recurrence or Progression Risk Groups	Average
Recurrence	5–9	38 (35–41)	62 (58–65)	Intermediate risk	56/63 (88.89%)
	10–17	61 (55–67)	78 (73–84)	High risk	7/63 (11.11%)
Progression	2–6	1 (0,4–1,6)	6 (5–8)	Intermediate risk	14/63 (22.23%)
	7–13	5 (4–7)	17 (14–20)	High risk	49/63 (77.77%)

EORTC—European Organization of Research and Treatment of Cancer; NMIBC—Non-Muscle-Invasive Bladder Cancer.

**Table 3:** Candidate MiRNAs threshold values relative to their relative expression (FC).

miRNA	Threshold Values (S)	G1 (<S) (%)	G2 (≥S) (%)
miR-9	2.05	37.32	62.68
miR-182	47.23	70.15	29.85
Let-7g	7.67	66.66	33.34
miR-143	0.01	58.20	41.80
miR-205	1.58 × 10^−5^	1.49	98.51
miR-369	1.23 × 10^−5^	57.78	42.22
miR-27a	5.85 × 10^−5^	12.22	87.78
Let-7c	5.67	72.22	27.78

FC = fold change; G1—group 1; G2—group 2. Threshold values for all candidate miRNAs were determined using time-dependent ROC curve analysis in software (version 25.0, SPSS Inc., Chicago, IL, USA), and the optimal cut-off points were identified using the Youden index (sensitivity + specificity − 1), which makes it possible to distinguish between two groups of patients. The first group has an expression level lower than the threshold value of “FC” and conversely the second group is characterized by an expression greater than or equal to the fixed threshold of FC.

**Table 4:** Candidate drugs targeting the miRNAs-mRNA target.

miRNA	mRNA Target	Drug	Interaction Score *	Interaction Type	Studies PMIDs
Let-7c	RRM2	GEMCITABINE ELAIDATE	5.15	-	-
		GEMCITABINE HYDORCHLORIDE	0.74	Inhibitor	-
	DAPK1	GEMCITABINE	1.53	-	22293537
	MTR	CISPLATIN	0.57		21605004 29662106 19159907
Let-7g	RRM2	GEMCITABINE ELAIDATE	5.15	-	-
		GEMCITABINE HYDORCHLORIDE	0.74	Inhibitor	-
	DAPK1	GEMCITABINE	1.53	-	22293537
	MTR	CISPLATIN	0.57		21605004 29662106 19159907
	MDM2	NIVOLUMAB	0.52	-	28351930
miR-9	MTR	CISPLATIN	0.57	-	21605004 29662106 19159907
	FGFR1	ERDAFITINIB	0.73	Inhibitor	26324363 28341788 28965185
		PEMIGATINIB	0.48	Inhibitor	32315352
	DAPK1	GEMCITABINE	1.53	-	22293537
	ATR	CISPLATIN	0.11	-	12894503
	MDM2	NIVOLUMAB	0.52	-	28351930
miR-27a-3p	MTR	CISPLATIN	0.57	-	21605004 29662106 19159907
	MDM2	NIVOLUMAB	0.52	-	28351930
		PEMBROLIZUMAB	0.35	-	28351930
	CXCL2	BCG VACCINE	2.06	-	18217952
	GATA3	DOXORUBICIN	0.18		24141364
	EZH2	DOXORUBICIN	0.19	-	25605023
	ATR	CISPLATIN	0.11	-	12894503
miR-143	DAPK1	GEMCITABINE	1.53	-	22293537
	ATR	CISPLATIN	0.11	-	12894503
miR-182	MTR	CISPLATIN	0.57	-	21605004 29662106 19159907
miR-205	MTR	CISPLATIN	0.57	-	21605004 29662106 19159907
	HMGB1	CISPLATIN	0.12	-	9427537 8968078
	GATA3	DOXORUBICIN	0.18	-	24141364
	FGFR1	ERDAFITINIB	0.73	Inhibitor	26324363 28341788 28965185
		PEMIGATINIB	0.48	Inhibitor	32315352
	DAPK1	GEMCITABINE	1.53	-	22293537
	ATR	CISPLATIN	0.11	-	12894503
miR-369	EZH2	DOXORUBICIN	0.19	-	25605023

* The interaction score is the combined number of database sources and PubMed references supporting a given interaction according to DGIdb database.

## Data Availability

The raw data of the study are available from the corresponding author upon reasonable request.

## Supplementary Materials

The following supporting information can be downloaded at: https://www.mdpi.com/article/10.3390/cancers17172768/s1, Table S1: A. PCA Analysis in NMIBC; B. PCA Analysis in LGNMIBC; C. PCA Analysis in HG NMIBC; D. PCA analysis according to the EORTC Progression risk scores; E. PCA analysis according to the EORTC Recurrence risk scores; Table S2: A: Let-7c-5p Targets (CSmiRTar output); B: Let-7g-5p Targets (CSmiRTar output); C: miR-9-5p Targets (CSmiRTar output); D: miR-27a-3p Targets (CSmiRTar output); E: miR-143-5p Targets (CSmiRTar output); F: miR-182-5p Targets (CSmiRTar output); G: miR-205-5p Targets (CSmiRTar output); H: miR-369-5p Targets (CSmiRTar output); Table S3: A. Signaling pathways in which the potential targets of Let-7c-5p are involved (identified by DAVID); B. Signaling pathways in which the potential targets of Let-7g-5p are involved (identified by DAVID); C. Signaling pathways in which the potential targets of miR-9-5p are involved (identified by DAVID); D. Signaling pathways in which the potential targets of miR-27a-3p are involved (identified by DAVID); E. Signaling pathways in which the potential targets of miR-27a-3p are involved (identified by DAVID); F. Signaling pathways in which the potential targets of miR-182-5p are involved (identified by DAVID); G. Signaling pathways in which the potential targets of miR-205-5p are involved (identified by DAVID); H. Signaling pathways in which the potential targets of miR-369-5p are involved (identified by DAVID).

## Abbreviations

ANS	Average Normalized Score
BCa	Bladder Cancer
CIS	Carcinoma in situ
CSmiRTar	Condition-Specific miRNA Targets
DAVID	The Database for Annotation, Visualization, and Integrated Discovery
DGIdb	Drug–Gene Interaction Database
EORTC	The European Organization for Research and Treatment of Cancer
FDA	Food and Drug Administration
FFPE	Formalin-Fixed Paraffin-Embedded
HG	High grade
KEGG	The Kyoto Encyclopedia of Genes and Genomes
LG	Low grade
MFS	Metastasis-Free Survival
MIBC	Muscle-Invasive Bladder Cancer
miRNAs	microRNAs
NMIBC	Non-Muscle-Invasive Bladder Cancer
OS	Overall Survival
PCA	Principal Component Analysis
PFS	Progression-Free Survival
TURBT	Transurethral Resection Of The Bladder
