# Supplementary material for: Dysregulated MicroRNAs in Urinary Non-Muscle-Invasive Bladder Cancer: From Molecular Characterization to Clinical Applicability
Source: Cancers (Basel). 2025 Aug 25;17(17):2768. doi: 10.3390/cancers17172768 (PMC12427307; doi:10.3390/cancers17172768)
Supplement: Supplementary file 1 [file cancers-17-02768-s001.zip › cancers-3803082-supplementary.pdf]

Supplementary tables S1

S1.A.PCA Analysis in NMIBC

1- Total variance explained

Component	Initial Eigenvalues			Extraction sums of squared loadings		
	Total	% of variance	% cumulative	Total	% of variance	% cumulative
miR-143	2,025	25,310	25,310	2,025	25,310	25,310
miR-9	1,423	17,781	43,092	1,423	17,781	43,092
miR-182	1,352	16,895	59,987	1,352	16,895	59,987
miR-205	1,102	13,779	73,766	1,102	13,779	73,766
miR-27a	,735	9,188	82,954			
miR-369	,707	8,837	91,791			
Let-7g	,596	7,449	99,240			
Let-7c	,061	,760	100,000			

Extraction Method : Principal Component Analysis

2- Correlation Matrix

		miR-143 (FC)	miR-9 (FC)	miR-182 (FC)	miR-205 (FC)	miR-27a (FC)	miR-369 (FC)	Let-7g (FC)	Let-7c (FC)
Correlation	miR-143 (FC)	1,000	,117	,349	-,045	-,040	-,029	-,140	-,050
	miR-9 (FC)	,117	1,000	-,058	-,110	-,082	,250	-,098	-,056
	miR-182 (FC)	,349	-,058	1,000	-,146	-,107	-,160	-,075	-,061
	miR-205 (FC)	-,045	-,110	-,146	1,000	,933	-,054	-,168	-,086
	miR-27a (FC)	-,040	-,082	-,107	,933	1,000	-,018	-,103	-,060

	miR-369 (FC)	-,029	,250	-,160	-,054	-,018	1,000	-,091	,056
	Let-7g (FC)	-,140	-,098	-,075	-,168	-,103	-,091	1,000	-,142
	Let-7c (FC)	-,050	-,056	-,061	-,086	-,060	,056	-,142	1,000
Significance (unilateral)	miR-143 (FC)		,217	,008	,383	,394	,424	,174	,369
	miR-9 (FC)	,217		,349	,230	,291	,045	,256	,354
	miR-182 (FC)	,008	,349		,164	,237	,141	,309	,342
	miR-205 (FC)	,383	,230	,164		,000	,359	,129	,283
	miR-27a (FC)	,394	,291	,237	,000		,451	,245	,344
	miR-369 (FC)	,424	,045	,141	,359	,451		,272	,353
	Let-7g (FC)	,174	,256	,309	,129	,245	,272		,171
	Let-7c (FC)	,369	,354	,342	,283	,344	,353	,171	

S1.B. PCA Analysis in LG NMIBC

1- Total variance explained

Compnent	Initial Eigenvalues			Extraction sums of squared loadings		
	Total	% of variance	% cumulative	Total	% of variance	% cumulative
miR-9	2,358	29,475	29,475	2,358	29,475	29,475
miR-182	1,733	21,665	51,139	1,733	21,665	51,139
miR-205	1,189	14,859	65,999	1,189	14,859	65,999
miR-143	1,044	13,049	79,048	1,044	13,049	79,048
miR-27a	,729	9,112	88,160			
miR-369	,636	7,953	96,113			
Let-7g	,290	3,625	99,738			
Let-7c	,021	,262	100,000			

Extraction method : Principal Component analysis

2- Correlation Matrix

		miR 9 (FC)	miR-182 (FC)	miR-205 (FC)	miR 143 (FC)	miR-27a (FC)	miR-369 (FC)	Let-7g (FC)	LET 7C (FC)
Correlation	miR-9 (FC)	1,000	,121	-,190	,287	-,191	-,184	,181	-,165
	miR-182 (FC)	,121	1,000	-,187	,543	-,255	-,041	-,141	-,276
	miR-205 (FC)	-,190	-,187	1,000	-,013	,958	-,117	-,259	-,138
	miR-143 (FC)	,287	,543	-,013	1,000	-,150	-,168	,049	-,172
	miR-27a (FC)	-,191	-,255	,958	-,150	1,000	-,016	-,357	-,098
	miR-369 (FC)	-,184	-,041	-,117	-,168	-,016	1,000	,036	,048

	Let-7g (FC)	,181	-,141	-,259	,049	-,357	,036	1,000	-,214
	Let-7c (FC)	-,165	-,276	-,138	-,172	-,098	,048	-,214	1,000
Signification (unilatéral)	miR-9 (FC)		,340	,258	,160	,257	,265	,268	,286
	miR-182 (FC)	,340		,261	,022	,189	,445	,315	,170
	miR-205 (FC)	,258	,261		,483	,000	,345	,186	,319
	miR-143 (FC)	,160	,022	,483		,304	,283	,434	,278
	miR-27a (FC)	,257	,189	,000	,304		,479	,105	,370
	miR-369 (FC)	,265	,445	,345	,283	,479		,452	,435
	Let-7g (FC)	,268	,315	,186	,434	,105	,452		,231
	Let-7c (FC)	,286	,170	,319	,278	,370	,435	,231	

S1.C. PCA Analysis in HG NMIBC

1- Total variance explained

Component	Initial Eigenvalues			Extraction sums of squared loadings		
	Total	% of variance	% cumulative	Total	% of variance	% cumulative
miR-9	1,533	19,164	19,164	1,533	19,164	19,164
miR-182	1,420	17,744	36,908	1,420	17,744	36,908
miR-205	1,206	15,074	51,982	1,206	15,074	51,982
miR-143	1,124	14,054	66,037	1,124	14,054	66,037
miR-27a	,933	11,664	77,701			
miR-369	,710	8,874	86,575			
Let-7g	,618	7,722	94,297			
Let-7c	,456	5,703	100,000			

Extraction method : principal component analysis

2- Correlation matrix

		miR 9 (FC)	miR-182 (FC)	miR-205 (FC)	miR 143 (FC)	miR-27a (FC)	miR-369 (FC)	Let-7g (FC)	LET 7C (FC)
Correlation	miR-9 (FC)	1,000	-,122	-,106	,112	,000	,281	-,141	-,056
	miR-182 (FC)	-,122	1,000	,009	,343	,196	-,163	-,162	-,070
	miR-205 (FC)	-,106	,009	1,000	-,082	,164	-,073	-,203	-,129
	miR-143 (FC)	,112	,343	-,082	1,000	-,083	-,022	-,128	-,049
	miR-27a (FC)	,000	,196	,164	-,083	1,000	-,072	,060	-,088

	miR-369 (FC)	,281	-,163	-,073	-,022	-,072	1,000	-,087	,063
	Let-7g (FC)	-,141	-,162	-,203	-,128	,060	-,087	1,000	-,136
	Let-7c (FC)	-,056	-,070	-,129	-,049	-,088	,063	-,136	1,000
Significance (unilateral)	miR-9 (FC)		,226	,258	,246	,499	,039	,193	,365
	miR-182 (FC)	,226		,478	,015	,112	,158	,159	,335
	miR-205 (FC)	,258	,478		,307	,156	,327	,105	,213
	miR-143 (FC)	,246	,015	,307		,305	,447	,216	,383
	miR-27a (FC)	,499	,112	,156	,305		,330	,356	,295
	miR-369 (FC)	,039	,158	,327	,447	,330		,296	,349
	Let-7g (FC)	,193	,159	,105	,216	,356	,296		,201
	Let-7c (FC)	,365	,335	,213	,383	,295	,349	,201	

S1.D. PCA analysis according to the EORTC Progression risk scores

a- High risk EORTC progression scores

1- Total Variance explained

Component	Initial Eigenvalues			Extraction sums of squared loadings		
	Total	% of variance	% cumulative	Total	% of variance	% cumulative
miR-143	1,477	18,459	18,459	1,477	18,459	18,459
miR-9	1,434	17,925	36,384	1,434	17,925	36,384
miR-182	1,138	14,224	50,608	1,138	14,224	50,608
miR-205	1,119	13,985	64,593	1,119	13,985	64,593
miR-27a	,978	12,225	76,819			
miR-369	,706	8,828	85,647			
Let-7g	,649	8,116	93,763			
Let-7c	,499	6,237	100,000			

2- Correlation Matrix

		miR 143 (FC)	miR 9 (FC)	miR-182 (FC)	miR-205 (FC)	miR-27a (FC)	miR-369 (FC)	Let-7g (FC)	LET 7C (FC)
Correlation	miR-143 (FC)	1,000	,118	,341	-,084	-,081	-,020	-,119	-,044
	miR-9 (FC)	,118	1,000	-,080	-,131	-,002	,284	-,112	-,043
	miR-182 (FC)	,341	-,080	1,000	-,062	,204	-,143	-,129	-,044
	miR-205 (FC)	-,084	-,131	-,062	1,000	,086	-,080	-,139	-,133
	miR-27a (FC)	-,081	-,002	,204	,086	1,000	-,073	,051	-,091
	miR-369 (FC)	-,020	,284	-,143	-,080	-,073	1,000	-,078	,068
	Let-7g (FC)	-,119	-,112	-,129	-,139	,051	-,078	1,000	-,116
	Let-7c (FC)	-,044	-,043	-,044	-,133	-,091	,068	-,116	1,000
Significance (Unilateral)	miR-143 (FC)		,221	,011	,292	,299	,449	,219	,386
	miR-9 (FC)	,221		,300	,196	,496	,029	,232	,390
	miR-182 (FC)	,011	,300		,342	,090	,174	,200	,387
	miR-205 (FC)	,292	,196	,342		,287	,300	,182	,192
	miR-27a (FC)	,299	,496	,090	,287		,317	,371	,275
	miR-369 (FC)	,449	,029	,174	,300	,317		,306	,329

	Let-7g (FC)	,219	,232	,200	,182	,371	,306		,223
	Let-7c (FC)	,386	,390	,387	,192	,275	,329	,223	

- **b-Intermediate risk EORTC progression scores**

1- Total variance explained

Component	Initial Eigenvalues			Extraction sums of squared loadings		
	Total	% of variance	% cumulative	Total	% of variance	% cumulative
miR-143	2,626	32,824	32,824	2,626	32,824	32,824
miR-9	1,701	21,259	54,083	1,701	21,259	54,083
miR-182	1,150	14,375	68,459	1,150	14,375	68,459
miR-205	1,092	13,646	82,105	1,092	13,646	82,105
miR-27a	,807	10,093	92,198			
miR-369	,490	6,119	98,317			
Let-7g	,135	1,683	100,000			
Let-7c	3,776E-5	,000	100,000			

Extraction method : Principal Component Analysis

2- Correlation Matrix

Correlation Matrix

		miR-143 (FC)	miR-9 (FC)	miR-182 (FC)	miR-205 (FC)	miR-27a (FC)	miR-369 (FC)	Let-7g (FC)	Let-7c (FC)
Correlation	miR-143 (FC)	1,000	,151	,507	,001	-,171	-,178	,004	-,192
	miR-9 (FC)	,151	1,000	,037	-,226	-,205	-,173	,140	-,192
	miR-182 (FC)	,507	,037	1,000	-,301	-,406	-,094	,131	-,396
	miR-205 (FC)	,001	-,226	-,301	1,000	,982	-,142	-,461	-,180
	miR-27a (FC)	-,171	-,205	-,406	,982	1,000	-,061	-,454	-,153

	miR-369 (FC)	-,178	-,173	-,094	-,142	-,061	1,000	-,051	,000
	Let-7g (FC)	,004	,140	,131	-,461	-,454	-,051	1,000	-,379
	Let- 7c (FC)	-,192	-,192	-,396	-,180	-,153	,000	-,379	1,000
Significance (unilateral)	miR-143 (FC)		,349	,082	,499	,330	,324	,496	,310
	miR-9 (FC)	,349		,462	,279	,298	,328	,360	,310
	miR-182 (FC)	,082	,462		,216	,139	,405	,368	,146
	miR-205 (FC)	,499	,279	,216		,000	,358	,106	,322
	miR-27a (FC)	,330	,298	,139	,000		,438	,110	,347
	miR-369 (FC)	,324	,328	,405	,358	,438		,448	,500
	Let-7g (FC)	,496	,360	,368	,106	,110	,448		,157
	LET 7C (FC)	,310	,310	,146	,322	,347	,500	,157	

S1.E. PCA analysis according to the EORTC Recurrence risk scores

a- High risk EORTC recurrence scores

1- Total Variance explained

Component	Initial Eigenvalues			Extraction sums of squared loadings		
	Total	% of variance	% cumulative	Total	% of variance	% cumulative
miR-143	3,618	45,220	45,220	3,618	45,220	45,220
miR-9	1,655	20,691	65,911	1,655	20,691	65,911
miR-182	1,224	15,296	81,207	1,224	15,296	81,207
miR-205	,850	10,629	91,835			
miR-27a	,648	8,094	99,930			
miR-369	,006	,070	100,000			
Let-7g	3,421E-16	4,277E-15	100,000			
Let-7c	2,130E-16	2,662E-15	100,000			

Extraction Method : Principal Component Analysis

2- Correlation Matrix[a]

		miR-143 (FC)	miR-9 (FC)	miR-182 (FC)	miR-205 (FC)	miR-27a (FC)	miR-369 (FC)	Let-7g (FC)	Let-7c (FC)
Correlation	miR-143 (FC)	1,000	-,371	-,048	,373	-,280	-,170	,723	-,366
	miR-9 (FC)	-,371	1,000	-,120	-,350	,971	,233	-,283	,991
	miR-182 (FC)	-,048	-,120	1,000	,150	-,300	-,248	-,301	-,231
	miR-205 (FC)	,373	-,350	,150	1,000	-,256	-,396	-,139	-,312
	miR-27a (FC)	-,280	,971	-,300	-,256	1,000	,280	-,228	,986

	miR-369 (FC)	-,170	,233	-,248	-,396	,280	1,000	-,360	,203
	Let-7g (FC)	,723	-,283	-,301	-,139	-,228	-,360	1,000	-,250
	Let-7c (FC)	-,366	,991	-,231	-,312	,986	,203	-,250	1,000

a, This matrix is not defined positive,

- **b-Intermediate risk EORTC recurrence scores**

1- Total Variance explained

Component	Initial Eigenvalues			Extraction sums of squared loadings		
	Total	% of variance	% cumulative	Total	% of variance	% cumulative
miR-9	2,025	25,310	25,310	2,025	25,310	25,310
miR-143	1,423	17,781	43,092	1,423	17,781	43,092
miR-182	1,352	16,895	59,987	1,352	16,895	59,987
miR-205	1,102	13,779	73,766	1,102	13,779	73,766
miR-27a	,735	9,188	82,954			
miR-369	,707	8,837	91,791			
Let-7g	,596	7,449	99,240			
Let-7c	,061	,760	100,000			

Table A: Let-7c-5p Targets (CSmiRTar output)

Species	human
Input Name	hsa-let-7c-5p
Filter Settings	Tissue Filter: **bladder**<br>Disease Filter: **Urothelial Carcinoma**<br>Database Filter >= **2**

29 target genes of the input miRNA (hsa-let-7c-5p)

Target Gene	Normalized Score of hsa-let-7c-5p::target from				ANS	# of supported databases	Validated?
	DIANA-microT	miRanda-,org	miRDB	Target-scan			
CASP3	0,936	0,331	0,417	0,316	0,5	4	
TARBP2	0,838	0,224	0,667	0,147	0,469	4	
RRM2	0,809	0,155	0,571	0,288	0,456	4	↗
CDKN1A	0,693	0,178	0,192	0,172	0,309	4	↗
MIB1	0,547	0,051	0,344	0,09	0,258	4	
RNMT	0,524	0,104	0,18	0,192	0,25	4	
TNFSF10	0,627	0,347		0,318	0,431	3	
TP53	0,635	0,261		0,209	0,368	3	
DUSP1	0,816	0,144		0,132	0,364	3	↗
THBS1	0,564	0,055		0,122	0,247	3	↗
CCND1	0,565	0,01		0,082	0,219	3	↗
DAPK1		0,184	0,193	0,116	0,164	3	
CEBPD	0,411	0,001		0,058	0,157	3	
CXCL8	0,4	0,007		0,053	0,153	3	
HSPA5	0,329	0,002		0,084	0,138	3	

Target Gene	Normalized Score of hsa-let-7c-5p::target from				ANS	# of supported databases	Validated?
	DIANA-microT	miRanda-,org	miRDB	Target-scan			
MAP2K7	0,178	0,027		0,083	0,096	3	
EPHA2	0,18	0,001		0,056	0,079	3	
CYBB	0,147	0,003		0,062	0,071	3	
MTR	0,165	0,001		0	0,055	3	
EZH2	0,218	0,055			0,137	2	
XPO5	0,256	0,004			0,13	2	
IL6		0,047		0,112	0,08	2	
MTOR	0,036	0,062			0,049	2	
MKI67	0,087	0,005			0,046	2	
PTGS2	0,055	0,018			0,037	2	
MMD	0,029	0,028			0,029	2	
FGFR1		0,012		0,026	0,019	2	
AHR		0,016		0,021	0,019	2	
NUMA1	0,015	0,004			0,01	2	

Urothelial Carcinoma
The target genes must be expressed in the selected tissue,
The target genes must be related to the selected disease,
The miRNA::target gene pair must be supported by at least the user-determined number of databases,

Table B: Let-7g-5p Targets (CSmiRTar output)

Species	human
Input Name	hsa-let-7g-5p
Filter Settings	Tissue Filter: **bladder**<br>Disease Filter: **Urothelial Carcinoma**<br>Database Filter >= **2**

30 target genes of the input miRNA (hsa-let-7g-5p)

Target Gene	Normalized Score of hsa-let-7g-5p::target from				ANS	# of supported databases	Validated?
	DIANA-microT	miRanda-,org	miRDB	Target-scan			
CASP3	0,907	0,331	0,417	0,299	0,489	4	↗
TARBP2	0,824	0,224	0,667	0,143	0,465	4	
RRM2	0,809	0,156	0,571	0,28	0,454	4	↗
CDKN1A	0,684	0,178	0,192	0,168	0,306	4	↗
MIB1	0,533	0,051	0,344	0,087	0,254	4	
RNMT	0,5	0,104	0,18	0,187	0,243	4	
TNFSF10	0,587	0,347		0,31	0,415	3	
TP53	0,631	0,262		0,204	0,366	3	
DUSP1	0,733	0,146		0,126	0,335	3	↗
CCND1	0,602	0,01		0,08	0,231	3	↗
THBS1	0,491	0,054		0,117	0,221	3	↗
CEBPD	0,536	0,001		0,054	0,197	3	
DAPK1		0,184	0,193	0,134	0,17	3	
CXCL8	0,427	0,007		0,052	0,162	3	
HSPA5	0,344	0,002		0,082	0,143	3	
MAP2K7	0,176	0,025		0,088	0,096	3	↗

Target Gene	Normalized Score of hsa-let-7g-5p::target from				ANS	# of supported databases	Validated?
	DIANA-microT	miRanda-,org	miRDB	Target-scan			
MTR	0,244	0,001		0	0,082	3	
EPHA2	0,171	0,001		0,057	0,076	3	
CYBB	0,156	0,003		0,06	0,073	3	
EZH2	0,231	0,055			0,143	2	
XPO5	0,273	0,004			0,139	2	
MDM2	0,231	0			0,116	2	
IL6		0,048		0,113	0,081	2	
MTOR	0,064	0,062			0,063	2	
MKI67	0,091	0,005			0,048	2	
MMD	0,067	0,028			0,048	2	
PTGS2	0,058	0,018			0,038	2	
FGFR1		0,012		0,028	0,02	2	
AHR		0,016		0,008	0,012	2	↗
NUMA1	0,016	0,004			0,01	2	

Urothelial Carcinoma
The target genes must be expressed in the selected tissue,
The target genes must be related to the selected disease,
The miRNA::target gene pair must be supported by at least the user-determined number of databases,

Table C: miR-9-5p Targets (CSmiRTar output)

Species	Human
Input Name	hsa-miR-9-5p
Filter Settings	Tissue Filter: **bladder**<br>Disease Filter: **Urothelial Carcinoma**<br>Database Filter >= **2**

38 target genes of the input miRNA (hsa-miR-9-5p)

Target Gene	Normalized Score of hsa-miR-9-5p::target from				ANS	# of supported databases	Validated?
	DIANA-microT	miRanda-,org	miRDB	Target-scan			
LMNA	0,993	0,258	0,495	0,415	0,54	4	↗
EIF4E	0,931	0,371	0,618	0,22	0,535	4	
CCAR2	0,542	0,347	0,823	0,343	0,514	4	
TGFBI	0,035	0,355	0,831	0,534	0,439	4	↗
NFKB1	0,94	0,113	0,513	0,168	0,434	4	↗
MAP2K7	0,462	0,34	0,547	0,11	0,365	4	
ANTXR1	0,371	0,077	0,115	0,148	0,178	4	
ALKBH8	0,464	0,221		0,361	0,349	3	
CYBB	0,431	0,361		0,185	0,326	3	
STMN1	0,733	0,065		0,147	0,315	3	↗
MTR	0,805	0,09		0,039	0,311	3	
EFNA1	0,744	0,022		0,089	0,285	3	↗
EIF5A2	0,565	0,038		0,184	0,262	3	
CD34	0,315	0,26		0,161	0,245	3	↗

Target Gene	Normalized Score of hsa-miR-9-5p::target from				ANS	# of supported databases	Validated?
	DIANA-microT	miRanda-,org	miRDB	Target-scan			
FGFR1	0,171	0,314		0,089	0,191	3	
PTGS2	0,131	0,324		0,087	0,181	3	
SDC1	0,46	0,014		0,017	0,164	3	
EMP2	0,298	0,058		0,119	0,158	3	
PTCH1	0,215	0,003		0,089	0,102	3	
IGF2	0,191	0,008		0,037	0,079	3	
MIB1	0,18	0,001		0,054	0,078	3	
NR3C1	0,156	0		0,047	0,068	3	
DAPK1	0,989	0,064			0,527	2	
DECR1	0,66	0,247			0,454	2	
ATR	0,564	0,044			0,304	2	
MSH2	0,169	0,36			0,265	2	
MET	0,076	0,314			0,195	2	
VEGFA	0,182	0,204			0,193	2	
PIK3CD	0,32	0			0,16	2	
MDM2	0,107	0,126			0,117	2	
BCL2	0,191	0,004			0,098	2	
ALKBH3	0,18	0,01			0,095	2	
THBS1	0,178	0,001			0,09	2	
MMAB	0,138	0,004			0,071	2	
CDC42EP3		0,008		0,091	0,05	2	
TGFB2	0,073	0,007			0,04	2	
CD44	0,067	0,003			0,035	2	

Target Gene	Normalized Score of hsa-miR-9-5p::target from				ANS	# of supported databases	Validated?
	DIANA-microT	miRanda-,org	miRDB	Target-scan			
RNMT		0,001		0	0,001	2	

Urothelial Carcinoma
The target genes must be expressed in the selected tissue,
The miRNA::target gene pair must be supported by at least the user-determined number of databases,
Average Normalized Score
Experimentally validated target supported by miRTarBase

Table D: miR-27a-3p Targets (CSmiRTar output)

Species	Human
Input Name	hsa-miR-27a-3p
Filter Settings	Tissue Filter: **bladder**<br>Disease Filter: **Urothelial Carcinoma**<br>Database Filter >= **2**

49 target genes of the input miRNA (hsa-miR-27a-3p)

Target Gene	Normalized Score of hsa-miR-27a-3p::target from				ANS	# of supported databases	Validated?
	DIANA-microT	miRanda-,org	miRDB	Target-scan			
XPO5	0,511	0,243		0,109	0,288	3	
TP53	0,265	0,17		0,306	0,247	3	
TGFB2	0,182	0,001			0,092	2	
STMN1	0,825	0,115		0,315	0,418	3	
SOD1	0,236	0,03		0,162	0,143	3	
SLTM	0,813	0,068			0,441	2	
SFXN1	0,098	0,012		0,062	0,057	3	
SDC1	0,08	0,015		0,044	0,046	3	
RSF1	0,9	0,038		0,128	0,355	3	
RRM2	0,198	0			0,099	2	
RPS6KB1	0,891	0,136	0,708	0,094	0,457	4	
RNMT	0,727	0,645		0	0,457	3	
PTEN	0,258	0,044			0,151	2	
PTCH1	0,284	0,115		0,225	0,208	3	
PIK3R1	0,604	0,028		0,116	0,249	3	
PIK3CD	0,451	0,004		0,085	0,18	3	

Target Gene	Normalized Score of hsa-miR-27a-3p::target from				ANS	# of supported databases	Validated?
	DIANA-microT	miRanda-,org	miRDB	Target-scan			
NUMA1	0,231	0,004			0,118	2	
NR3C1	0,282	0,271		0,541	0,365	3	
MXI1	0,802	0,312		0,532	0,549	3	
MTR	0,476	0,023		0,195	0,231	3	
MMD	0,995	0,396	0,975	0,51	0,719	4	
MMAB	0,051	0,002		0,04	0,031	3	
MKI67	0,089	0,017		0,167	0,091	3	
MIB1	0,085	0			0,043	2	
MET	0,942	0,203		0,172	0,439	3	
MDM2	0,342	0,007		0	0,116	3	
MAP2K7	0,958	0,031		0,187	0,392	3	
MAD2L1	0,004	0,289			0,147	2	
KRAS	0,851	0,112		0,386	0,45	3	
IGFBP5	0,145	0,002		0,07	0,072	3	
GATA3	0,809	0,374		0,262	0,482	3	
FBLN5	0,862	0,131	0,392	0,253	0,41	4	
EZH2	0,471	0,052			0,262	2	
EPHA2	0,098	0,083		0,159	0,113	3	
EP300		0,002		0,032	0,017	2	
EMP2	0,076	0,009		0,013	0,033	3	
EIF5A2	0,527	0,309	0,423	0,125	0,346	4	
EIF4E	0,2	0,006			0,103	2	
EGFR	0,713	0,272	0,731	0,47	0,547	4	

Target Gene	Normalized Score of hsa-miR-27a-3p::target from				ANS	# of supported databases	Validated?
	DIANA-microT	miRanda-,org	miRDB	Target-scan			
DAPK1	0,027	0,039			0,033	2	
CYBB	0,305	0,103		0,184	0,197	3	
CXCL2	0,233	0,082		0,086	0,134	3	
CDC42EP3	0,924		0,252		0,588	2	
CD44	0,436	0,01		0,013	0,153	3	
CCAR2	0,285	0,001			0,143	2	
ATR	0,1	0,231			0,166	2	
AR	0,22	0,032			0,126	2	
ANTXR1	0,2	0,009			0,105	2	
ADAM9	0,887	0,541		0,128	0,519	3	

Urothelial Carcinoma
The target genes must be expressed in the selected tissue,
The miRNA::target gene pair must be supported by at least the user-determined number of databases,
Average Normalized Score
Experimentally validated target supported by miRTarBase

Table E: miR-143-5p Targets (CSmiRTar output)

Species	human
Input Name	hsa-miR-143-5p
Filter Settings	Tissue Filter: **bladder**<br>Disease Filter: **Urothelial Carcinoma**<br>Database Filter >= 2

24 target genes of the input miRNA (hsa-miR-143-5p)

Target Gene	Normalized Score of hsa-miR-143-5p::target from				ANS	# of supported databases	Validated?
	DIANA-microT	miRanda-,org	miRDB	Target-scan			
HIF1A	0,466	0,417	0,993		0,625	3	
STMN1	0,377	0,429	0,781		0,529	3	
MMD	0,442	0,388	0,016		0,282	3	
RNMT	0,32	0,05	0,036		0,135	3	
PIK3CA	0,87	0,172			0,521	2	
CD34	0,653	0,157			0,405	2	
ANTXR1	0,559	0,22			0,39	2	
EGFR	0,19	0,561			0,376	2	
PIK3CD	0,671	0,042			0,357	2	
HPGDS	0,358	0,12			0,239	2	
TGFB2	0,364	0,032			0,198	2	
SDC1	0,307	0,062			0,185	2	
TSC2	0,349	0			0,175	2	
CYBB	0,285	0,036			0,161	2	
LMNA	0,314	0,006			0,16	2	

Target Gene	Normalized Score of hsa-miR-143-5p::target from				ANS	# of supported databases	Validated?
	DIANA-microT	miRanda-,org	miRDB	Target-scan			
MET	0,044	0,239			0,142	2	
AHR	0,183	0,096			0,14	2	
DAPK1	0,225	0,001			0,113	2	
CASP3	0,143	0,01			0,077	2	
DAB2IP	0,137	0,001			0,069	2	
ATR	0,101	0,011			0,056	2	
AR	0,099	0,01			0,055	2	
FGFR1	0,06	0,008			0,034	2	
ERCC2	0,029	0,001			0,015	2	

Urothelial Carcinoma
The target genes must be expressed in the selected tissue,
The target genes must be related to the selected disease,
The miRNA::target gene pair must be supported by at least the user-determined number of databases,

Table F: miR-182-5p Targets (CSmiRTar output)

Species	Human
Input Name	hsa-miR-182-5p
Filter Settings	Tissue Filter: **bladder**<br>Disease Filter: **Urothelial Carcinoma**<br>Database Filter >= **2**

39 target genes of the input miRNA (hsa-miR-182-5p)

Target Gene	Normalized Score of hsa-miR-182-5p::target from				ANS	# of supported databases	Validated?
	DIANA-microT	miRanda-,org	miRDB	Target-scan			
THBS1	0,851	0,269	0,923	0,292	0,584	4	
ADAM9	0,36	0,295	0,777	0,427	0,465	4	
MMD	0,755	0,354	0,161	0,197	0,367	4	
NR3C1	0,596	0,116	0,039	0,347	0,275	4	
EIF5A2	0,12	0,013	0,682	0,061	0,219	4	
MET	0,978	0,323		0,591	0,631	3	
CEBPA	0,735	0,27		0,267	0,424	3	
BCL2	0,925	0,02		0,312	0,419	3	
PTCH1	0,885	0,211		0,135	0,41	3	
DUSP1	0,429	0,204		0,201	0,278	3	
EP300	0,651	0,044		0,11	0,268	3	
PIK3R1	0,58	0,016		0,142	0,246	3	
DAB2IP	0,647	0,004		0,075	0,242	3	
MTR	0,464	0,007		0,234	0,235	3	
ALKBH8	0,462	0,032		0,199	0,231	3	

Target Gene	Normalized Score of hsa-miR-182-5p::target from				ANS	# of supported databases	Validated?
	DIANA-microT	miRanda-,org	miRDB	Target-scan			
AHR	0,525	0,008		0,138	0,224	3	
TGFB2	0,269	0,101		0,19	0,187	3	
DAB2	0,36	0,009		0,068	0,146	3	
CDC42EP3	0,196	0,089		0,064	0,116	3	
CYBB	0,164	0,012		0,159	0,112	3	
SDC1	0,184	0,043		0,103	0,11	3	
PTGS2	0,176	0,022		0,105	0,101	3	
MMAB	0,122	0,011		0,119	0,084	3	
HSPA5	0,04	0,066		0,119	0,075	3	
LMNA	0,002	0,013		0,129	0,048	3	
MTOR	0,404	0,102			0,253	2	
FHIT		0,16		0,173	0,167	2	
EFNA1	0,209	0,002			0,106	2	
KRAS	0,095	0,106			0,101	2	
CDKN1B	0,136	0,051			0,094	2	
MYC	0,089	0,049			0,069	2	
GSTM2		0,006		0,118	0,062	2	
STMN1		0,061		0,05	0,056	2	
KRT19	0,053	0,018			0,036	2	
MIB1	0,055	0,002			0,029	2	
CXCL8	0,011	0,037			0,024	2	
CCND1	0,04	0,002			0,021	2	
TGFBI	0,016	0,003			0,01	2	

Target Gene	Normalized Score of hsa-miR-182-5p::target from				ANS	# of supported databases	Validated?
	DIANA-microT	miRanda-,org	miRDB	Target-scan			
ELK1	0,009	0,003			0,006	2	

Urothelial Carcinoma
The target genes must be expressed in the selected tissue,
The target genes must be related to the selected disease,
The miRNA::target gene pair must be supported by at least the user-determined number of databases,

Table G: miR-205-5p Targets (CSmiRTar output)

Species	Human
Input Name	hsa-miR-205-5p
Filter Settings	Tissue Filter: **bladder**<br>Disease Filter: **Urothelial Carcinoma**<br>Database Filter >= **2**

42 target genes of the input miRNA (hsa-miR-205-5p)

Target Gene	Normalized Score of hsa-miR-205-5p::target from				ANS	# of supported databases	Validated?
	DIANA-microT	miRanda-,org	miRDB	Target-scan			
MMD	0,882	0,295	0,804	0,278	0,565	4	
VEGFA	0,549	0,335	0,298	0,33	0,378	4	
MTR	0,622	0,057	0,162	0,068	0,227	4	
CYBB	0,767	0,379		0,41	0,519	3	
PTEN	0,885	0,258		0,137	0,427	3	
GATA3	0,742	0,252		0,172	0,389	3	
LMNA	0,611	0,099		0,162	0,291	3	
AHR	0,576	0,018		0,173	0,256	3	
EMP2	0,431	0,048		0,215	0,231	3	
EIF4E	0,587	0,029		0,055	0,224	3	
THBS1	0,531	0,013		0,008	0,184	3	
BCL2	0,373	0,129		0,051	0,184	3	
EFNA1	0,378	0,025		0,086	0,163	3	
ANTXR1	0,156	0,1		0,174	0,143	3	

Target Gene	Normalized Score of hsa-miR-205-5p::target from				ANS	# of supported databases	Validated?
	DIANA-microT	miRanda-,org	miRDB	Target-scan			
TMSB4X	0,315	0,016		0,084	0,138	3	
MMAB	0,345	0,042		0,011	0,133	3	
HMGB1	0,291	0,04		0,031	0,121	3	
MKI67	0,215	0,001		0,08	0,099	3	
FGFR1	0,156	0,077		0,011	0,081	3	
RNMT	0,147	0,04		0,011	0,066	3	
SFXN1	0,078	0,055		0,064	0,066	3	
CDC42EP3	0,095	0,023		0,051	0,056	3	
CEBPA	0,029	0,042		0,015	0,029	3	
MDM2	0,02	0,002		0	0,007	3	
S100A1	0,86	0,308			0,584	2	
SLTM	0,825	0,009			0,417	2	
DAPK1	0,651	0,002			0,327	2	
MSH2		0,302		0,255	0,279	2	
ATR	0,542	0,01			0,276	2	
PTCH1	0,445	0,046			0,246	2	
NR3C1		0,039		0,388	0,214	2	
GSTM2		0,04		0,219	0,13	2	
BOK	0,249	0,002			0,126	2	
EGFR	0,145	0,005			0,075	2	
ELK1	0,102	0,024			0,063	2	
HSPA5	0,105	0,001			0,053	2	
CXCL2		0,002		0,068	0,035	2	

Target Gene	Normalized Score of hsa-miR-205-5p::target from				ANS	# of supported databases	Validated?
	DIANA-microT	miRanda-,org	miRDB	Target-scan			
NUMA1	0,044	0,001			0,023	2	
TGFB2	0,027	0,007			0,017	2	
DAB2IP	0,025	0			0,013	2	
PIK3CD		0,005		0,02	0,013	2	
CASP3		0,01		0,011	0,011	2	

Urothelial Carcinoma
The target genes must be expressed in the selected tissue,
The target genes must be related to the selected disease,
The miRNA::target gene pair must be supported by at least the user-determined number of databases,

Table H: miR-369-5p Targets (CSmiRTar output)

Species	Human
Input Name	hsa-miR-369-5p
Filter Settings	Tissue Filter: **bladder**<br>Disease Filter: **Urothelial Carcinoma**<br>Database Filter >= **2**

4 target genes of the input miRNA (hsa-miR-369-5p)

Target Gene	Normalized Score of hsa-miR-369-5p::target from				ANS	# of supported databases	Validated?
	DIANA-microT	miRanda-,org	miRDB	Target-scan			
TP53	0,22	0,212			0,216	2	
SFXN1	0,015	0,151			0,083	2	
EZH2	0,121	0,033			0,077	2	
KRAS		0,003		0,125	0,064	2	

Urothelial Carcinoma
The target genes must be expressed in the selected tissue,
The miRNA::target gene pair must be supported by at least the user-determined number of databases,
Average Normalized Score
Experimentally validated target supported by miRTarBase

A. Signaling pathways in which the potential targets of Let-7c-5p are involved (identified by DAVID)

Table 1: targets identified by BIOCARTA (DAVID tool)

Table 2: targets identified by KEGG (DAVID tool)

Category	Term	Count	%	PValue	Genes	List Total	Pop Hits	Pop Total	Fold Enrichment	Bonferroni	Benjamini	FDR
**BIOCARTA**	h_ctcfPathway:CTCF: First Multivalent Nuclear Factor	7	12,07	3,25E-06	CDKN1B, TP53, PIK3CA, MDM2, RPS6KB1, MTOR, MYC	31	25	1625	14,68	4,78E-04	4,78E-04	0,0039
**BIOCARTA**	h_p53Pathway:p53 Signaling Pathway	5	8,62	1,91E-04	CDKN1A, CCND1, BCL2, TP53, MDM2	31	17	1625	15,42	0,028	0,0139	0,2265
**BIOCARTA**	h_telPathway:Telomeres, Telomerase, Cellular Aging, and Immortality	5	8,62	2,42E-04	EGFR, KRAS, BCL2, TP53, MYC	31	18	1625	14,56	0,035	0,0118	0,2874
**BIOCARTA**	h_raccycdPathway:Influence of Ras and Rho proteins on G1 to S Transition	5	8,62	0,0012	CDKN1A, CCND1, CDKN1B, PIK3CA, NFKB1	31	27	1625	9,71	0,166	0,0444	1,4595
**BIOCARTA**	h_arfPathway:Tumor Suppressor Arf Inhibits Ribosomal Biogenesis	4	6,90	0,0038	TP53, PIK3CA, MDM2, MYC	31	18	1625	11,65	0,432	0,1071	4,4817
**BIOCARTA**	h_hivnefPathway:HIV-I Nef: negative effector of Fas and TNF	6	10,34	0,0044	NUMA1, BCL2, LMNA, MDM2, NFKB1, MAP2K7	31	61	1625	5,16	0,474	0,1016	5,0724

**BIOCARTA**	h_atmPathway:ATM Signaling Pathway	4	6,90	0,0060	CDKN1A, TP53, MDM2, NFKB1	31	21	1625	9,98	0,5899	0,1194	6,9531
**BIOCARTA**	h_g2Pathway:Cell Cycle: G2/M Checkpoint	4	6,90	0,0099	CDKN1A, PLK1, TP53, MDM2	31	25	1625	8,39	0,7699	0,1676	11,2011
**BIOCARTA**	h_g1Pathway:Cell Cycle: G1/S Check Point	4	6,90	0,0165	CDKN1A, CCND1, CDKN1B, TP53	31	30	1625	6,99	0,9133	0,2377	17,9388
**BIOCARTA**	h_deathPathway:Induction of apoptosis through DR3 and DR4/5 Death Receptors	4	6,90	0,0213	TNFSF10, BCL2, LMNA, NFKB1	31	33	1625	6,35	0,9558	0,2717	22,6323
**BIOCARTA**	h_il2rbPathway:IL-2 Receptor Beta Chain in T cell Activation	4	6,90	0,0332	BCL2, PIK3CA, RPS6KB1, MYC	31	39	1625	5,38	0,9933	0,3629	33,0645
**BIOCARTA**	h_il7Pathway:IL-7 Signal Transduction	3	5,17	0,0377	PTK2B, BCL2, PIK3CA	31	17	1625	9,25	0,9977	0,3758	36,7324
**BIOCARTA**	h_cardiacEGFPathway:Role of EGF Receptor Transactivation by GPCRs in Cardiac Hypertrophy	3	5,17	0,0420	EGFR, NFKB1, MYC	31	18	1625	8,74	0,9988	0,3842	39,9689
**BIOCARTA**	h_igf1mtorPathway:Skeletal muscle hypertrophy is regulated via AKT/mTOR pathway	3	5,17	0,0510	PIK3CA, RPS6KB1, MTOR	31	20	1625	7,86	0,9999	0,4226	46,3329
**BIOCARTA**	h_keratinocytePathway:Keratinocyte Differentiation	4	6,90	0,0562	EGFR, BCL2, NFKB1, MAP2K7	31	48	1625	4,37	0,9999	0,4326	49,7434
**BIOCARTA**	h_her2Pathway:Role of ERBB2 in Signal Transduction and Oncology	3	5,17	0,0656	EGFR, IL6, PIK3CA	31	23	1625	6,84	0,9999	0,4638	55,3898

**BIOCARTA**	h_eif4Pathway:Regulation of eIF4e and p70 S6 Kinase	3	5,17	0,0707	PIK3CA, RPS6KB1, MTOR	31	24	1625	6,55	0,999	0,4698	58,2333
**BIOCARTA**	h_cxcr4Pathway:CXCR4 Signaling Pathway	3	5,17	0,0707	PTK2B, PIK3CA, NFKB1	31	24	1625	6,55	0,999	0,4698	58,2333
**BIOCARTA**	h_nthiPathway:NFkB activation by Nontypeable Hemophilus influenzae	3	5,17	0,0707	DUSP1, CXCL8, NFKB1	31	24	1625	6,55	0,999	0,4698	58,2333
**BIOCARTA**	h_cellcyclePathway:Cyclins and Cell Cycle Regulation	3	5,17	0,0760	CDKN1A, CCND1, CDKN1B	31	25	1625	6,29	0,999	0,4757	60,9754
**BIOCARTA**	h_mtorPathway:mTOR Signaling Pathway	3	5,17	0,0814	PIK3CA, RPS6KB1, MTOR	31	26	1625	6,05	0,999	0,4817	63,6101
**BIOCARTA**	h_p53hypoxiaPathway:Hypoxia and p53 in the Cardiovascular system	3	5,17	0,0814	CDKN1A, TP53, MDM2	31	26	1625	6,05	0,999	0,4817	63,6101
**BIOCARTA**	H_gsk3Pathway:Inactivation of Gsk3 by AKT causes accumulation of b-catenin in Alveolar Macrophages	3	5,17	0,0870	CCND1, PIK3CA, NFKB1	31	27	1625	5,82	0,999	0,4877	66,1330

Category	Term	Count	%	P Value	Genes	List Total	Pop Hits	Pop Total	Fold Enrichment	Bonferroni	Benjamini	FDR
**KEGG_PATHWAY**	hsa05219:Bladder cancer	10	17,24	8,89E-13	EGFR, CDKN1A, CCND1, KRAS, TP53, CXCL8, MDM2, THBS1, MYC, DAPK1	41	41	6879	40,92	1,33E-10	1,33E-10	1,06E-09
**KEGG_PATHWAY**	hsa05215:Prostate cancer	12	20,69	1,35E-12	EGFR, FGFR1, CDKN1A, CCND1, CDKN1B, KRAS, BCL2, TP53, PIK3CA, MDM2, NFKB1, MTOR	41	88	6879	22,88	2,03E-10	1,01E-10	1,61E-09
**KEGG_PATHWAY**	hsa05205:Proteoglycans in cancer	15	25,86	2,34E-12	EGFR, FGFR1, TP53, IGF2, RPS6KB1, CDKN1A, CCND1, KRAS, CD44, MDM2, PIK3CA, PTCH1, MTOR, THBS1, MYC	41	200	6879	12,58	3,51E-10	1,17E-10	2,79E-09
**KEGG_PATHWAY**	hsa05200:Pathways in cancer	18	31,03	1,38E-11	EGFR, FGFR1, IL6, PTGS2, TP53, CXCL8, NFKB1, DAPK1, CDKN1A, CCND1, KRAS, CDKN1B, BCL2, MDM2, PIK3CA, PTCH1, MTOR, MYC	41	393	6879	7,68	2,07E-09	5,18E-10	1,65E-08
**KEGG_PATHWAY**	hsa04151:PI3K-Akt signaling pathway	17	29,31	2,38E-11	EGFR, FGFR1, IL6, TP53, NFKB1, RPS6KB1, EPHA2, CDKN1A, CCND1, KRAS, CDKN1B, BCL2, MDM2, PIK3CA, MTOR, THBS1, MYC	41	345	6879	8,27	3,57E-09	7,13E-10	2,84E-08
**KEGG_PATHWAY**	hsa05206:MicroRNAs in cancer	15	25,86	2,95E-10	EGFR, PTGS2, EZH2, TP53, NFKB1, CDKN1A, CCND1, CDKN1B, KRAS, CD44, BCL2, MDM2, MTOR, THBS1, MYC	41	286	6879	8,80	4,42E-08	7,37E-09	3,52E-07

**KEGG_PATHWAY**	hsa05161:Hepatitis B	12	20,69	3,42E-10	CDKN1A, IL6, CCND1, CDKN1B, KRAS, PTK2B, BCL2, TP53, CXCL8, PIK3CA, NFKB1, MYC	41	145	6879	13,89	5,12E-08	7,32E-09	4,08E-07
**KEGG_PATHWAY**	hsa05220:Chronic myeloid leukemia	9	15,52	5,70E-09	CDKN1A, CCND1, CDKN1B, KRAS, TP53, PIK3CA, MDM2, NFKB1, MYC	41	72	6879	20,97	8,54E-07	1,07E-07	6,80E-06
**KEGG_PATHWAY**	hsa05222:Small cell lung cancer	9	15,52	2,17E-08	FHIT, CCND1, CDKN1B, PTGS2, BCL2, TP53, PIK3CA, NFKB1, MYC	41	85	6879	17,76	3,25E-06	3,62E-07	2,59E-05
**KEGG_PATHWAY**	hsa05169:Epstein-Barr virus infection	10	17,24	2,22E-08	CDKN1A, CDKN1B, CD44, BCL2, TP53, PIK3CA, MDM2, NFKB1, MAP2K7, MYC	41	122	6879	13,75	3,33E-06	3,33E-07	2,65E-05
**KEGG_PATHWAY**	hsa04012:ErbB signaling pathway	9	15,52	2,61E-08	EGFR, CDKN1A, CDKN1B, KRAS, PIK3CA, RPS6KB1, MTOR, MAP2K7, MYC	41	87	6879	17,36	3,92E-06	3,56E-07	3,12E-05
**KEGG_PATHWAY**	hsa04068:FoxO signaling pathway	10	17,24	5,06E-08	EGFR, CDKN1A, IL6, TNFSF10, CCND1, CDKN1B, KRAS, PLK1, PIK3CA, MDM2	41	134	6879	12,52	7,59E-06	6,32E-07	6,04E-05
**KEGG_PATHWAY**	hsa04066:HIF-1 signaling pathway	9	15,52	5,71E-08	EGFR, CDKN1A, IL6, CDKN1B, BCL2, PIK3CA, NFKB1, RPS6KB1, MTOR	41	96	6879	15,73	8,56E-06	6,59E-07	6,82E-05
**KEGG_PAT**	hsa05214:Glioma	8	13,	7,05E	EGFR, CDKN1A, CCND1, KRAS, TP53, PIK3CA, MDM2, MTOR	41	65	6879	20,65	1,06E-05	7,55E-07	8,42E

HWAY			79	-08								-05
KEGG_PATHWAY	hsa05218:Melanoma	8	13,79	1,31E-07	EGFR, FGFR1, CDKN1A, CCND1, KRAS, TP53, PIK3CA, MDM2	41	71	6879	18,90	1,97E-05	1,31E-06	1,57E-04
KEGG_PATHWAY	hsa05221:Acute myeloid leukemia	7	12,07	6,86E-07	CCND1, KRAS, PIK3CA, NFKB1, RPS6KB1, MTOR, MYC	41	56	6879	20,97	1,03E-04	6,43E-06	8,19E-04
KEGG_PATHWAY	hsa05230:Central carbon metabolism in cancer	7	12,07	1,53E-06	EGFR, FGFR1, KRAS, TP53, PIK3CA, MTOR, MYC	41	64	6879	18,35	2,30E-04	1,35E-05	0,0018
KEGG_PATHWAY	hsa05213:Endometrial cancer	6	10,34	1,09E-05	EGFR, CCND1, KRAS, TP53, PIK3CA, MYC	41	52	6879	19,36	0,002	9,10E-05	0,0130
KEGG_PATHWAY	hsa05223:Non-small cell lung cancer	6	10,34	1,58E-05	EGFR, FHIT, CCND1, KRAS, TP53, PIK3CA	41	56	6879	17,98	0,002	1,25E-04	0,0188
KEGG_PATHWAY	hsa05210:Colorectal cancer	6	10,34	2,61E-05	CCND1, KRAS, BCL2, TP53, PIK3CA, MYC	41	62	6879	16,24	0,004	1,96E-04	0,0311
KEGG_PATHWAY	hsa05212:Pancreatic cancer	6	10,34	3,29E-05	EGFR, CCND1, KRAS, TP53, PIK3CA, NFKB1	41	65	6879	15,49	0,005	2,35E-04	0,0392

**KEGG_PATHWAY**	hsa04115:p53 signaling pathway	6	10,34	3,81E-05	CDKN1A, CCND1, RRM2, TP53, MDM2, THBS1	41	67	6879	15,03	0,006	2,60E-04	0,0455
**KEGG_PATHWAY**	hsa05202:Transcriptional misregulation in cancer	8	13,79	4,16E-05	CDKN1A, IL6, CDKN1B, TP53, CXCL8, MDM2, NFKB1, MYC	41	167	6879	8,04	0,006	2,71E-04	0,0496
**KEGG_PATHWAY**	hsa04919:Thyroid hormone signaling pathway	7	12,07	4,62E-05	CCND1, KRAS, TP53, PIK3CA, MDM2, MTOR, MYC	41	115	6879	10,21	0,007	2,89E-04	0,0552
**KEGG_PATHWAY**	hsa04110:Cell cycle	7	12,07	7,06E-05	CDKN1A, CCND1, CDKN1B, PLK1, TP53, MDM2, MYC	41	124	6879	9,47	0,011	4,24E-04	0,0843
**KEGG_PATHWAY**	hsa05162:Measles	7	12,07	1,04E-04	IL6, TNFSF10, CCND1, CDKN1B, TP53, PIK3CA, NFKB1	41	133	6879	8,83	0,016	6,02E-04	0,1245
**KEGG_PATHWAY**	hsa05160:Hepatitis C	7	12,07	1,04E-04	EGFR, CDKN1A, KRAS, TP53, CXCL8, PIK3CA, NFKB1	41	133	6879	8,83	0,016	6,02E-04	0,1245
**KEGG_PATHWAY**	hsa05203:Viral carcinogenesis	8	13,79	1,52E-04	CDKN1A, CCND1, CDKN1B, KRAS, TP53, PIK3CA, MDM2, NFKB1	41	205	6879	6,55	0,023	8,45E-04	0,1816
**KEGG_PAT**	hsa04210:Apoptosis	5	8,62	4,29E	TNFSF10, BCL2, TP53, PIK3CA, NFKB1	41	62	6879	13,53	0,062	0,0023	0,5110

**HWAY**				-04								
**KEGG_PATHWAY**	hsa04010:MAPK signaling pathway	8	13,79	5,52E-04	EGFR, FGFR1, KRAS, DUSP1, TP53, NFKB1, MAP2K7, MYC	41	253	6879	5,31	0,079	0,0029	0,6568
**KEGG_PATHWAY**	hsa05166:HTLV-I infection	8	13,79	5,65E-04	CDKN1A, IL6, CCND1, KRAS, TP53, PIK3CA, NFKB1, MYC	41	254	6879	5,28	0,081	0,0028	0,6725
**KEGG_PATHWAY**	hsa05216:Thyroid cancer	4	6,90	5,99E-04	CCND1, KRAS, TP53, MYC	41	29	6879	23,14	0,086	0,0029	0,7134
**KEGG_PATHWAY**	hsa04722:Neurotrophin signaling pathway	6	10,34	6,00E-04	KRAS, BCL2, TP53, PIK3CA, NFKB1, MAP2K7	41	120	6879	8,39	0,086	0,0028	0,7142
**KEGG_PATHWAY**	hsa05231:Choline metabolism in cancer	5	8,62	0,0027	EGFR, KRAS, PIK3CA, RPS6KB1, MTOR	41	101	6879	8,31	0,330	0,0121	3,1389
**KEGG_PATHWAY**	hsa05164:Influenza A	6	10,34	0,0031	IL6, TNFSF10, CXCL8, PIK3CA, NFKB1, MAP2K7	41	174	6879	5,79	0,376	0,0138	3,6855
**KEGG_PATHWAY**	hsa04620:Toll-like receptor signaling pathway	5	8,62	0,0032	IL6, CXCL8, PIK3CA, NFKB1, MAP2K7	41	106	6879	7,91	0,380	0,0135	3,7299

**KEGG_PATHWAY**	hsa04668:TNF signaling pathway	5	8,62	0,0033	IL6, PTGS2, PIK3CA, NFKB1, MAP2K7	41	107	6879	7,84	0,390	0,0136	3,8563
**KEGG_PATHWAY**	hsa04931:Insulin resistance	5	8,62	0,0034	IL6, PIK3CA, NFKB1, RPS6KB1, MTOR	41	108	6879	7,77	0,400	0,0137	3,9856
**KEGG_PATHWAY**	hsa04071:Sphingolipid signaling pathway	5	8,62	0,0050	KRAS, BCL2, TP53, PIK3CA, NFKB1	41	120	6879	6,99	0,525	0,0194	5,7621
**KEGG_PATHWAY**	hsa04015:Rap1 signaling pathway	6	10,34	0,0070	EGFR, FGFR1, KRAS, PIK3CA, THBS1, EPHA2	41	210	6879	4,79	0,650	0,0265	8,0136
**KEGG_PATHWAY**	hsa04917:Prolactin signaling pathway	4	6,90	0,0079	CCND1, KRAS, PIK3CA, NFKB1	41	71	6879	9,45	0,697	0,0294	9,0601
**KEGG_PATHWAY**	hsa04014:Ras signaling pathway	6	10,34	0,0094	EGFR, FGFR1, KRAS, PIK3CA, NFKB1, EPHA2	41	226	6879	4,45	0,759	0,0341	10,7031
**KEGG_PATHWAY**	hsa04921:Oxytocin signaling pathway	5	8,62	0,0108	EGFR, CDKN1A, CCND1, KRAS, PTGS2	41	150	6879	5,59	0,804	0,0380	12,1540
**KEGG_PAT**	hsa04064:NF-kappa B signaling pathway	4	6,90	0,0138	PTGS2, BCL2, CXCL8, NFKB1	41	87	6879	7,71	0,875	0,0473	15,2713

**HWAY**												
**KEGG_PATHWAY**	hsa04912:GnRH signaling pathway	4	6,90	0,0155	EGFR, KRAS, PTK2B, MAP2K7	41	91	6879	7,37	0,905	0,0520	17,0636
**KEGG_PATHWAY**	hsa04660:T cell receptor signaling pathway	4	6,90	0,0200	KRAS, PIK3CA, NFKB1, MAP2K7	41	100	6879	6,71	0,951	0,0650	21,4007
**KEGG_PATHWAY**	hsa05142:Chagas disease (American trypanosomiasis)	4	6,90	0,0221	IL6, CXCL8, PIK3CA, NFKB1	41	104	6879	6,45	0,965	0,0704	23,4488
**KEGG_PATHWAY**	hsa04062:Chemokine signaling pathway	5	8,62	0,0222	KRAS, PTK2B, CXCL8, PIK3CA, NFKB1	41	186	6879	4,51	0,965	0,0690	23,4710
**KEGG_PATHWAY**	hsa05146:Amoebiasis	4	6,90	0,0233	IL6, CXCL8, PIK3CA, NFKB1	41	106	6879	6,33	0,971	0,0709	24,4973
**KEGG_PATHWAY**	hsa04510:Focal adhesion	5	8,62	0,0308	EGFR, CCND1, BCL2, PIK3CA, THBS1	41	206	6879	4,07	0,991	0,0913	31,1580
**KEGG_PATHWAY**	hsa05144:Malaria	3	5,17	0,0326	IL6, CXCL8, THBS1	41	49	6879	10,27	0,993	0,0948	32,7237

**KEGG_PATHWAY**	hsa04650:Natural killer cell mediated cytotoxicity	4	6,90	0,0334	TNFSF10, KRAS, PTK2B, PIK3CA	41	122	6879	5,50	0,994	0,0951	33,3492
**KEGG_PATHWAY**	hsa04152:AMPK signaling pathway	4	6,90	0,0341	CCND1, PIK3CA, RPS6KB1, MTOR	41	123	6879	5,46	0,995	0,0952	33,9232
**KEGG_PATHWAY**	hsa05134:Legionellosis	3	5,17	0,0390	IL6, CXCL8, NFKB1	41	54	6879	9,32	0,997	0,1065	37,8242
**KEGG_PATHWAY**	hsa04380:Osteoclast differentiation	4	6,90	0,0400	CYBB, PIK3CA, NFKB1, MAP2K7	41	131	6879	5,12	0,998	0,1071	38,5649
**KEGG_PATHWAY**	hsa04621:NOD-like receptor signaling pathway	3	5,17	0,0417	IL6, CXCL8, NFKB1	41	56	6879	8,99	0,998	0,1096	39,8551
**KEGG_PATHWAY**	hsa04150:mTOR signaling pathway	3	5,17	0,0444	PIK3CA, RPS6KB1, MTOR	41	58	6879	8,68	0,998	0,1146	41,8740
**KEGG_PATHWAY**	hsa04910:Insulin signaling pathway	4	6,90	0,0455	KRAS, PIK3CA, RPS6KB1, MTOR	41	138	6879	4,86	0,999	0,1154	42,6648
**KEGG_PAT**	hsa04550:Signaling pathways regulating	4	6,90	0,0472	FGFR1, KRAS, PIK3CA, MYC	41	140	6879	4,79	0,999	0,1175	43,8365

HWAY	pluripotency of stem cells											
KEGG_PATHWAY	hsa04370:VEGF signaling pathway	3	5,17	0,0486	KRAS, PTGS2, PIK3CA	41	61	6879	8,25	0,999	0,1191	44,8705
KEGG_PATHWAY	hsa04630:Jak-STAT signaling pathway	4	6,90	0,0514	IL6, CCND1, PIK3CA, MYC	41	145	6879	4,63	0,999	0,1236	46,7570
KEGG_PATHWAY	hsa05131:Shigellosis	3	5,17	0,0530	CD44, CXCL8, NFKB1	41	64	6879	7,86	0,999	0,1254	47,8177
KEGG_PATHWAY	hsa04932:Non-alcoholic fatty liver disease (NAFLD)	4	6,90	0,0568	IL6, CXCL8, PIK3CA, NFKB1	41	151	6879	4,44	0,999	0,1318	50,2290
KEGG_PATHWAY	hsa05120:Epithelial cell signaling in Helicobacter pylori infection	3	5,17	0,0575	EGFR, CXCL8, NFKB1	41	67	6879	7,51	0,999	0,1315	50,7045
KEGG_PATHWAY	hsa04664:Fc epsilon RI signaling pathway	3	5,17	0,0590	KRAS, PIK3CA, MAP2K7	41	68	6879	7,40	0,999	0,1329	51,6515
KEGG_PATHWAY	hsa04662:B cell receptor signaling pathway	3	5,17	0,0606	KRAS, PIK3CA, NFKB1	41	69	6879	7,29	0,999	0,1343	52,5904

**KEGG_PATHWAY**	hsa05133:Pertussis	3	5,17	0,0701	IL6, CXCL8, NFKB1	41	75	6879	6,71	0,999	0,1523	58,0362
**KEGG_PATHWAY**	hsa05204:Chemical carcinogenesis	3	5,17	0,0784	PTGS2, SULT1A1, GSTO1	41	80	6879	6,29	0,999	0,1671	62,3025
**KEGG_PATHWAY**	hsa05132:Salmonella infection	3	5,17	0,0836	IL6, CXCL8, NFKB1	41	83	6879	6,06	0,999	0,1751	64,7334
**KEGG_PATHWAY**	hsa04350:TGF-beta signaling pathway	3	5,17	0,0853	RPS6KB1, THBS1, MYC	41	84	6879	5,99	0,999	0,1762	65,5215
**KEGG_PATHWAY**	hsa04914:Progesterone-mediated oocyte maturation	3	5,17	0,0906	KRAS, PLK1, PIK3CA	41	87	6879	5,79	0,999	0,1841	67,8176

B. Signaling pathways in which the potential targets of Let-7g-5p are involved (identified by DAVID)

Table 1: targets identified by BIOCARTA (DAVID tool)

Table 2: targets identified by KEGG (DAVID tool)

Category	Term	Count	%	PValue	Genes	List Total	Pop Hits	Pop Total	Fold Enrichment	Bonferroni	Benjamini	FDR
**BIOCARTA**	h_ctcfPathway:CTCF: First Multivalent Nuclear Factor	7	12,07	3,25E-06	CDKN1B, TP53, PIK3CA, MDM2, RPS6KB1, MTOR, MYC	31	25	1625	14,68	4,78E-04	4,78E-04	0,0039
**BIOCARTA**	h_p53Pathway:p53 Signaling Pathway	5	8,62	1,91E-04	CDKN1A, CCND1, BCL2, TP53, MDM2	31	17	1625	15,42	0,028	0,0139	0,2265
**BIOCARTA**	h_telPathway:Telomeres, Telomerase, Cellular Aging, and Immortality	5	8,62	2,42E-04	EGFR, KRAS, BCL2, TP53, MYC	31	18	1625	14,56	0,035	0,0118	0,2874
**BIOCARTA**	h_raccycdPathway:Influence of Ras and Rho proteins on G1 to S Transition	5	8,62	0,0012	CDKN1A, CCND1, CDKN1B, PIK3CA, NFKB1	31	27	1625	9,71	0,166	0,0444	1,4595
**BIOCARTA**	h_arfPathway:Tumor Suppressor Arf Inhibits Ribosomal Biogenesis	4	6,90	0,0038	TP53, PIK3CA, MDM2, MYC	31	18	1625	11,65	0,432	0,1071	4,4817
**BIOCARTA**	h_hivnefPathway:HIV-I Nef: negative effector of Fas and TNF	6	10,	0,0044	NUMA1, BCL2, LMNA, MDM2, NFKB1, MAP2K7	31	61	1625	5,16	0,474	0,1016	5,0724

			34									
**BIOCARTA**	h_atmPathway:ATM Signaling Pathway	4	6,90	0,0060	CDKN1A, TP53, MDM2, NFKB1	31	21	1625	9,98	0,589	0,1194	6,9531
**BIOCARTA**	h_g2Pathway:Cell Cycle: G2/M Checkpoint	4	6,90	0,0099	CDKN1A, PLK1, TP53, MDM2	31	25	1625	8,39	0,769	0,1676	11,2011
**BIOCARTA**	h_g1Pathway:Cell Cycle: G1/S Check Point	4	6,90	0,0165	CDKN1A, CCND1, CDKN1B, TP53	31	30	1625	6,99	0,913	0,2377	17,9388
**BIOCARTA**	h_deathPathway:Induction of apoptosis through DR3 and DR4/5 Death Receptors	4	6,90	0,0213	TNFSF10, BCL2, LMNA, NFKB1	31	33	1625	6,35	0,958	0,2717	22,6323
**BIOCARTA**	h_il2rbPathway:IL-2 Receptor Beta Chain in T cell Activation	4	6,90	0,0332	BCL2, PIK3CA, RPS6KB1, MYC	31	39	1625	5,38	0,993	0,3629	33,0645
**BIOCARTA**	h_il7Pathway:IL-7 Signal Transduction	3	5,17	0,0377	PTK2B, BCL2, PIK3CA	31	17	1625	9,25	0,997	0,3758	36,7324
**BIOCARTA**	h_cardiacEGFPathway:Role of EGF Receptor Transactivation by GPCRs in Cardiac Hypertrophy	3	5,17	0,0420	EGFR, NFKB1, MYC	31	18	1625	8,74	0,998	0,3842	39,9689
**BIOCARTA**	h_igf1mtorPathway:Skeletal muscle hypertrophy is regulated via AKT/mTOR pathway	3	5,17	0,0510	PIK3CA, RPS6KB1, MTOR	31	20	1625	7,86	0,999	0,4226	46,3329
**BIOCARTA**	h_keratinocytePathway:Keratinocyte Differentiation	4	6,90	0,0562	EGFR, BCL2, NFKB1, MAP2K7	31	48	1625	4,37	0,999	0,4326	49,7434

**BIOCARTA**	h_her2Pathway:Role of ERBB2 in Signal Transduction and Oncology	3	5,17	0,0656	EGFR, IL6, PIK3CA	31	23	1625	6,84	0,999	0,4638	55,3898
**BIOCARTA**	h_eif4Pathway:Regulation of eIF4e and p70 S6 Kinase	3	5,17	0,0707	PIK3CA, RPS6KB1, MTOR	31	24	1625	6,55	0,999	0,4698	58,2333
**BIOCARTA**	h_cxcr4Pathway:CXCR4 Signaling Pathway	3	5,17	0,0707	PTK2B, PIK3CA, NFKB1	31	24	1625	6,55	0,999	0,4698	58,2333
**BIOCARTA**	h_nthiPathway:NFkB activation by Nontypeable Hemophilus influenzae	3	5,17	0,0707	DUSP1, CXCL8, NFKB1	31	24	1625	6,55	0,999	0,4698	58,2333
**BIOCARTA**	h_cellcyclePathway:Cyclins and Cell Cycle Regulation	3	5,17	0,0760	CDKN1A, CCND1, CDKN1B	31	25	1625	6,29	0,999	0,4757	60,9754
**BIOCARTA**	h_mtorPathway:mTOR Signaling Pathway	3	5,17	0,0814	PIK3CA, RPS6KB1, MTOR	31	26	1625	6,05	0,999	0,4817	63,6101
**BIOCARTA**	h_p53hypoxiaPathway:Hypoxia and p53 in the Cardiovascular system	3	5,17	0,0814	CDKN1A, TP53, MDM2	31	26	1625	6,05	0,999	0,4817	63,6101
**BIOCARTA**	H_gsk3Pathway:Inactivation of Gsk3 by AKT causes accumulation of b-catenin in Alveolar Macrophages	3	5,17	0,0870	CCND1, PIK3CA, NFKB1	31	27	1625	5,82	0,999	0,4877	66,1330

Category	Term	Count	%	P Value	Genes	List Total	Pop Hits	Pop Total	Fold Enrichment	Bonferroni	Benjamini	FDR
**KEGG_PATHWAY**	hsa05219:Bladder cancer	10	17,24	8,89E-13	EGFR, CDKN1A, CCND1, KRAS, TP53, CXCL8, MDM2, THBS1, MYC, DAPK1	41	41	6879	40,92	1,33E-10	1,33E-10	1,06E-09
**KEGG_PATHWAY**	hsa05215:Prostate cancer	12	20,69	1,35E-12	EGFR, FGFR1, CDKN1A, CCND1, CDKN1B, KRAS, BCL2, TP53, PIK3CA, MDM2, NFKB1, MTOR	41	88	6879	22,88	2,03E-10	1,01E-10	1,61E-09
**KEGG_PATHWAY**	hsa05205:Proteoglycans in cancer	15	25,86	2,34E-12	EGFR, FGFR1, TP53, IGF2, RPS6KB1, CDKN1A, CCND1, KRAS, CD44, MDM2, PIK3CA, PTCH1, MTOR, THBS1, MYC	41	200	6879	12,58	3,51E-10	1,17E-10	2,79E-09
**KEGG_PATHWAY**	hsa05200:Pathways in cancer	18	31,03	1,38E-11	EGFR, FGFR1, IL6, PTGS2, TP53, CXCL8, NFKB1, DAPK1, CDKN1A, CCND1, KRAS, CDKN1B, BCL2, MDM2, PIK3CA, PTCH1, MTOR, MYC	41	393	6879	7,68	2,07E-09	5,18E-10	1,65E-08
**KEGG_PATHWAY**	hsa04151:PI3K-Akt signaling pathway	17	29,31	2,38E-11	EGFR, FGFR1, IL6, TP53, NFKB1, RPS6KB1, EPHA2, CDKN1A, CCND1, KRAS, CDKN1B, BCL2, MDM2, PIK3CA, MTOR, THBS1, MYC	41	345	6879	8,27	3,57E-09	7,13E-10	2,84E-08
**KEGG_PATHWAY**	hsa05206:MicroRNAs in cancer	15	25,86	2,95E-10	EGFR, PTGS2, EZH2, TP53, NFKB1, CDKN1A, CCND1, CDKN1B, KRAS, CD44, BCL2, MDM2, MTOR, THBS1, MYC	41	286	6879	8,80	4,42E-08	7,37E-09	3,52E-07
**KEGG_PAT**	hsa05161:Hepatitis B	12	20,	3,42E	CDKN1A, IL6, CCND1, CDKN1B, KRAS, PTK2B, BCL2, TP53, CXCL8, PIK3CA, NFKB1, MYC	41	145	6879	13,89	5,12E-08	7,32E-09	4,08E

**HWAY**			69	-10								-07
**KEGG_PATHWAY**	hsa05220:Chronic myeloid leukemia	9	15,52	5,70E-09	CDKN1A, CCND1, CDKN1B, KRAS, TP53, PIK3CA, MDM2, NFKB1, MYC	41	72	6879	20,97	8,54E-07	1,07E-07	6,80E-06
**KEGG_PATHWAY**	hsa05222:Small cell lung cancer	9	15,52	2,17E-08	FHIT, CCND1, CDKN1B, PTGS2, BCL2, TP53, PIK3CA, NFKB1, MYC	41	85	6879	17,76	3,25E-06	3,62E-07	2,59E-05
**KEGG_PATHWAY**	hsa05169:Epstein-Barr virus infection	10	17,24	2,22E-08	CDKN1A, CDKN1B, CD44, BCL2, TP53, PIK3CA, MDM2, NFKB1, MAP2K7, MYC	41	122	6879	13,75	3,33E-06	3,33E-07	2,65E-05
**KEGG_PATHWAY**	hsa04012:ErbB signaling pathway	9	15,52	2,61E-08	EGFR, CDKN1A, CDKN1B, KRAS, PIK3CA, RPS6KB1, MTOR, MAP2K7, MYC	41	87	6879	17,36	3,92E-06	3,56E-07	3,12E-05
**KEGG_PATHWAY**	hsa04068:FoxO signaling pathway	10	17,24	5,06E-08	EGFR, CDKN1A, IL6, TNFSF10, CCND1, CDKN1B, KRAS, PLK1, PIK3CA, MDM2	41	134	6879	12,52	7,59E-06	6,32E-07	6,04E-05
**KEGG_PATHWAY**	hsa04066:HIF-1 signaling pathway	9	15,52	5,71E-08	EGFR, CDKN1A, IL6, CDKN1B, BCL2, PIK3CA, NFKB1, RPS6KB1, MTOR	41	96	6879	15,73	8,56E-06	6,59E-07	6,82E-05
**KEGG_PATHWAY**	hsa05214:Glioma	8	13,79	7,05E-08	EGFR, CDKN1A, CCND1, KRAS, TP53, PIK3CA, MDM2, MTOR	41	65	6879	20,65	1,06E-05	7,55E-07	8,42E-05

**KEGG_PATHWAY**	hsa05218:Melanoma	8	13,79	1,31E-07	EGFR, FGFR1, CDKN1A, CCND1, KRAS, TP53, PIK3CA, MDM2	41	71	6879	18,90	1,97E-05	1,31E-06	1,57E-04
**KEGG_PATHWAY**	hsa05221:Acute myeloid leukemia	7	12,07	6,86E-07	CCND1, KRAS, PIK3CA, NFKB1, RPS6KB1, MTOR, MYC	41	56	6879	20,97	1,03E-04	6,43E-06	8,19E-04
**KEGG_PATHWAY**	hsa05230:Central carbon metabolism in cancer	7	12,07	1,53E-06	EGFR, FGFR1, KRAS, TP53, PIK3CA, MTOR, MYC	41	64	6879	18,35	2,30E-04	1,35E-05	0,0018
**KEGG_PATHWAY**	hsa05213:Endometrial cancer	6	10,34	1,09E-05	EGFR, CCND1, KRAS, TP53, PIK3CA, MYC	41	52	6879	19,36	0,002	9,10E-05	0,0130
**KEGG_PATHWAY**	hsa05223:Non-small cell lung cancer	6	10,34	1,58E-05	EGFR, FHIT, CCND1, KRAS, TP53, PIK3CA	41	56	6879	17,98	0,002	1,25E-04	0,0188
**KEGG_PATHWAY**	hsa05210:Colorectal cancer	6	10,34	2,61E-05	CCND1, KRAS, BCL2, TP53, PIK3CA, MYC	41	62	6879	16,24	0,004	1,96E-04	0,0311
**KEGG_PATHWAY**	hsa05212:Pancreatic cancer	6	10,34	3,29E-05	EGFR, CCND1, KRAS, TP53, PIK3CA, NFKB1	41	65	6879	15,49	0,005	2,35E-04	0,0392
**KEGG_PAT**	hsa04115:p53 signaling pathway	6	10,	3,81E	CDKN1A, CCND1, RRM2, TP53, MDM2, THBS1	41	67	6879	15,03	0,006	2,60E-04	0,0455

**HWAY**			34	-05								
**KEGG_PATHWAY**	hsa05202:Transcriptional misregulation in cancer	8	13,79	4,16E-05	CDKN1A, IL6, CDKN1B, TP53, CXCL8, MDM2, NFKB1, MYC	41	167	6879	8,04	0,006	2,71E-04	0,0496
**KEGG_PATHWAY**	hsa04919:Thyroid hormone signaling pathway	7	12,07	4,62E-05	CCND1, KRAS, TP53, PIK3CA, MDM2, MTOR, MYC	41	115	6879	10,21	0,007	2,89E-04	0,0552
**KEGG_PATHWAY**	hsa04110:Cell cycle	7	12,07	7,06E-05	CDKN1A, CCND1, CDKN1B, PLK1, TP53, MDM2, MYC	41	124	6879	9,47	0,011	4,24E-04	0,0843
**KEGG_PATHWAY**	hsa05162:Measles	7	12,07	1,04E-04	IL6, TNFSF10, CCND1, CDKN1B, TP53, PIK3CA, NFKB1	41	133	6879	8,83	0,016	6,02E-04	0,1245
**KEGG_PATHWAY**	hsa05160:Hepatitis C	7	12,07	1,04E-04	EGFR, CDKN1A, KRAS, TP53, CXCL8, PIK3CA, NFKB1	41	133	6879	8,83	0,016	6,02E-04	0,1245
**KEGG_PATHWAY**	hsa05203:Viral carcinogenesis	8	13,79	1,52E-04	CDKN1A, CCND1, CDKN1B, KRAS, TP53, PIK3CA, MDM2, NFKB1	41	205	6879	6,55	0,023	8,45E-04	0,1816
**KEGG_PATHWAY**	hsa04210:Apoptosis	5	8,62	4,29E-04	TNFSF10, BCL2, TP53, PIK3CA, NFKB1	41	62	6879	13,53	0,062	0,0023	0,5110

**KEGG_PATHWAY**	hsa04010:MAPK signaling pathway	8	13,79	5,52E-04	EGFR, FGFR1, KRAS, DUSP1, TP53, NFKB1, MAP2K7, MYC	41	253	6879	5,31	0,079	0,0029	0,6568
**KEGG_PATHWAY**	hsa05166:HTLV-I infection	8	13,79	5,65E-04	CDKN1A, IL6, CCND1, KRAS, TP53, PIK3CA, NFKB1, MYC	41	254	6879	5,28	0,081	0,0028	0,6725
**KEGG_PATHWAY**	hsa05216:Thyroid cancer	4	6,90	5,99E-04	CCND1, KRAS, TP53, MYC	41	29	6879	23,14	0,086	0,0029	0,7134
**KEGG_PATHWAY**	hsa04722:Neurotrophin signaling pathway	6	10,34	6,00E-04	KRAS, BCL2, TP53, PIK3CA, NFKB1, MAP2K7	41	120	6879	8,39	0,086	0,0028	0,7142
**KEGG_PATHWAY**	hsa05231:Choline metabolism in cancer	5	8,62	0,0027	EGFR, KRAS, PIK3CA, RPS6KB1, MTOR	41	101	6879	8,31	0,330	0,0121	3,1389
**KEGG_PATHWAY**	hsa05164:Influenza A	6	10,34	0,0031	IL6, TNFSF10, CXCL8, PIK3CA, NFKB1, MAP2K7	41	174	6879	5,79	0,376	0,0138	3,6855
**KEGG_PATHWAY**	hsa04620:Toll-like receptor signaling pathway	5	8,62	0,0032	IL6, CXCL8, PIK3CA, NFKB1, MAP2K7	41	106	6879	7,91	0,380	0,0135	3,7299
**KEGG_PAT**	hsa04668:TNF signaling pathway	5	8,62	0,0033	IL6, PTGS2, PIK3CA, NFKB1, MAP2K7	41	107	6879	7,84	0,390	0,0136	3,8563

**HWAY**												
**KEGG_PATHWAY**	hsa04931:Insulin resistance	5	8,62	0,0034	IL6, PIK3CA, NFKB1, RPS6KB1, MTOR	41	108	6879	7,77	0,400	0,0137	3,9856
**KEGG_PATHWAY**	hsa04071:Sphingolipid signaling pathway	5	8,62	0,0050	KRAS, BCL2, TP53, PIK3CA, NFKB1	41	120	6879	6,99	0,525	0,0194	5,7621
**KEGG_PATHWAY**	hsa04015:Rap1 signaling pathway	6	10,34	0,0070	EGFR, FGFR1, KRAS, PIK3CA, THBS1, EPHA2	41	210	6879	4,79	0,650	0,0265	8,0136
**KEGG_PATHWAY**	hsa04917:Prolactin signaling pathway	4	6,90	0,0079	CCND1, KRAS, PIK3CA, NFKB1	41	71	6879	9,45	0,697	0,0294	9,0601
**KEGG_PATHWAY**	hsa04014:Ras signaling pathway	6	10,34	0,0094	EGFR, FGFR1, KRAS, PIK3CA, NFKB1, EPHA2	41	226	6879	4,45	0,759	0,0341	10,7031
**KEGG_PATHWAY**	hsa04921:Oxytocin signaling pathway	5	8,62	0,0108	EGFR, CDKN1A, CCND1, KRAS, PTGS2	41	150	6879	5,59	0,804	0,0380	12,1540
**KEGG_PATHWAY**	hsa04064:NF-kappa B signaling pathway	4	6,90	0,0138	PTGS2, BCL2, CXCL8, NFKB1	41	87	6879	7,71	0,875	0,0473	15,2713

**KEGG_PATHWAY**	hsa04912:GnRH signaling pathway	4	6,90	0,0155	EGFR, KRAS, PTK2B, MAP2K7	41	91	6879	7,37	0,905	0,0520	17,0636
**KEGG_PATHWAY**	hsa04660:T cell receptor signaling pathway	4	6,90	0,0200	KRAS, PIK3CA, NFKB1, MAP2K7	41	100	6879	6,71	0,951	0,0650	21,4007
**KEGG_PATHWAY**	hsa05142:Chagas disease (American trypanosomiasis)	4	6,90	0,0221	IL6, CXCL8, PIK3CA, NFKB1	41	104	6879	6,45	0,965	0,0704	23,4488
**KEGG_PATHWAY**	hsa04062:Chemokine signaling pathway	5	8,62	0,0222	KRAS, PTK2B, CXCL8, PIK3CA, NFKB1	41	186	6879	4,51	0,965	0,0690	23,4710
**KEGG_PATHWAY**	hsa05146:Amoebiasis	4	6,90	0,0233	IL6, CXCL8, PIK3CA, NFKB1	41	106	6879	6,33	0,971	0,0709	24,4973
**KEGG_PATHWAY**	hsa04510:Focal adhesion	5	8,62	0,0308	EGFR, CCND1, BCL2, PIK3CA, THBS1	41	206	6879	4,07	0,991	0,0913	31,1580
**KEGG_PATHWAY**	hsa05144:Malaria	3	5,17	0,0326	IL6, CXCL8, THBS1	41	49	6879	10,27	0,993	0,0948	32,7237
**KEGG_PAT**	hsa04650:Natural killer cell mediated cytotoxicity	4	6,90	0,0334	TNFSF10, KRAS, PTK2B, PIK3CA	41	122	6879	5,50	0,994	0,0951	33,3492

**HWAY**												
**KEGG_PATHWAY**	hsa04152:AMPK signaling pathway	4	6,90	0,0341	CCND1, PIK3CA, RPS6KB1, MTOR	41	123	6879	5,46	0,995	0,0952	33,9232
**KEGG_PATHWAY**	hsa05134:Legionellosis	3	5,17	0,0390	IL6, CXCL8, NFKB1	41	54	6879	9,32	0,997	0,1065	37,8242
**KEGG_PATHWAY**	hsa04380:Osteoclast differentiation	4	6,90	0,0400	CYBB, PIK3CA, NFKB1, MAP2K7	41	131	6879	5,12	0,998	0,1071	38,5649
**KEGG_PATHWAY**	hsa04621:NOD-like receptor signaling pathway	3	5,17	0,0417	IL6, CXCL8, NFKB1	41	56	6879	8,99	0,998	0,1096	39,8551
**KEGG_PATHWAY**	hsa04150:mTOR signaling pathway	3	5,17	0,0444	PIK3CA, RPS6KB1, MTOR	41	58	6879	8,68	0,998	0,1146	41,8740
**KEGG_PATHWAY**	hsa04910:Insulin signaling pathway	4	6,90	0,0455	KRAS, PIK3CA, RPS6KB1, MTOR	41	138	6879	4,86	0,999	0,1154	42,6648
**KEGG_PATHWAY**	hsa04550:Signaling pathways regulating pluripotency of stem cells	4	6,90	0,0472	FGFR1, KRAS, PIK3CA, MYC	41	140	6879	4,79	0,999	0,1175	43,8365

**KEGG_PATHWAY**	hsa04370:VEGF signaling pathway	3	5,17	0,0486	KRAS, PTGS2, PIK3CA	41	61	6879	8,25	0,999	0,1191	44,8705
**KEGG_PATHWAY**	hsa04630:Jak-STAT signaling pathway	4	6,90	0,0514	IL6, CCND1, PIK3CA, MYC	41	145	6879	4,63	0,999	0,1236	46,7570
**KEGG_PATHWAY**	hsa05131:Shigellosis	3	5,17	0,0530	CD44, CXCL8, NFKB1	41	64	6879	7,86	0,999	0,1254	47,8177
**KEGG_PATHWAY**	hsa04932:Non-alcoholic fatty liver disease (NAFLD)	4	6,90	0,0568	IL6, CXCL8, PIK3CA, NFKB1	41	151	6879	4,44	0,999	0,1318	50,2290
**KEGG_PATHWAY**	hsa05120:Epithelial cell signaling in Helicobacter pylori infection	3	5,17	0,0575	EGFR, CXCL8, NFKB1	41	67	6879	7,51	0,999	0,1315	50,7045
**KEGG_PATHWAY**	hsa04664:Fc epsilon RI signaling pathway	3	5,17	0,0590	KRAS, PIK3CA, MAP2K7	41	68	6879	7,40	0,999	0,1329	51,6515
**KEGG_PATHWAY**	hsa04662:B cell receptor signaling pathway	3	5,17	0,0606	KRAS, PIK3CA, NFKB1	41	69	6879	7,29	0,999	0,1343	52,5904
**KEGG_PAT**	hsa05133:Pertussis	3	5,17	0,0701	IL6, CXCL8, NFKB1	41	75	6879	6,71	0,999	0,1523	58,0362

**HWAY**												
**KEGG_PATHWAY**	hsa05204:Chemical carcinogenesis	3	5,17	0,0784	PTGS2, SULT1A1, GSTO1	41	80	6879	6,29	0,999	0,1671	62,3025
**KEGG_PATHWAY**	hsa05132:Salmonella infection	3	5,17	0,0836	IL6, CXCL8, NFKB1	41	83	6879	6,06	0,999	0,1751	64,7334
**KEGG_PATHWAY**	hsa04350:TGF-beta signaling pathway	3	5,17	0,0853	RPS6KB1, THBS1, MYC	41	84	6879	5,99	0,999	0,1762	65,5215
**KEGG_PATHWAY**	hsa04914:Progesterone-mediated oocyte maturation	3	5,17	0,0906	KRAS, PLK1, PIK3CA	41	87	6879	5,79	0,999	0,1841	67,8176

C. Signaling pathways in which the potential targets of miR-9-5p are involved (identified by DAVID)

Table 1: targets identified by BIOCARTA (DAVID tool)

Table 2: targets identified by KEGG (DAVID tool)

Category	Term	Count	%	PValue	Genes	List Total	Pop Hits	Pop Total	Fold Enrichment	Bonferroni	Benjamini	FDR
**BIOCARTA**	h_her2Pathway:Role of ERBB2 in Signal Transduction and Oncology	7	8,24	1,12E-05	PIK3CG, EGFR, IL6, EP300, RAF1, PIK3CA, PIK3R1	41	23	1625	12,063	0,002	0,0019	0,0136
**BIOCARTA**	h_ctcfPathway:CTCF: First Multivalent Nuclear Factor	7	8,24	1,90E-05	PIK3CG, CDKN1B, PIK3CA, MDM2, PTEN, PIK3R1, TGFB2	41	25	1625	11,098	0,003	0,0016	0,0231
**BIOCARTA**	h_rasPathway:Ras Signaling Pathway	6	7,06	1,70E-04	PIK3CG, RAF1, PIK3CA, ELK1, NFKB1, PIK3R1	41	23	1625	10,339	0,028	0,0094	0,2065
**BIOCARTA**	h_metPathway:Signaling of Hepatocyte Growth Factor Receptor	7	8,24	1,71E-04	PIK3CG, MET, RAF1, PIK3CA, ELK1, PTEN, PIK3R1	41	36	1625	7,707	0,028	0,0071	0,2075
**BIOCARTA**	h_fcer1Pathway:Fc Epsilon Receptor I Signaling in Mast Cells	7	8,24	2,71E-04	PIK3CG, PLA2G4A, RAF1, PIK3CA, ELK1, MAP2K7, PIK3R1	41	39	1625	7,114	0,044	0,0090	0,3288
**BIOCARTA**	h_raccycdPathway:Influence of Ras and Rho proteins on G1 to S Transition	6	7,06	3,80E-04	CCND1, CDKN1B, RAF1, PIK3CA, NFKB1, PIK3R1	41	27	1625	8,808	0,061	0,0104	0,4602
**BIOCARTA**	h_il7Pathway:IL-7 Signal Transduction	5	5,88	5,95E-04	PIK3CG, EP300, BCL2, PIK3CA, PIK3R1	41	17	1625	11,657	0,094	0,0140	0,7211

**BIOCARTA**	h_gcrpathway:Corticosteroids and cardioprotection	5	5,88	9,36E-04	PIK3CG, PIK3CA, NFKB1, NR3C1, PIK3R1	41	19	1625	10,430	0,144	0,0192	1,1310
**BIOCARTA**	h_ngfPathway:Nerve growth factor pathway (NGF)	5	5,88	0,0011	PIK3CG, RAF1, PIK3CA, ELK1, PIK3R1	41	20	1625	9,909	0,174	0,0210	1,3871
**BIOCARTA**	h_igf1Pathway:IGF-1 Signaling Pathway	5	5,88	0,0014	PIK3CG, RAF1, PIK3CA, ELK1, PIK3R1	41	21	1625	9,437	0,207	0,0229	1,6808
**BIOCARTA**	h_insulinPathway:Insulin Signaling Pathway	5	5,88	0,0014	PIK3CG, RAF1, PIK3CA, ELK1, PIK3R1	41	21	1625	9,437	0,207	0,0229	1,6808
**BIOCARTA**	h_egfPathway:EGF Signaling Pathway	5	5,88	0,0037	EGFR, RAF1, PIK3CA, ELK1, PIK3R1	41	27	1625	7,340	0,457	0,0540	4,3731
**BIOCARTA**	h_tcrPathway:T Cell Receptor Signaling Pathway	6	7,06	0,0038	PIK3CG, RAF1, PIK3CA, ELK1, NFKB1, PIK3R1	41	44	1625	5,405	0,464	0,0507	4,4634
**BIOCARTA**	h_pdgfPathway:PDGF Signaling Pathway	5	5,88	0,0042	PIK3CG, RAF1, PIK3CA, ELK1, PIK3R1	41	28	1625	7,078	0,504	0,0524	4,9970
**BIOCARTA**	h_bcellsurvivalPathway:B Cell Survival Pathway	4	4,71	0,0051	PIK3CG, CASP3, PIK3CA, PIK3R1	41	15	1625	10,569	0,574	0,0591	6,0508
**BIOCARTA**	h_vegfPathway:VEGF, Hypoxia, and Angiogenesis	5	5,88	0,0061	PIK3CG, HIF1A, VEGFA, PIK3CA, PIK3R1	41	31	1625	6,393	0,640	0,0658	7,2028
**BIOCARTA**	h_hcmvPathway:Human Cytomegalovirus and Map Kinase Pathways	4	4,71	0,0074	PIK3CG, PIK3CA, NFKB1, PIK3R1	41	17	1625	9,326	0,709	0,0742	8,6295

**BIOCARTA**	h_deathPathway:Induction of apoptosis through DR3 and DR4/5 Death Receptors	5	5,88	0,0077	CASP3, TNFSF10, BCL2, LMNA, NFKB1	41	33	1625	6,005	0,723	0,0727	8,9622
**BIOCARTA**	h_ptenPathway:PTEN dependent cell cycle arrest and apoptosis	4	4,71	0,0087	CDKN1B, PIK3CA, PTEN, PIK3R1	41	18	1625	8,808	0,767	0,0777	10,1055
**BIOCARTA**	h_arfPathway:Tumor Suppressor Arf Inhibits Ribosomal Biogenesis	4	4,71	0,0087	PIK3CG, PIK3CA, MDM2, PIK3R1	41	18	1625	8,808	0,767	0,0777	10,1055
**BIOCARTA**	h_igf1mtorPathway:Skeletal muscle hypertrophy is regulated via AKT/mTOR pathway	4	4,71	0,0118	EIF4E, PIK3CA, PTEN, PIK3R1	41	20	1625	7,927	0,860	0,0984	13,4166
**BIOCARTA**	h_il2rbPathway:IL-2 Receptor Beta Chain in T cell Activation	5	5,88	0,0139	PIK3CG, BCL2, RAF1, PIK3CA, PIK3R1	41	39	1625	5,081	0,902	0,1098	15,6504
**BIOCARTA**	h_hivnefPathway:HIV-I Nef: negative effector of Fas and TNF	6	7,06	0,0151	CASP3, BCL2, LMNA, MDM2, NFKB1, MAP2K7	41	61	1625	3,898	0,920	0,1133	16,8818
**BIOCARTA**	h_aktPathway:AKT Signaling Pathway	4	4,71	0,0154	PIK3CG, PIK3CA, NFKB1, PIK3R1	41	22	1625	7,206	0,924	0,1104	17,1745
**BIOCARTA**	h_gleevecPathway:Inhibition of Cellular Proliferation by Gleevec	4	4,71	0,0174	PIK3CG, RAF1, PIK3CA, PIK3R1	41	23	1625	6,893	0,946	0,1190	19,2055
**BIOCARTA**	h_plcPathway:Phospholipase C Signaling Pathway	3	3,53	0,0191	PIK3CG, PIK3CA, PIK3R1	41	9	1625	13,211	0,959	0,1247	20,8616
**BIOCARTA**	h_tpoPathway:TPO Signaling Pathway	4	4,71	0,0195	PIK3CG, RAF1, PIK3CA, PIK3R1	41	24	1625	6,606	0,962	0,1229	21,3278

**BIOCARTA**	h_ecmPathway:Erk and PI-3 Kinase Are Necessary for Collagen Binding in Corneal Epithelia	4	4,71	0,0195	PIK3CG, RAF1, PIK3CA, PIK3R1	41	24	1625	6,606	0,962	0,1229	21,3278
**BIOCARTA**	h_cxcr4Pathway:CXCR4 Signaling Pathway	4	4,71	0,0195	RAF1, PIK3CA, NFKB1, PIK3R1	41	24	1625	6,606	0,962	0,1229	21,3278
**BIOCARTA**	h_nthiPathway:NFkB activation by Nontypeable Hemophilus influenza	4	4,71	0,0195	EP300, DUSP1, NFKB1, NR3C1	41	24	1625	6,606	0,962	0,1229	21,3278
**BIOCARTA**	h_eif4Pathway:Regulation of eIF4e and p70 S6 Kinase	4	4,71	0,0195	EIF4E, PIK3CA, PTEN, PIK3R1	41	24	1625	6,606	0,962	0,1229	21,3278
**BIOCARTA**	h_g2Pathway:Cell Cycle: G2/M Checkpoint	4	4,71	0,0218	EP300, PLK1, MDM2, ATR	41	25	1625	6,341	0,974	0,1315	23,5331
**BIOCARTA**	h_badPathway:Regulation of BAD phosphorylation	4	4,71	0,0243	PIK3CG, BCL2, PIK3CA, PIK3R1	41	26	1625	6,098	0,983	0,1402	25,8126
**BIOCARTA**	h_tffPathway:Trefoil Factors Initiate Mucosal Healing	4	4,71	0,0243	PIK3CG, EGFR, PIK3CA, PIK3R1	41	26	1625	6,098	0,983	0,1402	25,8126
**BIOCARTA**	h_mtorPathway:mTOR Signaling Pathway	4	4,71	0,0243	EIF4E, PIK3CA, PTEN, PIK3R1	41	26	1625	6,098	0,983	0,1402	25,8126
**BIOCARTA**	H_gsk3Pathway:Inactivation of Gsk3 by AKT causes accumulation of b-catenin in Alveolar Macrophages	4	4,71	0,0268	CCND1, PIK3CA, NFKB1, PIK3R1	41	27	1625	5,872	0,989	0,1490	28,1568
**BIOCARTA**	h_ghPathway:Growth Hormone Signaling Pathway	4	4,71	0,0268	PIK3CG, RAF1, PIK3CA, PIK3R1	41	27	1625	5,872	0,989	0,1490	28,1568

**BIOCARTA**	h_keratinocytePathway:Keratinocyte Differentiation	5	5,88	0,0281	EGFR, BCL2, RAF1, NFKB1, MAP2K7	41	48	1625	4,129	0,991	0,1503	29,2382
**BIOCARTA**	h_g1Pathway:Cell Cycle: G1/S Check Point	4	4,71	0,0354	CCND1, CDKN1B, ATR, TGFB2	41	30	1625	5,285	0,997	0,1809	35,4801
**BIOCARTA**	h_PparaPathway:Mechanism of Gene Regulation by Peroxisome Proliferators via PPARa(alpha)	5	5,88	0,0364	PIK3CG, EP300, DUSP1, PIK3CA, PIK3R1	41	52	1625	3,811	0,997	0,1800	36,2582
**BIOCARTA**	h_longevityPathway:The IGF-1 Receptor and Longevity	3	3,53	0,0507	PIK3CG, PIK3CA, PIK3R1	41	15	1625	7,927	0,999	0,2365	46,8492
**BIOCARTA**	h_achPathway:Role of nicotinic acetylcholine receptors in the regulation of apoptosis	3	3,53	0,0570	PIK3CG, PIK3CA, PIK3R1	41	16	1625	7,431	0,999	0,2558	51,0150
**BIOCARTA**	h_p53Pathway:p53 Signaling Pathway	3	3,53	0,0637	CCND1, BCL2, MDM2	41	17	1625	6,994	0,999	0,2747	55,0333
**BIOCARTA**	h_hifPathway:Hypoxia-Inducible Factor in the Cardiovascular System	3	3,53	0,0637	HIF1A, EP300, VEGFA	41	17	1625	6,994	0,999	0,2747	55,0333
**BIOCARTA**	h_erk5Pathway:Role of Erk5 in Neuronal Survival	3	3,53	0,0705	PIK3CG, PIK3CA, PIK3R1	41	18	1625	6,606	0,999	0,2931	58,8802
**BIOCARTA**	h_il6Pathway:IL 6 signaling pathway	3	3,53	0,0925	IL6, RAF1, ELK1	41	21	1625	5,662	0,999	0,3607	69,2362

Category	Term	Count	%	PValue	Genes	List Total	Pop Hits	Pop Total	Fold Enrichment	Bonferroni	Benjamini	FDR
**KEGG_PATHWAY**	hsa05200:Pathways in cancer	28	32,94	1,72E-19	FGFR1, FGFR3, PTGS2, NFKB1, PTEN, SHH, TGFB2, CASP3, BCL2, PIK3CA, PIK3R1, PIK3CG, EGFR, AR, IL6, PIK3CB, MSH2, PIK3CD, MET, RAF1, DAPK1, CCND1, CDKN1B, HIF1A, EP300, VEGFA, MDM2, PTCH1	57	393	6879	8,60	2,81E-17	2,81E-17	2,09E-16
**KEGG_PATHWAY**	hsa05205:Proteoglycans in cancer	21	24,71	2,16E-17	EGFR, PIK3CG, FGFR1, PIK3CB, MET, PIK3CD, RAF1, ELK1, IGF2, TGFB2, CASP3, CCND1, SDC1, HIF1A, CD44, VEGFA, MDM2, PIK3CA, PTCH1, THBS1, PIK3R1	57	200	6879	12,67	3,52E-15	1,76E-15	2,61E-14
**KEGG_PATHWAY**	hsa05215:Prostate cancer	16	18,82	1,21E-16	PIK3CG, EGFR, FGFR1, AR, PIK3CB, PIK3CD, RAF1, NFKB1, PTEN, CCND1, EP300, CDKN1B, BCL2, MDM2, PIK3CA, PIK3R1	57	88	6879	21,94	1,81E-14	6,00E-15	1,33E-13
**KEGG_PATHWAY**	hsa04151:PI3K-Akt signaling pathway	22	25,88	7,53E-14	EGFR, PIK3CG, FGFR1, IL6, FGFR3, EFNA1, PIK3CB, PIK3CD, MET, RAF1, NFKB1, PTEN, EPHA2, CCND1, EIF4E, CDKN1B, BCL2, VEGFA, MDM2, PIK3CA, THBS1, PIK3R1	57	345	6879	7,70	1,23E-11	3,07E-12	9,12E-11
**KEGG_PATHWAY**	hsa04068:FoxO signaling pathway	16	18,82	8,24E-14	EGFR, PIK3CG, IL6, PIK3CB, PIK3CD, RAF1, PTEN, TGFB2, CCND1, TNFSF10, EP300, CDKN1B, PLK1, MDM2, PIK3CA, PIK3R1	57	134	6879	14,41	1,34E-11	2,69E-12	9,98E-11

**KEGG_PATHWAY**	hsa05161:Hepatitis B	16	18,82	2,69E-13	PIK3CG, IL6, PIK3CB, PIK3CD, RAF1, ELK1, NFKB1, PTEN, TGFB2, CASP3, CCND1, EP300, CDKN1B, BCL2, PIK3CA, PIK3R1	57	145	6879	13,32	4,39E-11	7,32E-12	3,26E-10
**KEGG_PATHWAY**	hsa04066:HIF-1 signaling pathway	14	16,47	3,83E-13	EGFR, PIK3CG, IL6, EIF4E, CDKN1B, HIF1A, EP300, PIK3CB, BCL2, PIK3CD, VEGFA, PIK3CA, NFKB1, PIK3R1	57	96	6879	17,60	6,24E-11	8,91E-12	4,63E-10
**KEGG_PATHWAY**	hsa05230:Central carbon metabolism in cancer	12	14,12	1,98E-12	PIK3CG, EGFR, FGFR1, HIF1A, FGFR3, PIK3CB, PIK3CD, MET, RAF1, PIK3CA, PTEN, PIK3R1	57	64	6879	22,63	3,23E-10	4,04E-11	2,40E-09
**KEGG_PATHWAY**	hsa05206:MicroRNAs in cancer	19	22,35	4,13E-12	EGFR, FGFR3, PTGS2, MET, RAF1, NFKB1, PTEN, TGFB2, CASP3, CCND1, EP300, CDKN1B, CD44, BCL2, VEGFA, DNMT1, MDM2, STMN1, THBS1	57	286	6879	8,02	6,73E-10	7,48E-11	5,00E-09
**KEGG_PATHWAY**	hsa05218:Melanoma	12	14,12	6,54E-12	PIK3CG, EGFR, FGFR1, CCND1, PIK3CB, PIK3CD, MET, RAF1, PIK3CA, MDM2, PTEN, PIK3R1	57	71	6879	20,40	1,07E-09	1,07E-10	7,92E-09
**KEGG_PATHWAY**	hsa05210:Colorectal cancer	11	12,94	4,29E-11	PIK3CG, CASP3, CCND1, PIK3CB, MSH2, BCL2, PIK3CD, RAF1, PIK3CA, PIK3R1, TGFB2	57	62	6879	21,41	6,99E-09	6,35E-10	5,19E-08

**KEGG_PATHWAY**	hsa05212:Pancreatic cancer	11	12,94	7,01E-11	PIK3CG, EGFR, CCND1, PIK3CB, PIK3CD, VEGFA, RAF1, PIK3CA, NFKB1, PIK3R1, TGFB2	57	65	6879	20,42	1,14E-08	9,52E-10	8,49E-08
**KEGG_PATHWAY**	hsa05211:Renal cell carcinoma	11	12,94	8,21E-11	PIK3CG, HIF1A, EP300, PIK3CB, PIK3CD, VEGFA, MET, RAF1, PIK3CA, PIK3R1, TGFB2	57	66	6879	20,11	1,34E-08	1,03E-09	9,94E-08
**KEGG_PATHWAY**	hsa04014:Ras signaling pathway	16	18,82	1,73E-10	EGFR, PIK3CG, FGFR1, FGFR3, EFNA1, PIK3CB, PIK3CD, MET, RAF1, ELK1, NFKB1, EPHA2, PLA2G4A, VEGFA, PIK3CA, PIK3R1	57	226	6879	8,54	2,83E-08	2,02E-09	2,10E-07
**KEGG_PATHWAY**	hsa05220:Chronic myeloid leukemia	11	12,94	2,01E-10	PIK3CG, CCND1, CDKN1B, PIK3CB, PIK3CD, RAF1, PIK3CA, MDM2, NFKB1, PIK3R1, TGFB2	57	72	6879	18,44	3,28E-08	2,19E-09	2,44E-07
**KEGG_PATHWAY**	hsa05213:Endometrial cancer	10	11,76	2,26E-10	PIK3CG, EGFR, CCND1, PIK3CB, PIK3CD, RAF1, PIK3CA, ELK1, PTEN, PIK3R1	57	52	6879	23,21	3,68E-08	2,30E-09	2,74E-07
**KEGG_PATHWAY**	hsa05222:Small cell lung cancer	11	12,94	1,08E-09	PIK3CG, CCND1, CDKN1B, PTGS2, PIK3CB, BCL2, PIK3CD, PIK3CA, NFKB1, PTEN, PIK3R1	57	85	6879	15,62	1,77E-07	1,04E-08	1,31E-06

**KEGG_PATHWAY**	hsa05214:Glioma	10	11,76	1,81E-09	PIK3CG, EGFR, CCND1, PIK3CB, PIK3CD, RAF1, PIK3CA, MDM2, PTEN, PIK3R1	57	65	6879	18,57	2,95E-07	1,64E-08	2,19E-06
**KEGG_PATHWAY**	hsa04510:Focal adhesion	14	16,47	6,49E-09	EGFR, PIK3CG, PIK3CB, PIK3CD, MET, ELK1, RAF1, PTEN, CCND1, BCL2, VEGFA, PIK3CA, THBS1, PIK3R1	57	206	6879	8,20	1,06E-06	5,57E-08	7,86E-06
**KEGG_PATHWAY**	hsa04015:Rap1 signaling pathway	14	16,47	8,20E-09	PIK3CG, EGFR, FGFR1, FGFR3, EFNA1, PIK3CB, MET, PIK3CD, RAF1, EPHA2, VEGFA, PIK3CA, THBS1, PIK3R1	57	210	6879	8,05	1,34E-06	6,68E-08	9,93E-06
**KEGG_PATHWAY**	hsa04370:VEGF signaling pathway	9	10,59	2,43E-08	PIK3CG, PLA2G4A, PTGS2, PIK3CB, PIK3CD, VEGFA, RAF1, PIK3CA, PIK3R1	57	61	6879	17,81	3,96E-06	1,88E-07	2,94E-05
**KEGG_PATHWAY**	hsa04012:ErbB signaling pathway	10	11,76	2,54E-08	PIK3CG, EGFR, CDKN1B, PIK3CB, PIK3CD, RAF1, PIK3CA, ELK1, MAP2K7, PIK3R1	57	87	6879	13,87	4,13E-06	1,88E-07	3,07E-05
**KEGG_PATHWAY**	hsa04210:Apoptosis	9	10,59	2,77E-08	PIK3CG, CASP3, TNFSF10, PIK3CB, BCL2, PIK3CD, PIK3CA, NFKB1, PIK3R1	57	62	6879	17,52	4,51E-06	1,96E-07	3,35E-05

**KEGG_PATHWAY**	hsa05219:Bladder cancer	8	9,41	2,92E-08	EGFR, CCND1, FGFR3, VEGFA, RAF1, MDM2, THBS1, DAPK1	57	41	6879	23,55	4,76E-06	1,99E-07	3,54E-05
**KEGG_PATHWAY**	hsa05169:Epstein-Barr virus infection	11	12,94	3,80E-08	PIK3CG, CDKN1B, CD44, PIK3CB, BCL2, PIK3CD, PIK3CA, MDM2, NFKB1, MAP2K7, PIK3R1	57	122	6879	10,88	6,19E-06	2,48E-07	4,60E-05
**KEGG_PATHWAY**	hsa04668:TNF signaling pathway	10	11,76	1,57E-07	PIK3CG, CASP3, IL6, PTGS2, PIK3CB, PIK3CD, PIK3CA, NFKB1, MAP2K7, PIK3R1	57	107	6879	11,28	2,55E-05	9,81E-07	1,90E-04
**KEGG_PATHWAY**	hsa05221:Acute myeloid leukemia	8	9,41	2,74E-07	PIK3CG, CCND1, PIK3CB, PIK3CD, RAF1, PIK3CA, NFKB1, PIK3R1	57	56	6879	17,24	4,47E-05	1,66E-06	3,32E-04
**KEGG_PATHWAY**	hsa05223:Non-small cell lung cancer	8	9,41	2,74E-07	PIK3CG, EGFR, CCND1, PIK3CB, PIK3CD, RAF1, PIK3CA, PIK3R1	57	56	6879	17,24	4,47E-05	1,66E-06	3,32E-04
**KEGG_PATHWAY**	hsa04919:Thyroid hormone signaling pathway	10	11,76	2,92E-07	PIK3CG, CCND1, HIF1A, EP300, PIK3CB, PIK3CD, RAF1, PIK3CA, MDM2, PIK3R1	57	115	6879	10,49	4,76E-05	1,70E-06	3,54E-04
**KEGG_PATHWAY**	hsa05162:Measles	10	11,	1,01E-06	PIK3CG, IL6, TNFSF10, CCND1, CDKN1B, PIK3CB, PIK3CD, PIK3CA, NFKB1, PIK3R1	57	133	6879	9,07	1,65E-04	5,68E-06	0,0012

			76									
**KEGG_PATHWAY**	hsa04664:Fc epsilon RI signaling pathway	8	9,41	1,06E-06	PIK3CG, PLA2G4A, PIK3CB, PIK3CD, RAF1, PIK3CA, MAP2K7, PIK3R1	57	68	6879	14,20	1,73E-04	5,78E-06	0,0013
**KEGG_PATHWAY**	hsa05164:Influenza A	11	12,94	1,08E-06	PIK3CG, IL6, TNFSF10, EP300, PIK3CB, PIK3CD, RAF1, PIK3CA, NFKB1, MAP2K7, PIK3R1	57	174	6879	7,63	1,76E-04	5,67E-06	0,0013
**KEGG_PATHWAY**	hsa05231:Choline metabolism in cancer	9	10,59	1,30E-06	PIK3CG, EGFR, PLA2G4A, HIF1A, PIK3CB, PIK3CD, RAF1, PIK3CA, PIK3R1	57	101	6879	10,75	2,11E-04	6,61E-06	0,0016
**KEGG_PATHWAY**	hsa04917:Prolactin signaling pathway	8	9,41	1,43E-06	PIK3CG, CCND1, PIK3CB, PIK3CD, RAF1, PIK3CA, NFKB1, PIK3R1	57	71	6879	13,60	2,33E-04	7,08E-06	0,0017
**KEGG_PATHWAY**	hsa05146:Amoebiasis	9	10,59	1,88E-06	PIK3CG, CASP3, IL6, PIK3CB, PIK3CD, PIK3CA, NFKB1, PIK3R1, TGFB2	57	106	6879	10,25	3,06E-04	8,99E-06	0,0023
**KEGG_PATHWAY**	hsa04010:MAPK signaling pathway	12	14,12	4,59E-06	EGFR, FGFR1, PLA2G4A, CASP3, FGFR3, DUSP1, RAF1, ELK1, NFKB1, STMN1, MAP2K7, TGFB2	57	253	6879	5,72	7,49E-04	2,14E-05	0,0056

**KEGG_PATHWAY**	hsa05203:Viral carcinogenesis	11	12,94	4,77E-06	PIK3CG, CASP3, CCND1, CDKN1B, EP300, PIK3CB, PIK3CD, PIK3CA, MDM2, NFKB1, PIK3R1	57	205	6879	6,48	7,78E-04	2,16E-05	0,0058
**KEGG_PATHWAY**	hsa05166:HTLV-I infection	12	14,12	4,77E-06	PIK3CG, IL6, CCND1, EP300, PIK3CB, PIK3CD, PIK3CA, ELK1, NFKB1, ATR, PIK3R1, TGFB2	57	254	6879	5,70	7,78E-04	2,10E-05	0,0058
**KEGG_PATHWAY**	hsa04722:Neurotrophin signaling pathway	9	10,59	4,79E-06	PIK3CG, PIK3CB, BCL2, PIK3CD, RAF1, PIK3CA, NFKB1, MAP2K7, PIK3R1	57	120	6879	9,05	7,80E-04	2,05E-05	0,0058
**KEGG_PATHWAY**	hsa04071:Sphingolipid signaling pathway	9	10,59	4,79E-06	PIK3CG, PIK3CB, BCL2, PIK3CD, RAF1, PIK3CA, NFKB1, PTEN, PIK3R1	57	120	6879	9,05	7,80E-04	2,05E-05	0,0058
**KEGG_PATHWAY**	hsa04150:mTOR signaling pathway	7	8,24	6,46E-06	PIK3CG, EIF4E, PIK3CB, PIK3CD, PIK3CA, PTEN, PIK3R1	57	58	6879	14,57	0,001	2,70E-05	0,0078
**KEGG_PATHWAY**	hsa04380:Osteoclast differentiation	9	10,59	9,20E-06	PIK3CG, CYBB, PIK3CB, PIK3CD, PIK3CA, NFKB1, MAP2K7, PIK3R1, TGFB2	57	131	6879	8,29	0,001	3,75E-05	0,0111

**KEGG_PATHWAY**	hsa04660:T cell receptor signaling pathway	8	9,41	1,44E-05	PIK3CG, PIK3CB, PIK3CD, RAF1, PIK3CA, NFKB1, MAP2K7, PIK3R1	57	100	6879	9,65	0,002	5,72E-05	0,0174
**KEGG_PATHWAY**	hsa04115:p53 signaling pathway	7	8,24	1,51E-05	CASP3, CCND1, RRM2, MDM2, ATR, THBS1, PTEN	57	67	6879	12,61	0,002	5,85E-05	0,0182
**KEGG_PATHWAY**	hsa04662:B cell receptor signaling pathway	7	8,24	1,79E-05	PIK3CG, PIK3CB, PIK3CD, RAF1, PIK3CA, NFKB1, PIK3R1	57	69	6879	12,24	0,003	6,78E-05	0,0216
**KEGG_PATHWAY**	hsa05142:Chagas disease (American trypanosomiasis)	8	9,41	1,86E-05	PIK3CG, IL6, PIK3CB, PIK3CD, PIK3CA, NFKB1, PIK3R1, TGFB2	57	104	6879	9,28	0,003	6,89E-05	0,0225
**KEGG_PATHWAY**	hsa04620:Toll-like receptor signaling pathway	8	9,41	2,11E-05	PIK3CG, IL6, PIK3CB, PIK3CD, PIK3CA, NFKB1, MAP2K7, PIK3R1	57	106	6879	9,11	0,003	7,64E-05	0,0255
**KEGG_PATHWAY**	hsa04931:Insulin resistance	8	9,41	2,38E-05	PIK3CG, IL6, PIK3CB, PIK3CD, PIK3CA, NFKB1, PTEN, PIK3R1	57	108	6879	8,94	0,004	8,44E-05	0,0288
**KEGG_PATHWAY**	hsa04810:Regulation of actin cytoskeleton	10	11,76	4,23E-05	PIK3CG, EGFR, FGFR1, FGFR3, PIK3CB, PIK3CD, RAF1, PIK3CA, TMSB4X, PIK3R1	57	210	6879	5,75	0,007	1,47E-04	0,0512

**KEGG_PATHWAY**	hsa04650:Natural killer cell mediated cytotoxicity	8	9,41	5,24E-05	PIK3CG, CASP3, TNFSF10, PIK3CB, PIK3CD, RAF1, PIK3CA, PIK3R1	57	122	6879	7,91	0,009	1,78E-04	0,0635
**KEGG_PATHWAY**	hsa04914:Progesterone-mediated oocyte maturation	7	8,24	6,73E-05	PIK3CG, PIK3CB, PLK1, PIK3CD, RAF1, PIK3CA, PIK3R1	57	87	6879	9,71	0,011	2,24E-04	0,0815
**KEGG_PATHWAY**	hsa04923:Regulation of lipolysis in adipocytes	6	7,06	8,30E-05	PIK3CG, PTGS2, PIK3CB, PIK3CD, PIK3CA, PIK3R1	57	56	6879	12,93	0,013	2,71E-04	0,1005
**KEGG_PATHWAY**	hsa05160:Hepatitis C	8	9,41	9,08E-05	PIK3CG, EGFR, PIK3CB, PIK3CD, RAF1, PIK3CA, NFKB1, PIK3R1	57	133	6879	7,26	0,015	2,90E-04	0,1100
**KEGG_PATHWAY**	hsa04910:Insulin signaling pathway	8	9,41	1,15E-04	PIK3CG, EIF4E, PIK3CB, PIK3CD, RAF1, PIK3CA, ELK1, PIK3R1	57	138	6879	7,00	0,019	3,59E-04	0,1388
**KEGG_PATHWAY**	hsa04550:Signaling pathways regulating pluripotency of stem cells	8	9,41	1,26E-04	PIK3CG, FGFR1, FGFR3, PIK3CB, PIK3CD, RAF1, PIK3CA, PIK3R1	57	140	6879	6,90	0,020	3,86E-04	0,1519
**KEGG_PATHWAY**	hsa04915:Estrogen signaling pathway	7	8,24	1,39E-04	PIK3CG, EGFR, PIK3CB, PIK3CD, RAF1, PIK3CA, PIK3R1	57	99	6879	8,53	0,022	4,18E-04	0,1677

**KEGG_PATHWAY**	hsa04630:Jak-STAT signaling pathway	8	9,41	1,56E-04	PIK3CG, IL6, CCND1, EP300, PIK3CB, PIK3CD, PIK3CA, PIK3R1	57	145	6879	6,66	0,025	4,63E-04	0,1892
**KEGG_PATHWAY**	hsa04024:cAMP signaling pathway	9	10,59	1,77E-04	PIK3CG, EP300, PIK3CB, PIK3CD, RAF1, PIK3CA, NFKB1, PTCH1, PIK3R1	57	198	6879	5,49	0,029	5,16E-04	0,2146
**KEGG_PATHWAY**	hsa04932:Non-alcoholic fatty liver disease (NAFLD)	8	9,41	2,01E-04	PIK3CG, CASP3, IL6, PIK3CB, PIK3CD, PIK3CA, NFKB1, PIK3R1	57	151	6879	6,39	0,032	5,75E-04	0,2434
**KEGG_PATHWAY**	hsa04960:Aldosterone-regulated sodium reabsorption	5	5,88	2,62E-04	PIK3CG, PIK3CB, PIK3CD, PIK3CA, PIK3R1	57	39	6879	15,47	0,042	7,37E-04	0,3171
**KEGG_PATHWAY**	hsa04973:Carbohydrate digestion and absorption	5	5,88	3,50E-04	PIK3CG, PIK3CB, PIK3CD, PIK3CA, PIK3R1	57	42	6879	14,37	0,056	9,68E-04	0,4236
**KEGG_PATHWAY**	hsa05100:Bacterial invasion of epithelial cells	6	7,06	4,00E-04	PIK3CG, PIK3CB, PIK3CD, MET, PIK3CA, PIK3R1	57	78	6879	9,28	0,063	0,0011	0,4840
**KEGG_PATHWAY**	hsa04110:Cell cycle	7	8,24	4,72E-04	CCND1, CDKN1B, EP300, PLK1, MDM2, ATR, TGFB2	57	124	6879	6,81	0,074	0,0013	0,5705

**KEGG_PATHWAY**	hsa04666:Fc gamma R-mediated phagocytosis	6	7,06	5,64E-04	PIK3CG, PIK3CB, PIK3CD, RAF1, PIK3CA, PIK3R1	57	84	6879	8,62	0,088	0,0015	0,6814
**KEGG_PATHWAY**	hsa04930:Type II diabetes mellitus	5	5,88	5,87E-04	PIK3CG, PIK3CB, PIK3CD, PIK3CA, PIK3R1	57	48	6879	12,57	0,091	0,0015	0,7093
**KEGG_PATHWAY**	hsa05144:Malaria	5	5,88	6,36E-04	IL6, SDC1, MET, THBS1, TGFB2	57	49	6879	12,31	0,098	0,0016	0,7675
**KEGG_PATHWAY**	hsa04070:Phosphatidylinositol signaling system	6	7,06	0,0011	PIK3CG, PIK3CB, PIK3CD, PIK3CA, PTEN, PIK3R1	57	98	6879	7,39	0,169	0,0029	1,3706
**KEGG_PATHWAY**	hsa04750:Inflammatory mediator regulation of TRP channels	6	7,06	0,0011	PIK3CG, PLA2G4A, PIK3CB, PIK3CD, PIK3CA, PIK3R1	57	98	6879	7,39	0,169	0,0029	1,3706
**KEGG_PATHWAY**	hsa04725:Cholinergic synapse	6	7,06	0,0020	PIK3CG, PIK3CB, BCL2, PIK3CD, PIK3CA, PIK3R1	57	111	6879	6,52	0,276	0,0049	2,3765
**KEGG_PATHWAY**	hsa00562:Inositol phosphate metabolism	5	5,88	0,0026	PIK3CG, PIK3CB, PIK3CD, PIK3CA, PTEN	57	71	6879	8,50	0,341	0,0062	3,0503

**KEGG_PATHWAY**	hsa05152:Tuberculosis	7	8,24	0,0030	CASP3, IL6, EP300, BCL2, RAF1, NFKB1, TGFB2	57	177	6879	4,77	0,385	0,0071	3,5489
**KEGG_PATHWAY**	hsa04152:AMPK signaling pathway	6	7,06	0,0031	PIK3CG, CCND1, PIK3CB, PIK3CD, PIK3CA, PIK3R1	57	123	6879	5,89	0,398	0,0073	3,6983
**KEGG_PATHWAY**	hsa04062:Chemokine signaling pathway	7	8,24	0,0038	PIK3CG, PIK3CB, PIK3CD, RAF1, PIK3CA, NFKB1, PIK3R1	57	186	6879	4,54	0,463	0,0088	4,5166
**KEGG_PATHWAY**	hsa04611:Platelet activation	6	7,06	0,0039	PIK3CG, PLA2G4A, PIK3CB, PIK3CD, PIK3CA, PIK3R1	57	130	6879	5,57	0,475	0,0090	4,6714
**KEGG_PATHWAY**	hsa04912:GnRH signaling pathway	5	5,88	0,0062	EGFR, PLA2G4A, RAF1, ELK1, MAP2K7	57	91	6879	6,63	0,639	0,0141	7,2920
**KEGG_PATHWAY**	hsa04921:Oxytocin signaling pathway	6	7,06	0,0072	EGFR, PLA2G4A, CCND1, PTGS2, RAF1, ELK1	57	150	6879	4,83	0,692	0,0160	8,3867
**KEGG_PATHWAY**	hsa04670:Leukocyte transendothelial migration	5	5,88	0,0140	PIK3CG, PIK3CB, PIK3CD, PIK3CA, PIK3R1	57	115	6879	5,25	0,899	0,0305	15,6628
**KEGG_P**	hsa05120:Epithelial cell signaling in	4	4,	0,0170	EGFR, CASP3, MET, NFKB1	57	67	6879	7,21	0,938	0,0365	18,7

**ATHWAY**	Helicobacter pylori infection		71									075
**KEGG_PATHWAY**	hsa05140:Leishmaniasis	4	4,71	0,0198	PTGS2, ELK1, NFKB1, TGFB2	57	71	6879	6,80	0,961	0,0419	21,4863
**KEGG_PATHWAY**	hsa04520:Adherens junction	4	4,71	0,0198	EGFR, FGFR1, EP300, MET	57	71	6879	6,80	0,961	0,0419	21,4863
**KEGG_PATHWAY**	hsa05020:Prion diseases	3	3,53	0,0309	IL6, ELK1, HSPA5	57	34	6879	10,65	0,994	0,0643	31,6435
**KEGG_PATHWAY**	hsa05202:Transcriptional misregulation in cancer	5	5,88	0,0464	IL6, CDKN1B, MET, MDM2, NFKB1	57	167	6879	3,61	0,999	0,0945	43,7441
**KEGG_PATHWAY**	hsa05145:Toxoplasmosis	4	4,71	0,0599	CASP3, BCL2, NFKB1, TGFB2	57	110	6879	4,39	0,999	0,1197	52,6833
**KEGG_PATHWAY**	hsa04726:Serotonergic synapse	4	4,71	0,0612	PLA2G4A, CASP3, PTGS2, RAF1	57	111	6879	4,35	0,999	0,1208	53,4818
**KEGG_PATHWAY**	hsa05134:Legionellosis	3	3,53	0,0712	CASP3, IL6, NFKB1	57	54	6879	6,70	0,999	0,1381	59,1189

KEGG_PATHWAY	hsa05321:Inflammatory bowel disease (IBD)	3	3,53	0,0953	IL6, NFKB1, TGFB2	57	64	6879	5,66	0,999	0,1806	70,2826

D. Signaling pathways in which the potential targets of miR-27a-3p are involved (identified by DAVID)

Table 1: targets identified by BIOCARTA (DAVID tool)

Table 2: targets identified by KEGG (DAVID tool)

Category	Term	Count	%	PValue	Genes	List Total	Pop Hits	Pop Total	Fold Enrichment	Bonferroni	Benjamini	FDR
**BIOCARTA**	h_keratinocytePathway:Keratinocyte Differentiation	5	9,43	0,0046	CEBPA, EGFR, BCL2, NFKB1, MAP2K7	25	48	1625	6,77	0,434	0,434	5,192
**BIOCARTA**	h_ctcfPathway:CTCF: First Multivalent Nuclear Factor	4	7,55	0,0053	PIK3CG, MDM2, PTEN, TGFB2	25	25	1625	10,4	0,477	0,277	5,904
**BIOCARTA**	h_tffPathway:Trefoil Factors Initiate Mucosal Healing	4	7,55	0,0059	PIK3CG, AKT1, EGFR, ERBB2	25	26	1625	10	0,517	0,215	6,588
**BIOCARTA**	h_hivnefPathway:HIV-I Nef: negative effector of Fas and TNF	5	9,43	0,0109	CASP3, BCL2, MDM2, NFKB1, MAP2K7	25	61	1625	5,33	0,739	0,285	11,842
**BIOCARTA**	h_metPathway:Signaling of Hepatocyte Growth Factor Receptor	4	7,55	0,0147	PIK3CG, PTK2B, MET, PTEN	25	36	1625	7,22	0,838	0,305	15,683

**BIOCARTA**	h_bcellsurvivalPathway:B Cell Survival Pathway	3	5,66	0,0195	PIK3CG, AKT1, CASP3	25	15	1625	13	0,912	0,333	20,339
**BIOCARTA**	h_achPathway:Role of nicotinic acetylcholine receptors in the regulation of apoptosis	3	5,66	0,0221	PIK3CG, AKT1, PTK2B	25	16	1625	12,19	0,936	0,325	22,732
**BIOCARTA**	h_hcmvPathway:Human Cytomegalovirus and Map Kinase Pathways	3	5,66	0,0248	PIK3CG, AKT1, NFKB1	25	17	1625	11,47	0,955	0,321	25,179
**BIOCARTA**	h_il7Pathway:IL-7 Signal Transduction	3	5,66	0,0248	PIK3CG, PTK2B, BCL2	25	17	1625	11,47	0,955	0,321	25,179
**BIOCARTA**	h_telPathway:Telomeres, Telomerase, Cellular Aging, and Immortality	3	5,66	0,0277	AKT1, EGFR, BCL2	25	18	1625	10,83	0,968	0,319	27,666
**BIOCARTA**	h_gcrpathway:Corticosteroids and cardioprotection	3	5,66	0,0307	PIK3CG, AKT1, NFKB1	25	19	1625	10,26	0,9783	0,318	30,183
**BIOCARTA**	h_chemicalPathway:Apoptotic Signaling in Response to DNA Damage	3	5,66	0,0403	AKT1, CASP3, BCL2	25	22	1625	8,86	0,994	0,369	37,802
**BIOCARTA**	h_aktPathway:AKT Signaling Pathway	3	5,66	0,0403	PIK3CG, AKT1, NFKB1	25	22	1625	8,86	0,994	0,369	37,802
**BIOCARTA**	h_her2Pathway:Role of ERBB2 in Signal Transduction and Oncology	3	5,66	0,0438	PIK3CG, EGFR, ERBB2	25	23	1625	8,48	0,996	0,368	40,331
**BIOCARTA**	h_rasPathway:Ras Signaling Pathway	3	5,66	0,0438	PIK3CG, AKT1, NFKB1	25	23	1625	8,48	0,996	0,368	40,331

**BIOCARTA**	h_badPathway:Regulation of BAD phosphorylation	3	5,66	0,0548	PIK3CG, AKT1, BCL2	25	26	1625	7,5	0,999	0,413	47,768
**BIOCARTA**	h_deathPathway:Induction of apoptosis through DR3 and DR4/5 Death Receptors	3	5,66	0,0836	CASP3, BCL2, NFKB1	25	33	1625	5,91	0,999	0,536	63,459
**BIOCARTA**	h_tsp1Pathway:TSP-1 Induced Apoptosis in Microvascular Endothelial Cell	2	3,77	0,0991	CASP3, THBS1	25	7	1625	18,57	0,999	0,575	69,977

Category	Term	Count	%	PValue	Genes	List Total	Pop Hits	Pop Total	Fold Enrichment	Bonferroni	Benjamini	FDR
**KEGG_PATHWAY**	hsa05200:Pathways in cancer	18	33,96	2,63507E-13	CEBPA, EGFR, PIK3CG, FGFR1, MSH2, ERBB2, MET, NFKB1, PTEN, SHH, DAPK1, TGFB2, AKT1, CASP3, BCL2, VEGFA, MDM2, PTCH1	34	393	6879	9,27	3,58E-11	3,58E-11	3,09E-10
**KEGG_PATHWAY**	hsa05205:Proteoglycans in cancer	14	26,42	2,47317E-12	PIK3CG, EGFR, FGFR1, ERBB2, MET, IGF2, TGFB2, AKT1, SDC1, CASP3, VEGFA, MDM2, PTCH1, THBS1	34	200	6879	14,16	3,36E-10	1,68E-10	2,90E-09
**KEGG_PATHWAY**	hsa05206:MicroRNAs in cancer	13	24,53	3,44165E-09	EGFR, CASP3, BCL2, ERBB2, VEGFA, MET, DNMT1, MDM2, NFKB1, STMN1, THBS1, PTEN, TGFB2	34	286	6879	9,20	4,68E-07	1,56E-07	4,04E-06

**KEGG_PATHWAY**	hsa05215:Prostate cancer	9	16,98	5,55534E-09	PIK3CG, AKT1, EGFR, FGFR1, BCL2, ERBB2, MDM2, NFKB1, PTEN	34	88	6879	20,69	7,56E-07	1,89E-07	6,52E-06
**KEGG_PATHWAY**	hsa04151:PI3K-Akt signaling pathway	12	22,64	3,08163E-07	PIK3CG, AKT1, EGFR, FGFR1, BCL2, VEGFA, MET, MDM2, NFKB1, THBS1, PTEN, EPHA2	34	345	6879	7,04	4,19E-05	8,38E-06	3,62E-04
**KEGG_PATHWAY**	hsa05230:Central carbon metabolism in cancer	7	13,21	4,64853E-07	PIK3CG, AKT1, EGFR, FGFR1, ERBB2, MET, PTEN	34	64	6879	22,13	6,32E-05	1,05E-05	5,46E-04
**KEGG_PATHWAY**	hsa05212:Pancreatic cancer	7	13,21	5,10396E-07	PIK3CG, AKT1, EGFR, ERBB2, VEGFA, NFKB1, TGFB2	34	65	6879	21,79	6,94E-05	9,92E-06	5,99E-04
**KEGG_PATHWAY**	hsa05218:Melanoma	7	13,21	8,67192E-07	PIK3CG, AKT1, EGFR, FGFR1, MET, MDM2, PTEN	34	71	6879	19,95	1,18E-04	1,47E-05	0,0010
**KEGG_PATHWAY**	hsa05219:Bladder cancer	6	11,32	1,22766E-06	EGFR, ERBB2, VEGFA, MDM2, THBS1, DAPK1	34	41	6879	29,61	1,67E-04	1,86E-05	0,0014
**KEGG_PATHWAY**	hsa04510:Focal adhesion	9	16,98	4,11386E-06	PIK3CG, AKT1, EGFR, BCL2, ERBB2, VEGFA, MET, THBS1, PTEN	34	206	6879	8,84	5,59E-04	5,59E-05	0,0048

**KEGG_PATHWAY**	hsa05161:Hepatitis B	8	15,09	4,31759E-06	PIK3CG, AKT1, CASP3, PTK2B, BCL2, NFKB1, PTEN, TGFB2	34	145	6879	11,16	5,87E-04	5,34E-05	0,0051
**KEGG_PATHWAY**	hsa04066:HIF-1 signaling pathway	7	13,21	5,15754E-06	PIK3CG, AKT1, EGFR, BCL2, ERBB2, VEGFA, NFKB1	34	96	6879	14,75	7,01E-04	5,85E-05	0,0061
**KEGG_PATHWAY**	hsa05210:Colorectal cancer	6	11,32	9,86938E-06	PIK3CG, AKT1, CASP3, MSH2, BCL2, TGFB2	34	62	6879	19,58	0,001	1,03E-04	0,0116
**KEGG_PATHWAY**	hsa05222:Small cell lung cancer	6	11,32	4,62587E-05	PIK3CG, AKT1, FHIT, BCL2, NFKB1, PTEN	34	85	6879	14,28	0,006	4,49E-04	0,0543
**KEGG_PATHWAY**	hsa04015:Rap1 signaling pathway	8	15,09	4,85732E-05	PIK3CG, AKT1, EGFR, FGFR1, VEGFA, MET, THBS1, EPHA2	34	210	6879	7,71	0,007	4,40E-04	0,0570
**KEGG_PATHWAY**	hsa04014:Ras signaling pathway	8	15,09	7,75109E-05	PIK3CG, AKT1, EGFR, FGFR1, VEGFA, MET, NFKB1, EPHA2	34	226	6879	7,16	0,010	6,59E-04	0,0910
**KEGG_PATHWAY**	hsa05213:Endometrial cancer	5	9,43	0,0001	PIK3CG, AKT1, EGFR, ERBB2, PTEN	34	52	6879	19,45	0,014	8,08E-04	0,1186
**KEGG_PAT**	hsa05223:Non-small cell lung cancer	5	9,43	0,0001	PIK3CG, AKT1, EGFR, FHIT, ERBB2	34	56	6879	18,06	0,018	0,0010	0,1587

**HWAY**												
**KEGG_PATHWAY**	hsa04010:MAPK signaling pathway	8	15,09	0,0002	AKT1, EGFR, FGFR1, CASP3, NFKB1, STMN1, MAP2K7, TGFB2	34	253	6879	6,40	0,021	0,0011	0,1847
**KEGG_PATHWAY**	hsa04210:Apoptosis	5	9,43	0,0002	PIK3CG, AKT1, CASP3, BCL2, NFKB1	34	62	6879	16,32	0,027	0,0014	0,2361
**KEGG_PATHWAY**	hsa05214:Glioma	5	9,43	0,0002	PIK3CG, AKT1, EGFR, MDM2, PTEN	34	65	6879	15,56	0,032	0,0016	0,2835
**KEGG_PATHWAY**	hsa05211:Renal cell carcinoma	5	9,43	0,0003	PIK3CG, AKT1, VEGFA, MET, TGFB2	34	66	6879	15,33	0,034	0,0016	0,3008
**KEGG_PATHWAY**	hsa05169:Epstein-Barr virus infection	6	11,32	0,0003	PIK3CG, AKT1, BCL2, MDM2, NFKB1, MAP2K7	34	122	6879	9,95	0,034	0,0015	0,3021
**KEGG_PATHWAY**	hsa05220:Chronic myeloid leukemia	5	9,43	0,0004	PIK3CG, AKT1, MDM2, NFKB1, TGFB2	34	72	6879	14,05	0,048	0,0020	0,4205
**KEGG_PATHWAY**	hsa04068:FoxO signaling pathway	6	11,32	0,0004	PIK3CG, AKT1, EGFR, MDM2, PTEN, TGFB2	34	134	6879	9,06	0,053	0,0022	0,4667

**KEGG_PATHWAY**	hsa04012:ErbB signaling pathway	5	9,43	0,0007	PIK3CG, AKT1, EGFR, ERBB2, MAP2K7	34	87	6879	11,63	0,096	0,0039	0,8632
**KEGG_PATHWAY**	hsa05144:Malaria	4	7,55	0,0016	SDC1, MET, THBS1, TGFB2	34	49	6879	16,52	0,195	0,0080	1,8562
**KEGG_PATHWAY**	hsa04668:TNF signaling pathway	5	9,43	0,0016	PIK3CG, AKT1, CASP3, NFKB1, MAP2K7	34	107	6879	9,45	0,196	0,0077	1,8622
**KEGG_PATHWAY**	hsa05145:Toxoplasmosis	5	9,43	0,0018	AKT1, CASP3, BCL2, NFKB1, TGFB2	34	110	6879	9,20	0,214	0,0083	2,0604
**KEGG_PATHWAY**	hsa05221:Acute myeloid leukemia	4	7,55	0,0023	PIK3CG, CEBPA, AKT1, NFKB1	34	56	6879	14,45	0,273	0,0106	2,7187
**KEGG_PATHWAY**	hsa04722:Neurotrophin signaling pathway	5	9,43	0,0024	PIK3CG, AKT1, BCL2, NFKB1, MAP2K7	34	120	6879	8,43	0,282	0,0107	2,8244
**KEGG_PATHWAY**	hsa04071:Sphingolipid signaling pathway	5	9,43	0,0024	PIK3CG, AKT1, BCL2, NFKB1, PTEN	34	120	6879	8,43	0,282	0,0107	2,8244
**KEGG_PAT**	hsa04380:Osteoclast differentiation	5	9,43	0,0034	PIK3CG, AKT1, NFKB1, MAP2K7, TGFB2	34	131	6879	7,72	0,366	0,0142	3,8631

**HWAY**												
**KEGG_PATHWAY**	hsa05120:Epithelial cell signaling in Helicobacter pylori infection	4	7,55	0,0039	EGFR, CASP3, MET, NFKB1	34	67	6879	12,08	0,413	0,0160	4,4953
**KEGG_PATHWAY**	hsa04115:p53 signaling pathway	4	7,55	0,0039	CASP3, MDM2, THBS1, PTEN	34	67	6879	12,08	0,413	0,0160	4,4953
**KEGG_PATHWAY**	hsa04520:Adherens junction	4	7,55	0,0046	EGFR, FGFR1, ERBB2, MET	34	71	6879	11,40	0,466	0,0183	5,2744
**KEGG_PATHWAY**	hsa04932:Non-alcoholic fatty liver disease (NAFLD)	5	9,43	0,0056	PIK3CG, CEBPA, AKT1, CASP3, NFKB1	34	151	6879	6,70	0,532	0,0214	6,3397
**KEGG_PATHWAY**	hsa05152:Tuberculosis	5	9,43	0,0097	AKT1, CASP3, BCL2, NFKB1, TGFB2	34	177	6879	5,72	0,734	0,0361	10,7930
**KEGG_PATHWAY**	hsa04660:T cell receptor signaling pathway	4	7,55	0,0119	PIK3CG, AKT1, NFKB1, MAP2K7	34	100	6879	8,09	0,803	0,0429	13,0670
**KEGG_PATHWAY**	hsa05142:Chagas disease (American trypanosomiasis)	4	7,55	0,0132	PIK3CG, AKT1, NFKB1, TGFB2	34	104	6879	7,78	0,835	0,0464	14,4249

**KEGG_PATHWAY**	hsa04620:Toll-like receptor signaling pathway	4	7,55	0,0139	PIK3CG, AKT1, NFKB1, MAP2K7	34	106	6879	7,63	0,850	0,0476	15,1275
**KEGG_PATHWAY**	hsa05146:Amoebiasis	4	7,55	0,0139	PIK3CG, CASP3, NFKB1, TGFB2	34	106	6879	7,63	0,850	0,0476	15,1275
**KEGG_PATHWAY**	hsa04931:Insulin resistance	4	7,55	0,0146	PIK3CG, AKT1, NFKB1, PTEN	34	108	6879	7,49	0,864	0,0487	15,8454
**KEGG_PATHWAY**	hsa05160:Hepatitis C	4	7,55	0,0253	PIK3CG, AKT1, EGFR, NFKB1	34	133	6879	6,08	0,969	0,0814	25,9465
**KEGG_PATHWAY**	hsa04150:mTOR signaling pathway	3	5,66	0,0312	PIK3CG, AKT1, PTEN	34	58	6879	10,47	0,986	0,0976	31,0800
**KEGG_PATHWAY**	hsa04370:VEGF signaling pathway	3	5,66	0,0342	PIK3CG, AKT1, VEGFA	34	61	6879	9,95	0,991	0,1044	33,5735
**KEGG_PATHWAY**	hsa04664:Fc epsilon RI signaling pathway	3	5,66	0,0418	PIK3CG, AKT1, MAP2K7	34	68	6879	8,93	0,996	0,1235	39,3908
**KEGG_PAT**	hsa04662:B cell receptor signaling pathway	3	5,66	0,0429	PIK3CG, AKT1, NFKB1	34	69	6879	8,80	0,997	0,1240	40,2169

**HWAY**												
**KEGG_PATHWAY**	hsa05202:Transcriptional misregulation in cancer	4	7,55	0,0450	CEBPA, MET, MDM2, NFKB1	34	167	6879	4,85	0,998	0,1274	41,7822
**KEGG_PATHWAY**	hsa04917:Prolactin signaling pathway	3	5,66	0,0452	PIK3CG, AKT1, NFKB1	34	71	6879	8,55	0,998	0,1251	41,8625
**KEGG_PATHWAY**	hsa05164:Influenza A	4	7,55	0,0498	PIK3CG, AKT1, NFKB1, MAP2K7	34	174	6879	4,65	0,999	0,1349	45,1322
**KEGG_PATHWAY**	hsa04062:Chemokine signaling pathway	4	7,55	0,0587	PIK3CG, AKT1, PTK2B, NFKB1	34	186	6879	4,35	0,999	0,1545	50,8157
**KEGG_PATHWAY**	hsa04024:cAMP signaling pathway	4	7,55	0,0682	PIK3CG, AKT1, NFKB1, PTCH1	34	198	6879	4,09	0,999	0,1747	56,3392
**KEGG_PATHWAY**	hsa04912:GnRH signaling pathway	3	5,66	0,0702	EGFR, PTK2B, MAP2K7	34	91	6879	6,67	0,999	0,1763	57,4209
**KEGG_PATHWAY**	hsa05203:Viral carcinogenesis	4	7,55	0,0740	PIK3CG, CASP3, MDM2, NFKB1	34	205	6879	3,95	0,999	0,1822	59,4526

**KEGG_PATHWAY**	hsa04915:Estrogen signaling pathway	3	5,66	0,0812	PIK3CG, AKT1, EGFR	34	99	6879	6,13	0,999	0,1953	62,9845
**KEGG_PATHWAY**	hsa05231:Choline metabolism in cancer	3	5,66	0,0840	PIK3CG, AKT1, EGFR	34	101	6879	6,01	0,999	0,1983	64,3041
**KEGG_PATHWAY**	hsa04725:Cholinergic synapse	3	5,66	0,0986	PIK3CG, AKT1, BCL2	34	111	6879	5,47	0,999	0,2265	70,4511

E. Signaling pathways in which the potential targets of miR-143-5p are involved (identified by DAVID)

Table 1: targets identified by BIOCARTA (DAVID tool)

Table 2: targets identified by KEGG (DAVID tool)

Category	Term	Count	%	PValue	Genes	List Total	Pop Hits	Pop Total	Fold Enrichment	Bonferroni	Benjamini	FDR
BIOCARTA	h_hivnefPathway:HIV-I Nef: negative effector of Fas and TNF	7	10,45	9,22E-04	NUMA1, CASP3, TNF, BCL2, LMNA, MDM2, MAP2K7	33	61	1625	5,65	0,122	0,1220	1,0840
BIOCARTA	h_ctcfPathway:CTCF: First Multivalent Nuclear Factor	5	7,46	0,0012	TP53, PIK3CA, MDM2, PTEN, TGFB2	33	25	1625	9,85	0,153	0,0795	1,3777
BIOCARTA	h_g2Pathway:Cell Cycle: G2/M Checkpoint	5	7,46	0,0012	CDKN1A, PLK1, TP53, MDM2, ATR	33	25	1625	9,85	0,153	0,0795	1,3777
BIOCARTA	h_p53hypoxiaPathway:Hypoxia and p53 in the Cardiovascular system	5	7,46	0,0014	AKT1, CDKN1A, HIF1A, TP53, MDM2	33	26	1625	9,47	0,176	0,0623	1,6040
BIOCARTA	h_p53Pathway:p53 Signaling Pathway	4	5,97	0,0039	CDKN1A, BCL2, TP53, MDM2	33	17	1625	11,59	0,425	0,1291	4,5264
BIOCARTA	h_telPathway:Telomeres, Telomerase, Cellular Aging, and Immortality	4	5,97	0,0046	AKT1, EGFR, BCL2, TP53	33	18	1625	10,94	0,480	0,1227	5,3386

**BIOCARTA**	h_chemicalPathway:Apoptotic Signaling in Response to DNA Damage	4	5,97	0,0083	AKT1, CASP3, BCL2, TP53	33	22	1625	8,95	0,691	0,1776	9,3640
**BIOCARTA**	h_mtorPathway:mTOR Signaling Pathway	4	5,97	0,0133	AKT1, TSC2, PIK3CA, PTEN	33	26	1625	7,58	0,848	0,2358	14,5931
**BIOCARTA**	h_tffPathway:Trefoil Factors Initiate Mucosal Healing	4	5,97	0,0133	AKT1, EGFR, ERBB2, PIK3CA	33	26	1625	7,58	0,848	0,2358	14,5931
**BIOCARTA**	h_g1Pathway:Cell Cycle: G1/S Check Point	4	5,97	0,0196	CDKN1A, TP53, ATR, TGFB2	33	30	1625	6,57	0,939	0,2950	20,8855
**BIOCARTA**	h_metPathway:Signaling of Hepatocyte Growth Factor Receptor	4	5,97	0,0319	PTK2B, MET, PIK3CA, PTEN	33	36	1625	5,47	0,990	0,3981	31,8030
**BIOCARTA**	h_bcellsurvivalPathway:B Cell Survival Pathway	3	4,48	0,0336	AKT1, CASP3, PIK3CA	33	15	1625	9,85	0,992	0,3827	33,2446
**BIOCARTA**	h_achPathway:Role of nicotinic acetylcholine receptors in the regulation of apoptosis	3	4,48	0,0380	AKT1, PTK2B, PIK3CA	33	16	1625	9,23	0,996	0,3911	36,6999
**BIOCARTA**	h_hsp27Pathway:Stress Induction of HSP Regulation	3	4,48	0,0380	CASP3, TNF, BCL2	33	16	1625	9,23	0,996	0,3911	36,6999
**BIOCARTA**	h_il7Pathway:IL-7 Signal Transduction	3	4,48	0,0425	PTK2B, BCL2, PIK3CA	33	17	1625	8,69	0,998	0,3997	40,1407
**BIOCARTA**	h_ptenPathway:PTEN dependent cell cycle arrest and apoptosis	3	4,48	0,0472	AKT1, PIK3CA, PTEN	33	18	1625	8,21	0,998	0,4084	43,5454

**BIOCARTA**	h_arfPathway:Tumor Suppressor Arf Inhibits Ribosomal Biogenesis	3	4,48	0,0472	TP53, PIK3CA, MDM2	33	18	1625	8,21	0,998	0,4084	43,5454
**BIOCARTA**	h_igf1mtorPathway:Skeletal muscle hypertrophy is regulated via AKT/mTOR pathway	3	4,48	0,0573	AKT1, PIK3CA, PTEN	33	20	1625	7,39	0,999	0,4478	50,1710
**BIOCARTA**	h_atmPathway:ATM Signaling Pathway	3	4,48	0,0625	CDKN1A, TP53, MDM2	33	21	1625	7,03	0,999	0,4550	53,3602
**BIOCARTA**	h_keratinocytePathway:Keratinocyte Differentiation	4	5,97	0,0659	EGFR, TNF, BCL2, MAP2K7	33	48	1625	4,10	0,999	0,4515	55,2998
**BIOCARTA**	h_her2Pathway:Role of ERBB2 in Signal Transduction and Oncology	3	4,48	0,0735	EGFR, ERBB2, PIK3CA	33	23	1625	6,42	0,999	0,4692	59,4298
**BIOCARTA**	h_eif4Pathway:Regulation of eIF4e and p70 S6 Kinase	3	4,48	0,0792	AKT1, PIK3CA, PTEN	33	24	1625	6,16	0,999	0,4762	62,2919
**BIOCARTA**	h_badPathway:Regulation of BAD phosphorylation	3	4,48	0,0911	AKT1, BCL2, PIK3CA	33	26	1625	5,68	0,999	0,5077	67,6393
**BIOCARTA**	h_raccycdPathway:Influence of Ras and Rho proteins on G1 to S Transition	3	4,48	0,0972	AKT1, CDKN1A, PIK3CA	33	27	1625	5,47	0,999	0,5136	70,1173

Category	Term	Count	%	PValue	Genes	List Total	Pop Hits	Pop Total	Fold Enrichment	Bonferroni	Benjamini	FDR
**KEGG_PATHWAY**	hsa05205:Proteoglycans in cancer	19	28,36	5,23E-17	EGFR, FGFR1, TNF, ERBB2, MET, PIK3CD, TP53, IGF2, TGFB2, AKT1, CASP3, CDKN1A, SDC1, HIF1A, CD44, VEGFA, MDM2, PIK3CA, PTCH1	45	200	6879	14,52	7,90E-15	7,90E-15	6,26E-14
**KEGG_PATHWAY**	hsa05200:Pathways in cancer	22	32,84	2,72E-15	EGFR, FGFR1, AR, MSH2, ERBB2, PIK3CD, MET, TP53, CXCL8, PTEN, TGFB2, DAPK1, AKT1, CASP3, CDKN1A, HIF1A, RASSF1, BCL2, VEGFA, MDM2, PIK3CA, PTCH1	45	393	6879	8,56	4,19E-13	2,10E-13	3,32E-12
**KEGG_PATHWAY**	hsa05215:Prostate cancer	12	17,91	4,30E-12	AKT1, EGFR, FGFR1, AR, CDKN1A, BCL2, ERBB2, PIK3CD, TP53, PIK3CA, MDM2, PTEN	45	88	6879	20,85	6,50E-10	2,17E-10	5,14E-09
**KEGG_PATHWAY**	hsa05206:MicroRNAs in cancer	16	23,88	1,03E-10	EGFR, ERBB2, MET, EZH2, TP53, PTEN, TGFB2, CDKN1A, CASP3, CD44, RASSF1, BCL2, VEGFA, MDM2, DNMT1, STMN1	45	286	6879	8,55	1,56E-08	3,90E-09	1,23E-07
**KEGG_PATHWAY**	hsa05219:Bladder cancer	9	13,43	1,17E-10	EGFR, CDKN1A, RASSF1, ERBB2, VEGFA, TP53, CXCL8, MDM2, DAPK1	45	41	6879	33,56	1,77E-08	3,54E-09	1,40E-07

**KEGG_PATHWAY**	hsa05230:Central carbon metabolism in cancer	10	14,93	1,60E-10	AKT1, EGFR, FGFR1, HIF1A, ERBB2, PIK3CD, MET, TP53, PIK3CA, PTEN	45	64	6879	23,89	2,42E-08	4,03E-09	1,92E-07
**KEGG_PATHWAY**	hsa05218:Melanoma	10	14,93	4,20E-10	AKT1, EGFR, FGFR1, CDKN1A, PIK3CD, MET, TP53, PIK3CA, MDM2, PTEN	45	71	6879	21,53	6,34E-08	9,05E-09	5,02E-07
**KEGG_PATHWAY**	hsa05161:Hepatitis B	12	17,91	1,05E-09	AKT1, CDKN1A, CASP3, TNF, PTK2B, BCL2, PIK3CD, TP53, CXCL8, PIK3CA, PTEN, TGFB2	45	145	6879	12,65	1,59E-07	1,99E-08	1,26E-06
**KEGG_PATHWAY**	hsa05223:Non-small cell lung cancer	8	11,94	4,89E-08	AKT1, EGFR, FHIT, RASSF1, ERBB2, PIK3CD, TP53, PIK3CA	45	56	6879	21,84	7,38E-06	8,20E-07	5,85E-05
**KEGG_PATHWAY**	hsa05210:Colorectal cancer	8	11,94	1,01E-07	AKT1, CASP3, MSH2, BCL2, PIK3CD, TP53, PIK3CA, TGFB2	45	62	6879	19,72	1,52E-05	1,52E-06	1,20E-04
**KEGG_PATHWAY**	hsa04066:HIF-1 signaling pathway	9	13,43	1,26E-07	AKT1, EGFR, CDKN1A, HIF1A, BCL2, ERBB2, PIK3CD, VEGFA, PIK3CA	45	96	6879	14,33	1,90E-05	1,73E-06	1,50E-04

**KEGG_PATHWAY**	hsa05214:Glioma	8	11,94	1,41E-07	AKT1, EGFR, CDKN1A, PIK3CD, TP53, PIK3CA, MDM2, PTEN	45	65	6879	18,81	2,12E-05	1,77E-06	1,68E-04
**KEGG_PATHWAY**	hsa05212:Pancreatic cancer	8	11,94	1,41E-07	AKT1, EGFR, ERBB2, PIK3CD, VEGFA, TP53, PIK3CA, TGFB2	45	65	6879	18,81	2,12E-05	1,77E-06	1,68E-04
**KEGG_PATHWAY**	hsa05213:Endometrial cancer	7	10,45	7,86E-07	AKT1, EGFR, ERBB2, PIK3CD, TP53, PIK3CA, PTEN	45	52	6879	20,58	1,19E-04	9,13E-06	9,40E-04
**KEGG_PATHWAY**	hsa05169:Epstein-Barr virus infection	9	13,43	8,08E-07	AKT1, CDKN1A, CD44, BCL2, PIK3CD, TP53, PIK3CA, MDM2, MAP2K7	45	122	6879	11,28	1,22E-04	8,71E-06	9,65E-04
**KEGG_PATHWAY**	hsa04151:PI3K-Akt signaling pathway	13	19,40	1,03E-06	EGFR, FGFR1, PIK3CD, MET, TP53, PTEN, AKT1, CDKN1A, BCL2, VEGFA, TSC2, MDM2, PIK3CA	45	345	6879	5,76	1,56E-04	1,04E-05	0,0012
**KEGG_PATHWAY**	hsa04068:FoxO signaling pathway	9	13,43	1,65E-06	AKT1, EGFR, CDKN1A, PLK1, PIK3CD, PIK3CA, MDM2, PTEN, TGFB2	45	134	6879	10,27	2,49E-04	1,56E-05	0,0020

**KEGG_PATHWAY**	hsa04210:Apoptosis	7	10,45	2,26E-06	AKT1, CASP3, TNF, BCL2, PIK3CD, TP53, PIK3CA	45	62	6879	17,26	3,42E-04	2,01E-05	0,0027
**KEGG_PATHWAY**	hsa05211:Renal cell carcinoma	7	10,45	3,28E-06	AKT1, HIF1A, PIK3CD, VEGFA, MET, PIK3CA, TGFB2	45	66	6879	16,21	4,96E-04	2,75E-05	0,0039
**KEGG_PATHWAY**	hsa04115:p53 signaling pathway	7	10,45	3,59E-06	CDKN1A, CASP3, TSC2, TP53, MDM2, ATR, PTEN	45	67	6879	15,97	5,42E-04	2,85E-05	0,0043
**KEGG_PATHWAY**	hsa05220:Chronic myeloid leukemia	7	10,45	5,49E-06	AKT1, CDKN1A, PIK3CD, TP53, PIK3CA, MDM2, TGFB2	45	72	6879	14,86	8,28E-04	4,14E-05	0,0066
**KEGG_PATHWAY**	hsa05222:Small cell lung cancer	7	10,45	1,44E-05	AKT1, FHIT, BCL2, PIK3CD, TP53, PIK3CA, PTEN	45	85	6879	12,59	0,002	1,04E-04	0,0173
**KEGG_PATHWAY**	hsa04012:ErbB signaling pathway	7	10,45	1,65E-05	AKT1, EGFR, CDKN1A, ERBB2, PIK3CD, PIK3CA, MAP2K7	45	87	6879	12,30	0,002	1,13E-04	0,0197

**KEGG_PATHWAY**	hsa05160:Hepatitis C	8	11,94	1,82E-05	AKT1, EGFR, CDKN1A, TNF, PIK3CD, TP53, CXCL8, PIK3CA	45	133	6879	9,19	0,003	1,19E-04	0,0218
**KEGG_PATHWAY**	hsa04150:mTOR signaling pathway	6	8,96	3,02E-05	AKT1, TNF, PIK3CD, TSC2, PIK3CA, PTEN	45	58	6879	15,81	0,005	1,90E-04	0,0361
**KEGG_PATHWAY**	hsa04510:Focal adhesion	9	13,43	3,96E-05	AKT1, EGFR, BCL2, ERBB2, PIK3CD, VEGFA, MET, PIK3CA, PTEN	45	206	6879	6,68	0,006	2,39E-04	0,0473
**KEGG_PATHWAY**	hsa04919:Thyroid hormone signaling pathway	7	10,45	8,05E-05	AKT1, HIF1A, PIK3CD, TSC2, TP53, PIK3CA, MDM2	45	115	6879	9,30	0,012	4,68E-04	0,0962
**KEGG_PATHWAY**	hsa04071:Sphingolipid signaling pathway	7	10,45	1,02E-04	AKT1, TNF, BCL2, PIK3CD, TP53, PIK3CA, PTEN	45	120	6879	8,92	0,015	5,71E-04	0,1220
**KEGG_PATHWAY**	hsa04380:Osteoclast differentiation	7	10,45	1,66E-04	AKT1, CYBB, TNF, PIK3CD, PIK3CA, MAP2K7, TGFB2	45	131	6879	8,17	0,025	8,94E-04	0,1980
**KEGG_PA**	hsa04010:MAPK signaling pathway	9	13,	1,68	AKT1, EGFR, FGFR1, CASP3, TNF, TP53, STMN1, MAP2K7, TGFB2	45	253	6879	5,44	0,025	8,75E-04	0,2009

**THWAY**			43	E-04								
**KEGG_PATHWAY**	hsa05144:Malaria	5	7,46	2,50E-04	SDC1, TNF, MET, CXCL8, TGFB2	45	49	6879	15,60	0,037	0,013	0,2987
**KEGG_PATHWAY**	hsa05231:Choline metabolism in cancer	6	8,96	4,26E-04	AKT1, EGFR, HIF1A, PIK3CD, TSC2, PIK3CA	45	101	6879	9,08	0,062	0,021	0,5081
**KEGG_PATHWAY**	hsa05142:Chagas disease (American trypanosomiasis)	6	8,96	4,88E-04	AKT1, TNF, PIK3CD, CXCL8, PIK3CA, TGFB2	45	104	6879	8,82	0,071	0,023	0,5814
**KEGG_PATHWAY**	hsa04014:Ras signaling pathway	8	11,94	5,11E-04	AKT1, EGFR, FGFR1, RASSF1, PIK3CD, VEGFA, MET, PIK3CA	45	226	6879	5,41	0,074	0,023	0,6095
**KEGG_PATHWAY**	hsa04620:Toll-like receptor signaling pathway	6	8,96	5,32E-04	AKT1, TNF, PIK3CD, CXCL8, PIK3CA, MAP2K7	45	106	6879	8,65	0,077	0,024	0,6345
**KEGG_PATHWAY**	hsa05146:Amoebiasis	6	8,96	5,32E-04	CASP3, TNF, PIK3CD, CXCL8, PIK3CA, TGFB2	45	106	6879	8,65	0,077	0,024	0,6345
**KEGG_PATHWAY**	hsa04668:TNF signaling pathway	6	8,96	5,56E-04	AKT1, CASP3, TNF, PIK3CD, PIK3CA, MAP2K7	45	107	6879	8,57	0,081	0,024	0,6623

**KEGG_PATHWAY**	hsa04664:Fc epsilon RI signaling pathway	5	7,46	8,80E-04	AKT1, TNF, PIK3CD, PIK3CA, MAP2K7	45	68	6879	11,24	0,125	0,0037	1,0476
**KEGG_PATHWAY**	hsa04722:Neurotrophin signaling pathway	6	8,96	9,37E-04	AKT1, BCL2, PIK3CD, TP53, PIK3CA, MAP2K7	45	120	6879	7,64	0,132	0,0038	1,1143
**KEGG_PATHWAY**	hsa05166:HTLV-I infection	8	11,94	0,0010	AKT1, CDKN1A, TNF, PIK3CD, TP53, PIK3CA, ATR, TGFB2	45	254	6879	4,81	0,143	0,0041	1,2171
**KEGG_PATHWAY**	hsa04110:Cell cycle	6	8,96	0,0011	CDKN1A, PLK1, TP53, MDM2, ATR, TGFB2	45	124	6879	7,40	0,151	0,0042	1,2907
**KEGG_PATHWAY**	hsa04015:Rap1 signaling pathway	7	10,45	0,0020	AKT1, EGFR, FGFR1, PIK3CD, VEGFA, MET, PIK3CA	45	210	6879	5,10	0,263	0,0076	2,3859
**KEGG_PATHWAY**	hsa04932:Non-alcoholic fatty liver disease (NAFLD)	6	8,96	0,0026	AKT1, CASP3, TNF, PIK3CD, CXCL8, PIK3CA	45	151	6879	6,07	0,325	0,0095	3,0621
**KEGG_PATHWAY**	hsa04660:T cell receptor signaling pathway	5	7,46	0,0037	AKT1, TNF, PIK3CD, PIK3CA, MAP2K7	45	100	6879	7,64	0,425	0,0131	4,2835

**KEGG_PATHWAY**	hsa05014:Amyotrophic lateral sclerosis (ALS)	4	5,97	0,0039	CASP3, TNF, BCL2, TP53	45	50	6879	12,23	0,444	0,0136	4,5426
**KEGG_PATHWAY**	hsa05164:Influenza A	6	8,96	0,0048	AKT1, TNF, PIK3CD, CXCL8, PIK3CA, MAP2K7	45	174	6879	5,27	0,515	0,0163	5,5648
**KEGG_PATHWAY**	hsa04931:Insulin resistance	5	7,46	0,0048	AKT1, TNF, PIK3CD, PIK3CA, PTEN	45	108	6879	7,08	0,517	0,0161	5,6061
**KEGG_PATHWAY**	hsa05145:Toxoplasmosis	5	7,46	0,0051	AKT1, CASP3, TNF, BCL2, TGFB2	45	110	6879	6,95	0,541	0,0168	5,9731
**KEGG_PATHWAY**	hsa04370:VEGF signaling pathway	4	5,97	0,0068	AKT1, PIK3CD, VEGFA, PIK3CA	45	61	6879	10,02	0,642	0,0216	7,8193
**KEGG_PATHWAY**	hsa04650:Natural killer cell mediated cytotoxicity	5	7,46	0,0074	CASP3, TNF, PTK2B, PIK3CD, PIK3CA	45	122	6879	6,27	0,674	0,0231	8,4927
**KEGG_PATHWAY**	hsa05120:Epithelial cell signaling in Helicobacter pylori infection	4	5,97	0,0088	EGFR, CASP3, MET, CXCL8	45	67	6879	9,13	0,737	0,0269	10,0250

**KEGG_PATHWAY**	hsa05203:Viral carcinogenesis	6	8,996	0,0095	CDKN1A, CASP3, PIK3CD, TP53, PIK3CA, MDM2	45	205	6879	4,47	0,762	0,0283	10,7356
**KEGG_PATHWAY**	hsa04520:Adherens junction	4	5,997	0,0103	EGFR, FGFR1, ERBB2, MET	45	71	6879	8,61	0,791	0,0302	11,6550
**KEGG_PATHWAY**	hsa04914:Progesterone-mediated oocyte maturation	4	5,997	0,0178	AKT1, PLK1, PIK3CD, PIK3CA	45	87	6879	7,03	0,934	0,0509	19,3424
**KEGG_PATHWAY**	hsa05323:Rheumatoid arthritis	4	5,997	0,0184	TNF, VEGFA, CXCL8, TGFB2	45	88	6879	6,95	0,939	0,0514	19,8786
**KEGG_PATHWAY**	hsa05202:Transcriptional misregulation in cancer	5	7,496	0,0214	CDKN1A, MET, TP53, CXCL8, MDM2	45	167	6879	4,58	0,962	0,0587	22,7842
**KEGG_PATHWAY**	hsa04915:Estrogen signaling pathway	4	5,997	0,0250	AKT1, EGFR, PIK3CD, PIK3CA	45	99	6879	6,18	0,978	0,0672	26,1279
**KEGG_PATHWAY**	hsa05152:Tuberculosis	5	7,496	0,0258	AKT1, CASP3, TNF, BCL2, TGFB2	45	177	6879	4,32	0,981	0,0682	26,8756
**KEGG_PA**	hsa04973:Carbohydrate digestion and absorption	3	4,	0,0293	AKT1, PIK3CD, PIK3CA	45	42	6879	10,92	0,989	0,0757	29,9

**THWAY**			48									058
**KEGG_PATHWAY**	hsa04062:Chemokine signaling pathway	5	7,46	0,0303	AKT1, PTK2B, PIK3CD, CXCL8, PIK3CA	45	186	6879	4,11	0,990	0,0770	30,7688
**KEGG_PATHWAY**	hsa04725:Cholinergic synapse	4	5,97	0,0336	AKT1, BCL2, PIK3CD, PIK3CA	45	111	6879	5,51	0,994	0,0837	33,5076
**KEGG_PATHWAY**	hsa04930:Type II diabetes mellitus	3	4,48	0,0375	TNF, PIK3CD, PIK3CA	45	48	6879	9,55	0,996	0,0916	36,6423
**KEGG_PATHWAY**	hsa04152:AMPK signaling pathway	4	5,97	0,0434	AKT1, PIK3CD, TSC2, PIK3CA	45	123	6879	4,97	0,998	0,1041	41,1966
**KEGG_PATHWAY**	hsa05134:Legionellosis	3	4,48	0,0464	CASP3, TNF, CXCL8	45	54	6879	8,49	0,999	0,1092	43,3267
**KEGG_PATHWAY**	hsa04923:Regulation of lipolysis in adipocytes	3	4,48	0,0495	AKT1, PIK3CD, PIK3CA	45	56	6879	8,19	0,999	0,1146	45,5156
**KEGG_PATHWAY**	hsa05221:Acute myeloid leukemia	3	4,48	0,0495	AKT1, PIK3CD, PIK3CA	45	56	6879	8,19	0,999	0,1146	45,5156

**KEGG_PATHWAY**	hsa05162:Measles	4	5,97	0,0527	AKT1, PIK3CD, TP53, PIK3CA	45	133	6879	4,60	0,999	0,1199	47,6347
**KEGG_PATHWAY**	hsa04910:Insulin signaling pathway	4	5,97	0,0576	AKT1, PIK3CD, TSC2, PIK3CA	45	138	6879	4,43	0,999	0,1288	50,8120
**KEGG_PATHWAY**	hsa04550:Signaling pathways regulating pluripotency of stem cells	4	5,97	0,0597	AKT1, FGFR1, PIK3CD, PIK3CA	45	140	6879	4,37	0,999	0,1313	52,0695
**KEGG_PATHWAY**	hsa04662:B cell receptor signaling pathway	3	4,48	0,0716	AKT1, PIK3CD, PIK3CA	45	69	6879	6,65	0,999	0,1543	58,8837
**KEGG_PATHWAY**	hsa00562:Inositol phosphate metabolism	3	4,48	0,0753	PIK3CD, PIK3CA, PTEN	45	71	6879	6,46	0,999	0,1595	60,7732
**KEGG_PATHWAY**	hsa04917:Prolactin signaling pathway	3	4,48	0,0753	AKT1, PIK3CD, PIK3CA	45	71	6879	6,46	0,999	0,1595	60,7732
**KEGG_PATHWAY**	hsa05133:Pertussis	3	4,48	0,0828	CASP3, TNF, CXCL8	45	75	6879	6,11	0,999	0,1722	64,3965
**KEGG_PA**	hsa05100:Bacterial invasion of epithelial cells	3	4,	0,0885	PIK3CD, MET, PIK3CA	45	78	6879	5,88	0,999	0,1812	66,9

THWAY			48									726
KEGG_PATHWAY	hsa05410:Hypertrophic cardiomyopathy (HCM)	3	4,48	0,0885	TNF, LMNA, TGFB2	45	78	6879	5,88	0,999	0,1812	66,9726

F. Signaling pathways in which the potential targets of miR-182-5p are involved (identified by DAVID)

Table 1: targets identified by BIOCARTA (DAVID tool)

Table 2: targets identified by KEGG (DAVID tool)

Category	Term	Count	%	PValue	Genes	List Total	Pop Hits	Pop Total	Fold Enrichment	Bonferroni	Benjamini	FDR
BIOCARTA	h_ctcfPathway:CTCF: First Multivalent Nuclear Factor	9	12,33	3,36E-08	PIK3CG, CDKN1B, PIK3CA, MDM2, MTOR, PTEN, MYC, PIK3R1, TGFB2	39	25	1625	15	5,08E-06	5,08E-06	4,02E-05
BIOCARTA	h_her2Pathway:Role of ERBB2 in Signal Transduction and Oncology	7	9,59	8,22E-06	PIK3CG, EGFR, IL6, EP300, ERBB2, PIK3CA, PIK3R1	39	23	1625	12,68	0,001	6,21E-04	0,0098
BIOCARTA	h_bcellsurvivalPathway:B Cell Survival Pathway	5	6,85	2,89E-04	PIK3CG, CASP3, PIK3CA, MTOR, PIK3R1	39	15	1625	13,89	0,043	0,0144	0,3450

BIOCARTA	h_il7Pathway:IL-7 Signal Transduction	5	6,85	4,87E-04	PIK3CG, EP300, BCL2, PIK3CA, PIK3R1	39	17	1625	12,25	0,071	0,0182	0,5809
BIOCARTA	h_arfPathway:Tumor Suppressor Arf Inhibits Ribosomal Biogenesis	5	6,85	6,16E-04	PIK3CG, PIK3CA, MDM2, MYC, PIK3R1	39	18	1625	11,57	0,089	0,0184	0,7339
BIOCARTA	h_gcrpathway:Corticosteroids and cardioprotection	5	6,85	7,67E-04	PIK3CG, PIK3CA, NFKB1, NR3C1, PIK3R1	39	19	1625	10,96	0,109	0,0191	0,9133
BIOCARTA	h_metPathway:Signaling of Hepatocyte Growth Factor Receptor	6	8,22	0,0012	PIK3CG, MET, PIK3CA, ELK1, PTEN, PIK3R1	39	36	1625	6,94	0,164	0,0253	1,4086
BIOCARTA	h_rasPathway:Ras Signaling Pathway	5	6,85	0,0016	PIK3CG, PIK3CA, ELK1, NFKB1, PIK3R1	39	23	1625	9,06	0,219	0,0305	1,9411
BIOCARTA	h_nthiPathway:NFkB activation by Nontypeable Hemophilus influenzae	5	6,85	0,0019	EP300, DUSP1, CXCL8, NFKB1, NR3C1	39	24	1625	8,68	0,253	0,0319	2,2867
BIOCARTA	h_tffPathway:Trefoil Factors Initiate Mucosal Healing	5	6,85	0,0026	PIK3CG, EGFR, ERBB2, PIK3CA, PIK3R1	39	26	1625	8,01	0,328	0,0390	3,0989
BIOCARTA	h_raccycdPathway:Influence of Ras and Rho proteins on G1 to S Transition	5	6,85	0,0030	CCND1, CDKN1B, PIK3CA, NFKB1, PIK3R1	39	27	1625	7,72	0,368	0,0409	3,5692
BIOCARTA	h_PparaPathway:Mechanism of Gene Regulation by Peroxisome Proliferators via PPARa(alpha)	6	8,22	0,0062	PIK3CG, EP300, DUSP1, PIK3CA, MYC, PIK3R1	39	52	1625	4,81	0,610	0,0755	7,1853
BIOCARTA	h_deathPathway:Induction of apoptosis through DR3 and DR4/5 Death Receptors	5	6,85	0,0064	CASP3, TNFSF10, BCL2, LMNA, NFKB1	39	33	1625	6,31	0,621	0,0718	7,3869

**BIOCARTA**	h_hcmvPathway:Human Cytomegalovirus and Map Kinase Pathways	4	5,48	0,0064	PIK3CG, PIK3CA, NFKB1, PIK3R1	39	17	1625	9,80	0,621	0,0669	7,3899
**BIOCARTA**	h_telPathway:Telomeres, Telomerase, Cellular Aging, and Immortality	4	5,48	0,0076	EGFR, KRAS, BCL2, MYC	39	18	1625	9,26	0,682	0,0735	8,6710
**BIOCARTA**	h_ptenPathway:PTEN dependent cell cycle arrest and apoptosis	4	5,48	0,0076	CDKN1B, PIK3CA, PTEN, PIK3R1	39	18	1625	9,26	0,682	0,0735	8,6710
**BIOCARTA**	h_ngfPathway:Nerve growth factor pathway (NGF)	4	5,48	0,0102	PIK3CG, PIK3CA, ELK1, PIK3R1	39	20	1625	8,33	0,788	0,0924	11,5606
**BIOCARTA**	h_igf1mtorPathway:Skeletal muscle hypertrophy is regulated via AKT/mTOR pathway	4	5,48	0,0102	PIK3CA, MTOR, PTEN, PIK3R1	39	20	1625	8,33	0,788	0,0924	11,5606
**BIOCARTA**	h_il2rbPathway:IL-2 Receptor Beta Chain in T cell Activation	5	6,85	0,0116	PIK3CG, BCL2, PIK3CA, MYC, PIK3R1	39	39	1625	5,34	0,829	0,0987	13,0506
**BIOCARTA**	h_fcer1Pathway:Fc Epsilon Receptor I Signaling in Mast Cells	5	6,85	0,0116	PIK3CG, PIK3CA, ELK1, MAP2K7, PIK3R1	39	39	1625	5,34	0,829	0,0987	13,0506
**BIOCARTA**	h_insulinPathway:Insulin Signaling Pathway	4	5,48	0,0117	PIK3CG, PIK3CA, ELK1, PIK3R1	39	21	1625	7,94	0,832	0,0943	13,1634
**BIOCARTA**	h_igf1Pathway:IGF-1 Signaling Pathway	4	5,48	0,0117	PIK3CG, PIK3CA, ELK1, PIK3R1	39	21	1625	7,94	0,832	0,0943	13,1634
**BIOCARTA**	h_hivnefPathway:HIV-I Nef: negative effector of Fas and TNF	6	8,22	0,0122	CASP3, BCL2, LMNA, MDM2, NFKB1, MAP2K7	39	61	1625	4,10	0,843	0,0930	13,6528

**BIOCARTA**	h_aktPathway:AKT Signaling Pathway	4	5,48	0,0134	PIK3CG, PIK3CA, NFKB1, PIK3R1	39	22	1625	7,58	0,869	0,0966	14,8663
**BIOCARTA**	h_gleevecPathway:Inhibition of Cellular Proliferation by Gleevec	4	5,48	0,0151	PIK3CG, PIK3CA, MYC, PIK3R1	39	23	1625	7,25	0,900	0,1038	16,6644
**BIOCARTA**	h_eif4Pathway:Regulation of eIF4e and p70 S6 Kinase	4	5,48	0,0170	PIK3CA, MTOR, PTEN, PIK3R1	39	24	1625	6,94	0,925	0,1111	18,5520
**BIOCARTA**	h_plcPathway:Phospholipase C Signaling Pathway	3	4,11	0,0173	PIK3CG, PIK3CA, PIK3R1	39	9	1625	13,89	0,928	0,1082	18,8196
**BIOCARTA**	h_tcrPathway:T Cell Receptor Signaling Pathway	5	6,85	0,0177	PIK3CG, PIK3CA, ELK1, NFKB1, PIK3R1	39	44	1625	4,73	0,932	0,1060	19,1773
**BIOCARTA**	h_mtorPathway:mTOR Signaling Pathway	4	5,48	0,0212	PIK3CA, MTOR, PTEN, PIK3R1	39	26	1625	6,41	0,960	0,1212	22,5704
**BIOCARTA**	h_badPathway:Regulation of BAD phosphorylation	4	5,48	0,0212	PIK3CG, BCL2, PIK3CA, PIK3R1	39	26	1625	6,41	0,960	0,1212	22,5704
**BIOCARTA**	h_egfPathway:EGF Signaling Pathway	4	5,48	0,0234	EGFR, PIK3CA, ELK1, PIK3R1	39	27	1625	6,17	0,972	0,1287	24,6870
**BIOCARTA**	H_gsk3Pathway:Inactivation of Gsk3 by AKT causes accumulation of b-catenin in Alveolar Macrophages	4	5,48	0,0234	CCND1, PIK3CA, NFKB1, PIK3R1	39	27	1625	6,17	0,972	0,1287	24,6870
**BIOCARTA**	h_keratinocytePathway:Keratinocyte Differentiation	5	6,85	0,0237	CEBPA, EGFR, BCL2, NFKB1, MAP2K7	39	48	1625	4,34	0,973	0,1254	24,8971

**BIOCARTA**	h_pdgfPathway:PDGF Signaling Pathway	4	5,48	0,0258	PIK3CG, PIK3CA, ELK1, PIK3R1	39	28	1625	5,95	0,981	0,1316	26,8653
**BIOCARTA**	h_vegfPathway:VEGF, Hypoxia, and Angiogenesis	4	5,48	0,0338	PIK3CG, HIF1A, PIK3CA, PIK3R1	39	31	1625	5,38	0,994	0,1638	33,6886
**BIOCARTA**	h_longevityPathway:The IGF-1 Receptor and Longevity	3	4,11	0,0462	PIK3CG, PIK3CA, PIK3R1	39	15	1625	8,33	0,999	0,2117	43,1715
**BIOCARTA**	h_mapkPathway:MAPKinase Signaling Pathway	6	8,22	0,0487	CEBPA, ELK1, NFKB1, MAP2K7, MYC, TGFB2	39	87	1625	2,87	0,999	0,2157	44,9203
**BIOCARTA**	h_achPathway:Role of nicotinic acetylcholine receptors in the regulation of apoptosis	3	4,11	0,0520	PIK3CG, PIK3CA, PIK3R1	39	16	1625	7,81	0,999	0,2227	47,1882
**BIOCARTA**	h_p53Pathway:p53 Signaling Pathway	3	4,11	0,0581	CCND1, BCL2, MDM2	39	17	1625	7,35	0,999	0,2395	51,0983
**BIOCARTA**	h_p38mapkPathway:p38 MAPK Signaling Pathway	4	5,48	0,0643	ELK1, MYC, DDIT3, TGFB2	39	40	1625	4,17	0,999	0,2557	54,8331
**BIOCARTA**	h_cardiacEGFPathway:Role of EGF Receptor Transactivation by GPCRs in Cardiac Hypertrophy	3	4,11	0,0644	EGFR, NFKB1, MYC	39	18	1625	6,94	0,999	0,2496	54,8773
**BIOCARTA**	h_erk5Pathway:Role of Erk5 in Neuronal Survival	3	4,11	0,0644	PIK3CG, PIK3CA, PIK3R1	39	18	1625	6,94	0,999	0,2496	54,8773
**BIOCARTA**	h_par1pathway:Thrombin signaling and protease-activated receptors	3	4,11	0,0990	PIK3CG, PIK3CA, PIK3R1	39	23	1625	5,43	0,999	0,3543	71,2604

**BIOCARTA**	h_rac1Pathway:Rac 1 cell motility signaling pathway	3	4,11	0,0990	PIK3CG, PIK3CA, PIK3R1	39	23	1625	5,43	0,999	0,3543	71,2604

**Category**	**Term**	**Count**	**%**	**PValue**	**Genes**	**List Total**	**Pop Hits**	**Pop Total**	**Fold Enrichment**	**Bonferroni**	**Benjamini**	**FDR**
**KEGG_PATHWAY**	hsa05200:Pathways in cancer	29	39,73	3,42E-22	PTGS2, ERBB2, CXCL8, MLH1, NFKB1, PTEN, TGFB2, CASP3, KRAS, BCL2, PIK3CA, MYC, PIK3R1, EGFR, PIK3CG, CEBPA, AR, IL6, PIK3CB, MET, DAPK1, CCND1, CDKN1B, HIF1A, EP300, RASSF1, MDM2, PTCH1, MTOR	52	393	6879	9,76	5,44E-20	5,44E-20	4,13E-19
**KEGG_PATHWAY**	hsa05215:Prostate cancer	16	21,92	2,50E-17	PIK3CG, EGFR, AR, PIK3CB, ERBB2, NFKB1, PTEN, CCND1, EP300, KRAS, CDKN1B, BCL2, MDM2, PIK3CA, MTOR, PIK3R1	52	88	6879	24,05	3,97E-15	1,99E-15	3,01E-14
**KEGG_PATHWAY**	hsa05205:Proteoglycans in cancer	20	27,40	5,58E-17	EGFR, PIK3CG, PIK3CB, ERBB2, MET, ELK1, IGF2, TGFB2, CASP3, CCND1, SDC1, HIF1A, KRAS, MDM2, PIK3CA, PTCH1, MTOR, THBS1, MYC, PIK3R1	52	200	6879	13,23	1,77E-14	5,88E-15	1,33E-13
**KEGG_PATHWAY**	hsa05161:Hepatitis B	17	23,29	2,53E-15	PIK3CG, IL6, PIK3CB, CXCL8, ELK1, NFKB1, PTEN, TGFB2, CASP3, CCND1, EP300, KRAS, CDKN1B, BCL2, PIK3CA, MYC, PIK3R1	52	145	6879	15,51	4,06E-13	1,01E-13	3,08E-12

**KEGG_PATHWAY**	hsa05206:MicroRNAs in cancer	21	28,77	3,04E-15	EGFR, PTGS2, ERBB2, EZH2, MET, NFKB1, PTEN, TGFB2, CASP3, CCND1, EP300, KRAS, CDKN1B, RASSF1, BCL2, DNMT1, MDM2, STMN1, MTOR, THBS1, MYC	52	286	6879	9,71	4,77E-13	9,54E-14	3,62E-12
**KEGG_PATHWAY**	hsa05213:Endometrial cancer	12	16,44	5,69E-14	PIK3CG, EGFR, CCND1, KRAS, PIK3CB, ERBB2, PIK3CA, MLH1, ELK1, PTEN, MYC, PIK3R1	52	52	6879	30,53	9,04E-12	1,51E-12	6,86E-11
**KEGG_PATHWAY**	hsa05230:Central carbon metabolism in cancer	12	16,44	6,57E-13	PIK3CG, EGFR, KRAS, HIF1A, PIK3CB, ERBB2, MET, PIK3CA, MTOR, PTEN, MYC, PIK3R1	52	64	6879	24,80	1,04E-10	1,49E-11	7,92E-10
**KEGG_PATHWAY**	hsa04066:HIF-1 signaling pathway	13	17,81	2,74E-12	EGFR, PIK3CG, IL6, CDKN1B, HIF1A, EP300, PIK3CB, BCL2, ERBB2, PIK3CA, NFKB1, MTOR, PIK3R1	52	96	6879	17,91	4,36E-10	5,45E-11	3,31E-09
**KEGG_PATHWAY**	hsa04068:FoxO signaling pathway	14	19,18	8,20E-12	PIK3CG, EGFR, IL6, PIK3CB, PTEN, TGFB2, CCND1, TNFSF10, EP300, KRAS, CDKN1B, MDM2, PIK3CA, PIK3R1	52	134	6879	13,82	1,30E-09	1,45E-10	9,89E-09
**KEGG_PATHWAY**	hsa05219:Bladder cancer	10	13,70	9,45E-12	EGFR, CCND1, KRAS, RASSF1, ERBB2, CXCL8, MDM2, THBS1, MYC, DAPK1	52	41	6879	32,27	1,50E-09	1,50E-10	1,14E-08

**KEGG_PATHWAY**	hsa05210:Colorectal cancer	11	15,07	1,59E-11	PIK3CG, CASP3, CCND1, KRAS, PIK3CB, BCL2, PIK3CA, MLH1, MYC, PIK3R1, TGFB2	52	62	6879	23,47	2,53E-09	2,30E-10	1,92E-08
**KEGG_PATHWAY**	hsa05222:Small cell lung cancer	12	16,44	1,68E-11	PIK3CG, FHIT, CCND1, CDKN1B, PTGS2, PIK3CB, BCL2, PIK3CA, NFKB1, PTEN, MYC, PIK3R1	52	85	6879	18,68	2,68E-09	2,23E-10	2,03E-08
**KEGG_PATHWAY**	hsa04012:ErbB signaling pathway	12	16,44	2,19E-11	PIK3CG, EGFR, CDKN1B, KRAS, PIK3CB, ERBB2, PIK3CA, ELK1, MTOR, MAP2K7, MYC, PIK3R1	52	87	6879	18,25	3,48E-09	2,67E-10	2,64E-08
**KEGG_PATHWAY**	hsa05220:Chronic myeloid leukemia	11	15,07	7,52E-11	PIK3CG, CCND1, CDKN1B, KRAS, PIK3CB, PIK3CA, MDM2, NFKB1, MYC, PIK3R1, TGFB2	52	72	6879	20,21	1,20E-08	8,54E-10	9,07E-08
**KEGG_PATHWAY**	hsa04151:PI3K-Akt signaling pathway	18	24,66	1,69E-10	EGFR, PIK3CG, IL6, EFNA1, PIK3CB, MET, NFKB1, PTEN, CCND1, KRAS, CDKN1B, BCL2, MDM2, PIK3CA, MTOR, THBS1, MYC, PIK3R1	52	345	6879	6,90	2,68E-08	1,79E-09	2,03E-07
**KEGG_PATHWAY**	hsa05221:Acute myeloid leukemia	10	13,70	1,88E-10	PIK3CG, CEBPA, CCND1, KRAS, PIK3CB, PIK3CA, NFKB1, MTOR, MYC, PIK3R1	52	56	6879	23,62	2,99E-08	1,87E-09	2,27E-07

**KEGG_PATHWAY**	hsa05223:Non-small cell lung cancer	10	13,70	1,88E-10	PIK3CG, EGFR, FHIT, CCND1, KRAS, PIK3CB, RASSF1, ERBB2, PIK3CA, PIK3R1	52	56	6879	23,62	2,99E-08	1,87E-09	2,27E-07
**KEGG_PATHWAY**	hsa05212:Pancreatic cancer	10	13,70	7,55E-10	PIK3CG, EGFR, CCND1, KRAS, PIK3CB, ERBB2, PIK3CA, NFKB1, PIK3R1, TGFB2	52	65	6879	20,35	1,20E-07	7,06E-09	9,10E-07
**KEGG_PATHWAY**	hsa05214:Glioma	10	13,70	7,55E-10	PIK3CG, EGFR, CCND1, KRAS, PIK3CB, PIK3CA, MDM2, MTOR, PTEN, PIK3R1	52	65	6879	20,35	1,20E-07	7,06E-09	9,10E-07
**KEGG_PATHWAY**	hsa05218:Melanoma	10	13,70	1,70E-09	PIK3CG, EGFR, CCND1, KRAS, PIK3CB, MET, PIK3CA, MDM2, PTEN, PIK3R1	52	71	6879	18,63	2,70E-07	1,50E-08	2,05E-06
**KEGG_PATHWAY**	hsa04919:Thyroid hormone signaling pathway	11	15,07	8,26E-09	PIK3CG, CCND1, KRAS, HIF1A, EP300, PIK3CB, PIK3CA, MDM2, MTOR, MYC, PIK3R1	52	115	6879	12,65	1,31E-06	6,91E-08	9,96E-06
**KEGG_PATHWAY**	hsa05211:Renal cell carcinoma	9	12,33	2,14E-08	PIK3CG, KRAS, HIF1A, EP300, PIK3CB, MET, PIK3CA, PIK3R1, TGFB2	52	66	6879	18,04	3,40E-06	1,70E-07	2,58E-05

**KEGG_PATHWAY**	hsa05169:Epstein-Barr virus infection	10	13,70	2,10E-07	PIK3CG, CDKN1B, PIK3CB, BCL2, PIK3CA, MDM2, NFKB1, MAP2K7, MYC, PIK3R1	52	122	6879	10,84	3,34E-05	1,59E-06	2,53E-04
**KEGG_PATHWAY**	hsa04510:Focal adhesion	12	16,44	2,22E-07	PIK3CG, EGFR, CCND1, PIK3CB, BCL2, ERBB2, MET, PIK3CA, ELK1, THBS1, PTEN, PIK3R1	52	206	6879	7,71	3,52E-05	1,60E-06	2,67E-04
**KEGG_PATHWAY**	hsa04210:Apoptosis	8	10,96	2,90E-07	PIK3CG, CASP3, TNFSF10, PIK3CB, BCL2, PIK3CA, NFKB1, PIK3R1	52	62	6879	17,07	4,61E-05	2,00E-06	3,50E-04
**KEGG_PATHWAY**	hsa05146:Amoebiasis	9	12,33	8,96E-07	PIK3CG, CASP3, IL6, PIK3CB, CXCL8, PIK3CA, NFKB1, PIK3R1, TGFB2	52	106	6879	11,23	1,42E-04	5,94E-06	0,0011
**KEGG_PATHWAY**	hsa04668:TNF signaling pathway	9	12,33	9,63E-07	PIK3CG, CASP3, IL6, PTGS2, PIK3CB, PIK3CA, NFKB1, MAP2K7, PIK3R1	52	107	6879	11,13	1,53E-04	6,13E-06	0,0012
**KEGG_PATHWAY**	hsa04932:Non-alcoholic fatty liver disease (NAFLD)	10	13,70	1,29E-06	PIK3CG, CEBPA, CASP3, IL6, PIK3CB, CXCL8, PIK3CA, NFKB1, PIK3R1, DDIT3	52	151	6879	8,76	2,05E-04	7,90E-06	0,0016

**KEGG_PATHWAY**	hsa05166:HTLV-I infection	12	16,44	1,80E-06	PIK3CG, IL6, CCND1, KRAS, EP300, PIK3CB, PIK3CA, ELK1, NFKB1, MYC, PIK3R1, TGFB2	52	254	6879	6,25	2,86E-04	1,06E-05	0,0022
**KEGG_PATHWAY**	hsa05203:Viral carcinogenesis	11	15,07	1,95E-06	PIK3CG, CASP3, CCND1, CDKN1B, KRAS, EP300, PIK3CB, PIK3CA, MDM2, NFKB1, PIK3R1	52	205	6879	7,10	3,10E-04	1,11E-05	0,0024
**KEGG_PATHWAY**	hsa05164:Influenza A	10	13,70	4,20E-06	PIK3CG, IL6, TNFSF10, EP300, PIK3CB, CXCL8, PIK3CA, NFKB1, MAP2K7, PIK3R1	52	174	6879	7,60	6,68E-04	2,30E-05	0,0051
**KEGG_PATHWAY**	hsa04014:Ras signaling pathway	11	15,07	4,70E-06	PIK3CG, EGFR, KRAS, PIK3CB, EFNA1, RASSF1, MET, PIK3CA, ELK1, NFKB1, PIK3R1	52	226	6879	6,44	7,47E-04	2,49E-05	0,0057
**KEGG_PATHWAY**	hsa05162:Measles	9	12,33	5,00E-06	PIK3CG, IL6, TNFSF10, CCND1, CDKN1B, PIK3CB, PIK3CA, NFKB1, PIK3R1	52	133	6879	8,95	7,95E-04	2,57E-05	0,0060
**KEGG_PATHWAY**	hsa05231:Choline metabolism in cancer	8	10,96	8,14E-06	PIK3CG, EGFR, KRAS, HIF1A, PIK3CB, PIK3CA, MTOR, PIK3R1	52	101	6879	10,48	0,001	4,04E-05	0,0098

**KEGG_PATHWAY**	hsa05142:Chagas disease (American trypanosomiasis)	8	10,96	9,88E-06	PIK3CG, IL6, PIK3CB, CXCL8, PIK3CA, NFKB1, PIK3R1, TGFB2	52	104	6879	10,18	0,002	4,76E-05	0,0119
**KEGG_PATHWAY**	hsa04620:Toll-like receptor signaling pathway	8	10,96	1,12E-05	PIK3CG, IL6, PIK3CB, CXCL8, PIK3CA, NFKB1, MAP2K7, PIK3R1	52	106	6879	9,98	0,002	5,24E-05	0,0135
**KEGG_PATHWAY**	hsa04917:Prolactin signaling pathway	7	9,59	1,22E-05	PIK3CG, CCND1, KRAS, PIK3CB, PIK3CA, NFKB1, PIK3R1	52	71	6879	13,04	0,002	5,53E-05	0,0147
**KEGG_PATHWAY**	hsa04931:Insulin resistance	8	10,96	1,27E-05	PIK3CG, IL6, PIK3CB, PIK3CA, NFKB1, MTOR, PTEN, PIK3R1	52	108	6879	9,80	0,002	5,60E-05	0,0153
**KEGG_PATHWAY**	hsa04010:MAPK signaling pathway	11	15,07	1,28E-05	EGFR, CASP3, KRAS, DUSP1, ELK1, NFKB1, STMN1, MAP2K7, MYC, DDIT3, TGFB2	52	253	6879	5,75	0,002	5,50E-05	0,0154
**KEGG_PATHWAY**	hsa04071:Sphingolipid signaling pathway	8	10,96	2,53E-05	PIK3CG, KRAS, PIK3CB, BCL2, PIK3CA, NFKB1, PTEN, PIK3R1	52	120	6879	8,82	0,004	1,06E-04	0,0305
**KEG G_P**	hsa04722:Neurotrophin signaling pathway	8	10,	2,53	PIK3CG, KRAS, PIK3CB, BCL2, PIK3CA, NFKB1, MAP2K7, PIK3R1	52	120	6879	8,82	0,004	1,06	0,0305

**ATHWAY**			96	E-05							E-04	
**KEGG_PATHWAY**	hsa05202:Transcriptional misregulation in cancer	9	12,33	2,67E-05	CEBPA, IL6, CDKN1B, MET, CXCL8, MDM2, NFKB1, MYC, DDIT3	52	167	6879	7,13	0,004	1,09E-04	0,0322
**KEGG_PATHWAY**	hsa05144:Malaria	6	8,22	2,73E-05	IL6, SDC1, MET, CXCL8, THBS1, TGFB2	52	49	6879	16,20	0,004	1,09E-04	0,0330
**KEGG_PATHWAY**	hsa04380:Osteoclast differentiation	8	10,96	4,46E-05	PIK3CG, CYBB, PIK3CB, PIK3CA, NFKB1, MAP2K7, PIK3R1, TGFB2	52	131	6879	8,08	0,007	1,73E-04	0,0538
**KEGG_PATHWAY**	hsa05160:Hepatitis C	8	10,96	4,91E-05	PIK3CG, EGFR, KRAS, PIK3CB, CXCL8, PIK3CA, NFKB1, PIK3R1	52	133	6879	7,96	0,008	1,86E-04	0,0593
**KEGG_PATHWAY**	hsa04150:mTOR signaling pathway	6	8,22	6,25E-05	PIK3CG, PIK3CB, PIK3CA, MTOR, PTEN, PIK3R1	52	58	6879	13,69	0,010	2,31E-04	0,0753
**KEGG_PATHWAY**	hsa04370:VEGF signaling pathway	6	8,22	7,97E-05	PIK3CG, KRAS, PTGS2, PIK3CB, PIK3CA, PIK3R1	52	61	6879	13,01	0,013	2,88E-04	0,0961

**KEGG_PATHWAY**	hsa04630:Jak-STAT signaling pathway	8	10,96	8,52E-05	PIK3CG, IL6, CCND1, EP300, PIK3CB, PIK3CA, MYC, PIK3R1	52	145	6879	7,30	0,0013	3,01E-04	0,1027
**KEGG_PATHWAY**	hsa04660:T cell receptor signaling pathway	7	9,59	8,61E-05	PIK3CG, KRAS, PIK3CB, PIK3CA, NFKB1, MAP2K7, PIK3R1	52	100	6879	9,26	0,0014	2,98E-04	0,1038
**KEGG_PATHWAY**	hsa04664:Fc epsilon RI signaling pathway	6	8,22	1,34E-04	PIK3CG, KRAS, PIK3CB, PIK3CA, MAP2K7, PIK3R1	52	68	6879	11,67	0,0021	4,55E-04	0,1620
**KEGG_PATHWAY**	hsa04015:Rap1 signaling pathway	9	12,33	1,36E-04	PIK3CG, EGFR, KRAS, PIK3CB, EFNA1, MET, PIK3CA, THBS1, PIK3R1	52	210	6879	5,67	0,0021	4,49E-04	0,1636
**KEGG_PATHWAY**	hsa04662:B cell receptor signaling pathway	6	8,22	1,44E-04	PIK3CG, KRAS, PIK3CB, PIK3CA, NFKB1, PIK3R1	52	69	6879	11,50	0,0023	4,67E-04	0,1736
**KEGG_PATHWAY**	hsa04960:Aldosterone-regulated sodium reabsorption	5	6,85	1,82E-04	PIK3CG, KRAS, PIK3CB, PIK3CA, PIK3R1	52	39	6879	16,96	0,0029	5,79E-04	0,2194
**KEGG_PATHWAY**	hsa04650:Natural killer cell mediated cytotoxicity	7	9,59	2,58E-04	PIK3CG, CASP3, TNFSF10, KRAS, PIK3CB, PIK3CA, PIK3R1	52	122	6879	7,59	0,0040	8,04E-04	0,3108

**KEGG_PATHWAY**	hsa04930:Type II diabetes mellitus	5	6,85	4,10E-04	PIK3CG, PIK3CB, PIK3CA, MTOR, PIK3R1	52	48	6879	13,78	0,063	0,0013	0,4935
**KEGG_PATHWAY**	hsa04910:Insulin signaling pathway	7	9,59	5,01E-04	PIK3CG, KRAS, PIK3CB, PIK3CA, ELK1, MTOR, PIK3R1	52	138	6879	6,71	0,077	0,0015	0,6029
**KEGG_PATHWAY**	hsa04923:Regulation of lipolysis in adipocytes	5	6,85	7,41E-04	PIK3CG, PTGS2, PIK3CB, PIK3CA, PIK3R1	52	56	6879	11,81	0,111	0,0022	0,8901
**KEGG_PATHWAY**	hsa04915:Estrogen signaling pathway	6	8,22	7,76E-04	PIK3CG, EGFR, KRAS, PIK3CB, PIK3CA, PIK3R1	52	99	6879	8,02	0,116	0,0022	0,9317
**KEGG_PATHWAY**	hsa04725:Cholinergic synapse	6	8,22	0,0013	PIK3CG, KRAS, PIK3CB, BCL2, PIK3CA, PIK3R1	52	111	6879	7,15	0,187	0,0037	1,5571
**KEGG_PATHWAY**	hsa04115:p53 signaling pathway	5	6,85	0,0015	CASP3, CCND1, MDM2, THBS1, PTEN	52	67	6879	9,87	0,207	0,0041	1,7425
**KEGG_PATHWAY**	hsa05120:Epithelial cell signaling in Helicobacter pylori infection	5	6,85	0,0015	EGFR, CASP3, MET, CXCL8, NFKB1	52	67	6879	9,87	0,207	0,0041	1,7425

**KEGG_PATHWAY**	hsa04152:AMPK signaling pathway	6	8,22	0,0021	PIK3CG, CCND1, PIK3CB, PIK3CA, MTOR, PIK3R1	52	123	6879	6,45	0,278	0,0056	2,4455
**KEGG_PATHWAY**	hsa04110:Cell cycle	6	8,22	0,0021	CCND1, CDKN1B, EP300, MDM2, MYC, TGFB2	52	124	6879	6,40	0,287	0,0057	2,5331
**KEGG_PATHWAY**	hsa04062:Chemokine signaling pathway	7	9,59	0,0024	PIK3CG, KRAS, PIK3CB, CXCL8, PIK3CA, NFKB1, PIK3R1	52	186	6879	4,98	0,313	0,0062	2,8110
**KEGG_PATHWAY**	hsa05100:Bacterial invasion of epithelial cells	5	6,85	0,0026	PIK3CG, PIK3CB, MET, PIK3CA, PIK3R1	52	78	6879	8,48	0,334	0,0066	3,0361
**KEGG_PATHWAY**	hsa04024:cAMP signaling pathway	7	9,59	0,0032	PIK3CG, EP300, PIK3CB, PIK3CA, NFKB1, PTCH1, PIK3R1	52	198	6879	4,68	0,402	0,0083	3,8245
**KEGG_PATHWAY**	hsa04973:Carbohydrate digestion and absorption	4	5,48	0,0036	PIK3CG, PIK3CB, PIK3CA, PIK3R1	52	42	6879	12,60	0,436	0,0091	4,2520
**KEGG_PATHWAY**	hsa04550:Signaling pathways regulating pluripotency of stem cells	6	8,22	0,0036	PIK3CG, KRAS, PIK3CB, PIK3CA, MYC, PIK3R1	52	140	6879	5,67	0,436	0,0089	4,2574

**KEGG_PATHWAY**	hsa04914:Progesterone-mediated oocyte maturation	5	6,85	0,0038	PIK3CG, KRAS, PIK3CB, PIK3CA, PIK3R1	52	87	6879	7,60	0,454	0,0093	4,4809
**KEGG_PATHWAY**	hsa04070:Phosphatidylinositol signaling system	5	6,85	0,0058	PIK3CG, PIK3CB, PIK3CA, PTEN, PIK3R1	52	98	6879	6,75	0,603	0,0139	6,7755
**KEGG_PATHWAY**	hsa05134:Legionellosis	4	5,48	0,0073	CASP3, IL6, CXCL8, NFKB1	52	54	6879	9,80	0,688	0,0172	8,4581
**KEGG_PATHWAY**	hsa05152:Tuberculosis	6	8,22	0,0096	CASP3, IL6, EP300, BCL2, NFKB1, TGFB2	52	177	6879	4,48	0,785	0,0223	11,0043
**KEGG_PATHWAY**	hsa04520:Adherens junction	4	5,48	0,0154	EGFR, EP300, ERBB2, MET	52	71	6879	7,45	0,915	0,0351	17,0775
**KEGG_PATHWAY**	hsa05140:Leishmaniasis	4	5,48	0,0154	PTGS2, ELK1, NFKB1, TGFB2	52	71	6879	7,45	0,915	0,0351	17,0775
**KEGG_PATHWAY**	hsa00562:Inositol phosphate metabolism	4	5,48	0,0154	PIK3CG, PIK3CB, PIK3CA, PTEN	52	71	6879	7,45	0,915	0,0351	17,0775

KEGG_PATHWAY	hsa05133:Pertussis	4	5,48	0,0178	CASP3, IL6, CXCL8, NFKB1	52	75	6879	7,06	0,943	0,0400	19,5063
KEGG_PATHWAY	hsa04810:Regulation of actin cytoskeleton	6	8,22	0,0190	PIK3CG, EGFR, KRAS, PIK3CB, PIK3CA, PIK3R1	52	210	6879	3,78	0,953	0,0421	20,6693
KEGG_PATHWAY	hsa05216:Thyroid cancer	3	4,11	0,0193	CCND1, KRAS, MYC	52	29	6879	13,69	0,955	0,0420	20,9075
KEGG_PATHWAY	hsa04350:TGF-beta signaling pathway	4	5,48	0,0240	EP300, THBS1, MYC, TGFB2	52	84	6879	6,30	0,979	0,0516	25,4170
KEGG_PATHWAY	hsa04666:Fc gamma R-mediated phagocytosis	4	5,48	0,0240	PIK3CG, PIK3CB, PIK3CA, PIK3R1	52	84	6879	6,30	0,979	0,0516	25,4170
KEGG_PATHWAY	hsa04921:Oxytocin signaling pathway	5	6,85	0,0246	EGFR, CCND1, KRAS, PTGS2, ELK1	52	150	6879	4,41	0,981	0,0520	25,9221
KEGG_PATHWAY	hsa05020:Prion diseases	3	4,11	0,0260	IL6, ELK1, HSPA5	52	34	6879	11,67	0,985	0,0543	27,2158
KEGG_P	hsa04064:NF-kappa B signaling pathway	4	5,	0,0263	PTGS2, BCL2, CXCL8, NFKB1	52	87	6879	6,08	0,986	0,0543	27,5

**ATHWAY**			48									019
**KEGG_PATHWAY**	hsa04912:GnRH signaling pathway	4	5,48	0,0295	EGFR, KRAS, ELK1, MAP2K7	52	91	6879	5,81	0,992	0,0600	30,3521
**KEGG_PATHWAY**	hsa04750:Inflammatory mediator regulation of TRP channels	4	5,48	0,0357	PIK3CG, PIK3CB, PIK3CA, PIK3R1	52	98	6879	5,40	0,997	0,0714	35,4859
**KEGG_PATHWAY**	hsa05145:Toxoplasmosis	4	5,48	0,0476	CASP3, BCL2, NFKB1, TGFB2	52	110	6879	4,81	0,999	0,0935	44,4874
**KEGG_PATHWAY**	hsa04670:Leukocyte transendothelial migration	4	5,48	0,0531	PIK3CG, PIK3CB, PIK3CA, PIK3R1	52	115	6879	4,60	0,999	0,1028	48,2256
**KEGG_PATHWAY**	hsa04621:NOD-like receptor signaling pathway	3	4,11	0,0644	IL6, CXCL8, NFKB1	52	56	6879	7,09	0,999	0,1225	55,1997
**KEGG_PATHWAY**	hsa04611:Platelet activation	4	5,48	0,0713	PIK3CG, PIK3CB, PIK3CA, PIK3R1	52	130	6879	4,07	0,999	0,1336	59,0316
**KEGG_PATHWAY**	hsa05321:Inflammatory bowel disease (IBD)	3	4,11	0,0812	IL6, NFKB1, TGFB2	52	64	6879	6,20	0,999	0,1498	64,0168

G. Signaling pathways in which the potential targets of miR-205-5p are involved (identified by DAVID)

Table 1: targets identified by BIOCARTA (DAVID tool)

Table 2: targets identified by KEGG (DAVID tool)

Category	Term	Count	%	PValue	Genes	List Total	Pop Hits	Pop Total	Fold Enrichment	Bonferroni	Benjamini	FDR
**BIOCARTA**	h_ctcfPathway:CTCF: First Multivalent Nuclear Factor	6	8,22	1,00E-04	PIK3CA, MDM2, RPS6KB1, PTEN, PIK3R1, TGFB2	34	25	1625	11,47	0,014	0,0142	0,1188
**BIOCARTA**	h_mtorPathway:mTOR Signaling Pathway	6	8,22	1,23E-04	EIF4E, TSC2, PIK3CA, RPS6KB1, PTEN, PIK3R1	34	26	1625	11,03	0,017	0,0087	0,1450
**BIOCARTA**	h_igf1mtorPathway:Skeletal muscle hypertrophy is regulated via AKT/mTOR pathway	5	6,85	5,44E-04	EIF4E, PIK3CA, RPS6KB1, PTEN, PIK3R1	34	20	1625	11,95	0,074	0,0254	0,6415
**BIOCARTA**	h_metPathway:Signaling of Hepatocyte Growth Factor Receptor	6	8,22	6,08E-04	PTK2B, RAF1, PIK3CA, ELK1, PTEN, PIK3R1	34	36	1625	7,97	0,083	0,0213	0,7165
**BIOCARTA**	h_fcer1Pathway:Fc Epsilon Receptor I Signaling in Mast Cells	6	8,22	8,88E-04	PLA2G4A, RAF1, PIK3CA, ELK1, MAP2K7, PIK3R1	34	39	1625	7,35	0,119	0,0249	1,0459
**BIOCARTA**	h_her2Pathway:Role of ERBB2 in Signal Transduction and Oncology	5	6,85	9,52E-04	EGFR, ERBB2, RAF1, PIK3CA, PIK3R1	34	23	1625	10,39	0,127	0,0223	1,1204

**BIOCARTA**	h_eif4Pathway:Regulation of eIF4e and p70 S6 Kinase	5	6,85	0,0011	EIF4E, PIK3CA, RPS6KB1, PTEN, PIK3R1	34	24	1625	9,96	0,148	0,0226	1,3241
**BIOCARTA**	h_raccycdPathway:Influence of Ras and Rho proteins on G1 to S Transition	5	6,85	0,0018	CDKN1A, CCND1, RAF1, PIK3CA, PIK3R1	34	27	1625	8,85	0,224	0,0312	2,0871
**BIOCARTA**	h_egfPathway:EGF Signaling Pathway	5	6,85	0,0018	EGFR, RAF1, PIK3CA, ELK1, PIK3R1	34	27	1625	8,85	0,224	0,0312	2,0871
**BIOCARTA**	h_il7Pathway:IL-7 Signal Transduction	4	5,48	0,0043	PTK2B, BCL2, PIK3CA, PIK3R1	34	17	1625	11,25	0,456	0,0654	4,9431
**BIOCARTA**	h_p53Pathway:p53 Signaling Pathway	4	5,48	0,0043	CDKN1A, CCND1, BCL2, MDM2	34	17	1625	11,25	0,456	0,0654	4,9431
**BIOCARTA**	h_hivnefPathway:HIV-I Nef: negative effector of Fas and TNF	6	8,22	0,0067	NUMA1, CASP3, BCL2, LMNA, MDM2, MAP2K7	34	61	1625	4,70	0,613	0,0906	7,6058
**BIOCARTA**	h_ngfPathway:Nerve growth factor pathway (NGF)	4	5,48	0,0069	RAF1, PIK3CA, ELK1, PIK3R1	34	20	1625	9,56	0,625	0,0852	7,8388
**BIOCARTA**	h_il2rbPathway:IL-2 Receptor Beta Chain in T cell Activation	5	6,85	0,0070	BCL2, RAF1, PIK3CA, RPS6KB1, PIK3R1	34	39	1625	6,13	0,633	0,0801	8,0038
**BIOCARTA**	h_igf1Pathway:IGF-1 Signaling Pathway	4	5,48	0,0079	RAF1, PIK3CA, ELK1, PIK3R1	34	21	1625	9,10	0,676	0,0831	8,9694
**BIOCARTA**	h_insulinPathway:Insulin Signaling Pathway	4	5,48	0,0079	RAF1, PIK3CA, ELK1, PIK3R1	34	21	1625	9,10	0,676	0,0831	8,9694

**BIOCARTA**	h_rasPathway:Ras Signaling Pathway	4	5,48	0,0102	RAF1, PIK3CA, ELK1, PIK3R1	34	23	1625	8,31	0,768	0,0992	11,4729
**BIOCARTA**	h_cxcr4Pathway:CXCR4 Signaling Pathway	4	5,48	0,0116	PTK2B, RAF1, PIK3CA, PIK3R1	34	24	1625	7,97	0,808	0,1042	12,8420
**BIOCARTA**	h_tffPathway:Trefoil Factors Initiate Mucosal Healing	4	5,48	0,0144	EGFR, ERBB2, PIK3CA, PIK3R1	34	26	1625	7,35	0,873	0,1211	15,8021
**BIOCARTA**	h_keratinocytePathway:Keratinocyte Differentiation	5	6,85	0,0146	CEBPA, EGFR, BCL2, RAF1, MAP2K7	34	48	1625	4,98	0,876	0,1157	15,9821
**BIOCARTA**	h_pdgfPathway:PDGF Signaling Pathway	4	5,48	0,0177	RAF1, PIK3CA, ELK1, PIK3R1	34	28	1625	6,83	0,921	0,1314	19,0362
**BIOCARTA**	h_g1Pathway:Cell Cycle: G1/S Check Point	4	5,48	0,0213	CDKN1A, CCND1, ATR, TGFB2	34	30	1625	6,37	0,953	0,1488	22,5128
**BIOCARTA**	h_at1rPathway:Angiotensin II mediated activation of JNK Pathway via Pyk2 dependent signaling	4	5,48	0,0275	EGFR, PTK2B, RAF1, ELK1	34	33	1625	5,79	0,981	0,1797	28,1024
**BIOCARTA**	h_mapkPathway:MAPKinase Signaling Pathway	6	8,22	0,0284	CEBPA, RAF1, ELK1, RPS6KB1, MAP2K7, TGFB2	34	87	1625	3,30	0,983	0,1768	28,8458
**BIOCARTA**	h_bcellsurvivalPathway:B Cell Survival Pathway	3	4,11	0,0356	CASP3, PIK3CA, PIK3R1	34	15	1625	9,56	0,994	0,2087	34,8771
**BIOCARTA**	h_achPathway:Role of nicotinic acetylcholine receptors in the regulation of apoptosis	3	4,11	0,0402	PTK2B, PIK3CA, PIK3R1	34	16	1625	8,96	0,997	0,2237	38,4405

**BIOCARTA**	h_arfPathway:Tumor Suppressor Arf Inhibits Ribosomal Biogenesis	3	4,11	0,0500	PIK3CA, MDM2, PIK3R1	34	18	1625	7,97	0,999	0,2616	45,4622
**BIOCARTA**	h_ptenPathway:PTEN dependent cell cycle arrest and apoptosis	3	4,11	0,0500	PIK3CA, PTEN, PIK3R1	34	18	1625	7,97	0,999	0,2616	45,4622
**BIOCARTA**	h_gcrpathway:Corticosteroids and cardioprotection	3	4,11	0,0551	PIK3CA, NR3C1, PIK3R1	34	19	1625	7,55	0,999	0,2754	48,8780
**BIOCARTA**	h_tcrPathway:T Cell Receptor Signaling Pathway	4	5,48	0,0574	RAF1, PIK3CA, ELK1, PIK3R1	34	44	1625	4,34	0,999	0,2760	50,3188
**BIOCARTA**	h_nkcellsPathway:Ras-Independent pathway in NK cell-mediated cytotoxicity	3	4,11	0,0605	PTK2B, PIK3CA, PIK3R1	34	20	1625	7,17	0,999	0,2798	52,2068
**BIOCARTA**	h_gleevecPathway:Inhibition of Cellular Proliferation by Gleevec	3	4,11	0,0776	RAF1, PIK3CA, PIK3R1	34	23	1625	6,23	0,999	0,3361	61,5374
**BIOCARTA**	h_rac1Pathway:Rac 1 cell motility signaling pathway	3	4,11	0,0776	PIK3CA, RPS6KB1, PIK3R1	34	23	1625	6,23	0,999	0,3361	61,5374
**BIOCARTA**	h_igf1rPathway:Multiple antiapoptotic pathways from IGF-1R signaling lead to BAD phosphorylation	3	4,11	0,0776	RAF1, PIK3CA, PIK3R1	34	23	1625	6,23	0,999	0,3361	61,5374
**BIOCARTA**	h_par1pathway:Thrombin signaling and protease-activated receptors	3	4,11	0,0776	PTK2B, PIK3CA, PIK3R1	34	23	1625	6,23	0,999	0,3361	61,5374
**BIOCARTA**	h_ecmPathway:Erk and PI-3 Kinase Are Necessary for Collagen Binding in Corneal Epithelia	3	4,11	0,0836	RAF1, PIK3CA, PIK3R1	34	24	1625	5,97	0,999	0,3479	64,3968

**BIOCARTA**	h_caspasePathway:Caspase Cascade in Apoptosis	3	4,11	0,0836	CASP3, CASP4, LMNA	34	24	1625	5,97	0,999	0,3479	64,3968
**BIOCARTA**	h_tpoPathway:TPO Signaling Pathway	3	4,11	0,0836	RAF1, PIK3CA, PIK3R1	34	24	1625	5,97	0,999	0,3479	64,3968
**BIOCARTA**	h_g2Pathway:Cell Cycle: G2/M Checkpoint	3	4,11	0,0898	CDKN1A, MDM2, ATR	34	25	1625	5,74	0,999	0,3592	67,1201
**BIOCARTA**	h_nfatPathway:NFAT and Hypertrophy of the heart (Transcription in the broken heart)	4	5,48	0,0939	RAF1, PIK3CA, RPS6KB1, PIK3R1	34	54	1625	3,54	0,999	0,3633	68,8348
**BIOCARTA**	h_badPathway:Regulation of BAD phosphorylation	3	4,11	0,0960	BCL2, PIK3CA, PIK3R1	34	26	1625	5,51	0,999	0,3611	69,7038

**Category**	**Term**	**Count**	**%**	**PValue**	**Genes**	**List Total**	**Pop Hits**	**Pop Total**	**Fold Enrichment**	**Bonferroni**	**Benjamini**	**FDR**
**KEGG_PATHWAY**	hsa05215:Prostate cancer	15	20,55	1,25E-15	FGFR2, EGFR, FGFR1, AR, PIK3CB, ERBB2, PIK3CD, RAF1, PTEN, CDKN1A, CCND1, BCL2, MDM2, PIK3CA, PIK3R1	53	88	6879	22,12	1,88E-13	1,88E-13	1,47E-12
**KEGG_PATHWAY**	hsa05205:Proteoglycans in cancer	19	26,	1,86E-15	EGFR, FGFR1, PIK3CB, ERBB2, PIK3CD, RAF1, ELK1, RPS6KB1, TGFB2, CASP3, CCND1, CDKN1A, CD44, VEGFA, MDM2, PIK3CA, PTCH1, THBS1, PIK3R1	53	200	6879	12,33	2,91E-13	1,45E-13	2,26E-12

			03									
**KEGG_PATHWAY**	hsa05200:Pathways in cancer	23	31,51	1,39E-14	CEBPA, EGFR, FGFR2, FGFR1, AR, MSH2, PIK3CB, ERBB2, PIK3CD, RAF1, PTEN, DAPK1, TGFB2, CASP3, CCND1, CDKN1A, RASSF1, BCL2, VEGFA, MDM2, PIK3CA, PTCH1, PIK3R1	53	393	6879	7,60	2,14E-12	7,12E-13	1,67E-11
**KEGG_PATHWAY**	hsa05219:Bladder cancer	10	13,70	1,14E-11	EGFR, CDKN1A, CCND1, RASSF1, ERBB2, VEGFA, RAF1, MDM2, THBS1, DAPK1	53	41	6879	31,66	1,75E-09	4,38E-10	1,36E-08
**KEGG_PATHWAY**	hsa04151:PI3K-Akt signaling pathway	19	26,03	2,33E-11	EGFR, FGFR2, FGFR1, EFNA1, PIK3CB, PIK3CD, RAF1, RPS6KB1, PTEN, CCND1, CDKN1A, EIF4E, BCL2, VEGFA, TSC2, MDM2, PIK3CA, THBS1, PIK3R1	53	345	6879	7,15	3,58E-09	7,17E-10	2,79E-08
**KEGG_PATHWAY**	hsa05218:Melanoma	11	15,07	8,00E-11	EGFR, FGFR1, CDKN1A, CCND1, PIK3CB, PIK3CD, RAF1, PIK3CA, MDM2, PTEN, PIK3R1	53	71	6879	20,11	1,23E-08	2,05E-09	9,59E-08
**KEGG_PATHWAY**	hsa05213:Endometrial cancer	10	13,70	1,12E-10	EGFR, CCND1, PIK3CB, ERBB2, PIK3CD, RAF1, PIK3CA, ELK1, PTEN, PIK3R1	53	52	6879	24,96	1,73E-08	2,47E-09	1,35E-07
**KEGG_PATHWAY**	hsa05223:Non-small cell lung cancer	10	13,	2,26E-10	EGFR, FHIT, CCND1, PIK3CB, RASSF1, ERBB2, PIK3CD, RAF1, PIK3CA, PIK3R1	53	56	6879	23,18	3,48E-08	4,35E-09	2,71E-07

			70									
**KEGG_PATHWAY**	hsa05161:Hepatitis B	13	17,81	4,88E-10	CDKN1A, CASP3, CCND1, PIK3CB, PTK2B, BCL2, PIK3CD, RAF1, PIK3CA, ELK1, PTEN, PIK3R1, TGFB2	53	145	6879	11,64	7,51E-08	8,35E-09	5,85E-07
**KEGG_PATHWAY**	hsa05210:Colorectal cancer	10	13,70	5,85E-10	CASP3, CCND1, PIK3CB, MSH2, BCL2, PIK3CD, RAF1, PIK3CA, PIK3R1, TGFB2	53	62	6879	20,93	9,01E-08	9,01E-09	7,01E-07
**KEGG_PATHWAY**	hsa04012:ErbB signaling pathway	11	15,07	6,33E-10	EGFR, CDKN1A, PIK3CB, ERBB2, PIK3CD, RAF1, PIK3CA, ELK1, RPS6KB1, MAP2K7, PIK3R1	53	87	6879	16,41	9,75E-08	8,87E-09	7,60E-07
**KEGG_PATHWAY**	hsa05230:Central carbon metabolism in cancer	10	13,70	7,85E-10	FGFR2, EGFR, FGFR1, PIK3CB, ERBB2, PIK3CD, RAF1, PIK3CA, PTEN, PIK3R1	53	64	6879	20,28	1,21E-07	1,01E-08	9,42E-07
**KEGG_PATHWAY**	hsa05214:Glioma	10	13,70	9,06E-10	EGFR, CDKN1A, CCND1, PIK3CB, PIK3CD, RAF1, PIK3CA, MDM2, PTEN, PIK3R1	53	65	6879	19,97	1,40E-07	1,07E-08	1,09E-06
**KEGG_PATHWAY**	hsa05212:Pancreatic cancer	10	13,70	9,06E-10	EGFR, CCND1, PIK3CB, ERBB2, PIK3CD, VEGFA, RAF1, PIK3CA, PIK3R1, TGFB2	53	65	6879	19,97	1,40E-07	1,07E-08	1,09E-06

**KEGG_PATHWAY**	hsa04066:HIF-1 signaling pathway	11	15,07	1,70E-09	EGFR, CDKN1A, EIF4E, PIK3CB, BCL2, ERBB2, PIK3CD, VEGFA, PIK3CA, RPS6KB1, PIK3R1	53	96	6879	14,87	2,61E-07	1,87E-08	2,04E-06
**KEGG_PATHWAY**	hsa05206:MicroRNAs in cancer	15	20,55	1,43E-08	EGFR, ERBB2, RAF1, PTEN, TGFB2, CDKN1A, CCND1, CASP3, CD44, RASSF1, BCL2, VEGFA, MDM2, DNMT1, THBS1	53	286	6879	6,81	2,20E-06	1,47E-07	1,72E-05
**KEGG_PATHWAY**	hsa04510:Focal adhesion	13	17,81	2,72E-08	EGFR, PIK3CB, ERBB2, PIK3CD, ELK1, RAF1, PTEN, CCND1, BCL2, VEGFA, PIK3CA, THBS1, PIK3R1	53	206	6879	8,19	4,19E-06	2,62E-07	3,26E-05
**KEGG_PATHWAY**	hsa04068:FoxO signaling pathway	11	15,07	4,43E-08	EGFR, CDKN1A, CCND1, PIK3CB, PIK3CD, RAF1, PIK3CA, MDM2, PTEN, PIK3R1, TGFB2	53	134	6879	10,65	6,83E-06	4,02E-07	5,32E-05
**KEGG_PATHWAY**	hsa05220:Chronic myeloid leukemia	9	12,33	5,04E-08	CDKN1A, CCND1, PIK3CB, PIK3CD, RAF1, PIK3CA, MDM2, PIK3R1, TGFB2	53	72	6879	16,22	7,77E-06	4,31E-07	6,05E-05
**KEGG_PATHWAY**	hsa04014:Ras signaling pathway	13	17,81	7,63E-08	EGFR, FGFR2, FGFR1, PLA2G4A, PIK3CB, EFNA1, RASSF1, PIK3CD, VEGFA, RAF1, PIK3CA, ELK1, PIK3R1	53	226	6879	7,47	1,17E-05	6,18E-07	9,15E-05

**KEGG_PATHWAY**	hsa05221:Acute myeloid leukemia	8	10,96	1,62E-07	CEBPA, CCND1, PIK3CB, PIK3CD, RAF1, PIK3CA, RPS6KB1, PIK3R1	53	56	6879	18,54	2,50E-05	1,25E-06	1,95E-04
**KEGG_PATHWAY**	hsa04150:mTOR signaling pathway	8	10,96	2,08E-07	EIF4E, PIK3CB, PIK3CD, TSC2, PIK3CA, RPS6KB1, PTEN, PIK3R1	53	58	6879	17,90	3,20E-05	1,53E-06	2,50E-04
**KEGG_PATHWAY**	hsa04115:p53 signaling pathway	8	10,96	5,71E-07	CDKN1A, CASP3, CCND1, TSC2, MDM2, ATR, THBS1, PTEN	53	67	6879	15,50	8,80E-05	4,00E-06	6,85E-04
**KEGG_PATHWAY**	hsa05231:Choline metabolism in cancer	9	12,33	7,21E-07	EGFR, PLA2G4A, PIK3CB, PIK3CD, TSC2, RAF1, PIK3CA, RPS6KB1, PIK3R1	53	101	6879	11,57	1,11E-04	4,83E-06	8,65E-04
**KEGG_PATHWAY**	hsa05222:Small cell lung cancer	8	10,96	2,92E-06	FHIT, CCND1, PIK3CB, BCL2, PIK3CD, PIK3CA, PTEN, PIK3R1	53	85	6879	12,22	4,50E-04	1,88E-05	0,0035
**KEGG_PATHWAY**	hsa04015:Rap1 signaling pathway	11	15,07	2,93E-06	FGFR2, EGFR, FGFR1, PIK3CB, EFNA1, PIK3CD, VEGFA, RAF1, PIK3CA, THBS1, PIK3R1	53	210	6879	6,80	4,50E-04	1,80E-05	0,0035

**KEGG_PATHWAY**	hsa05169:Epstein-Barr virus infection	9	12,33	3,05E-06	CDKN1A, CD44, PIK3CB, BCL2, PIK3CD, PIK3CA, MDM2, MAP2K7, PIK3R1	53	122	6879	9,57	4,69E-04	1,80E-05	0,0037
**KEGG_PATHWAY**	hsa04370:VEGF signaling pathway	7	9,59	5,61E-06	PLA2G4A, PIK3CB, PIK3CD, VEGFA, RAF1, PIK3CA, PIK3R1	53	61	6879	14,89	8,64E-04	3,20E-05	0,0067
**KEGG_PATHWAY**	hsa04910:Insulin signaling pathway	9	12,33	7,65E-06	EIF4E, PIK3CB, PIK3CD, TSC2, RAF1, PIK3CA, ELK1, RPS6KB1, PIK3R1	53	138	6879	8,46	0,001	4,21E-05	0,0092
**KEGG_PATHWAY**	hsa05211:Renal cell carcinoma	7	9,59	8,92E-06	PIK3CB, PIK3CD, VEGFA, RAF1, PIK3CA, PIK3R1, TGFB2	53	66	6879	13,77	0,001	4,74E-05	0,0107
**KEGG_PATHWAY**	hsa04664:Fc epsilon RI signaling pathway	7	9,59	1,06E-05	PLA2G4A, PIK3CB, PIK3CD, RAF1, PIK3CA, MAP2K7, PIK3R1	53	68	6879	13,36	0,002	5,45E-05	0,0127
**KEGG_PATHWAY**	hsa04668:TNF signaling pathway	8	10,96	1,36E-05	CASP3, CCL20, PIK3CB, PIK3CD, CXCL2, PIK3CA, MAP2K7, PIK3R1	53	107	6879	9,70	0,002	6,76E-05	0,0163
**KEGG_PATHWAY**	hsa04919:Thyroid hormone signaling pathway	8	10,	2,18E-05	CCND1, PIK3CB, PIK3CD, TSC2, RAF1, PIK3CA, MDM2, PIK3R1	53	115	6879	9,03	0,003	1,05E-04	0,0262

			96									
**KEGG_PATHWAY**	hsa04210:Apoptosis	6	8,22	9,48E-05	CASP3, PIK3CB, BCL2, PIK3CD, PIK3CA, PIK3R1	53	62	6879	12,56	0,014	4,42E-04	0,1136
**KEGG_PATHWAY**	hsa05166:HTLV-I infection	10	13,70	1,01E-04	CDKN1A, CCND1, MAD2L1, PIK3CB, PIK3CD, PIK3CA, ELK1, ATR, PIK3R1, TGFB2	53	254	6879	5,11	0,015	4,58E-04	0,1214
**KEGG_PATHWAY**	hsa04810:Regulation of actin cytoskeleton	9	12,33	1,56E-04	FGFR2, EGFR, FGFR1, PIK3CB, PIK3CD, RAF1, PIK3CA, TMSB4X, PIK3R1	53	210	6879	5,56	0,024	6,87E-04	0,1873
**KEGG_PATHWAY**	hsa04917:Prolactin signaling pathway	6	8,22	1,81E-04	CCND1, PIK3CB, PIK3CD, RAF1, PIK3CA, PIK3R1	53	71	6879	10,97	0,028	7,75E-04	0,2171
**KEGG_PATHWAY**	hsa04722:Neurotrophin signaling pathway	7	9,59	2,63E-04	PIK3CB, BCL2, PIK3CD, RAF1, PIK3CA, MAP2K7, PIK3R1	53	120	6879	7,57	0,040	0,0011	0,3148
**KEGG_PATHWAY**	hsa04071:Sphingolipid signaling pathway	7	9,59	2,63E-04	PIK3CB, BCL2, PIK3CD, RAF1, PIK3CA, PTEN, PIK3R1	53	120	6879	7,57	0,040	0,0011	0,3148
**KEGG_PATHWAY**	hsa04650:Natural killer cell mediated cytotoxicity	7	9,59	2,88E-04	CASP3, PIK3CB, PTK2B, PIK3CD, RAF1, PIK3CA, PIK3R1	53	122	6879	7,45	0,043	0,0012	0,3443

**KEGG_PATHWAY**	hsa04152:AMPK signaling pathway	7	9,59	3,01E-04	CCND1, PIK3CB, PIK3CD, TSC2, PIK3CA, RPS6KB1, PIK3R1	53	123	6879	7,39	0,045	0,012	0,3599
**KEGG_PATHWAY**	hsa04666:Fc gamma R-mediated phagocytosis	6	8,22	3,99E-04	PIK3CB, PIK3CD, RAF1, PIK3CA, RPS6KB1, PIK3R1	53	84	6879	9,27	0,060	0,015	0,4775
**KEGG_PATHWAY**	hsa04380:Osteoclast differentiation	7	9,59	4,22E-04	CYBB, PIK3CB, PIK3CD, PIK3CA, MAP2K7, PIK3R1, TGFB2	53	131	6879	6,94	0,063	0,016	0,5052
**KEGG_PATHWAY**	hsa04062:Chemokine signaling pathway	8	10,96	4,51E-04	CCL20, PIK3CB, PTK2B, PIK3CD, CXCL2, RAF1, PIK3CA, PIK3R1	53	186	6879	5,58	0,067	0,017	0,5396
**KEGG_PATHWAY**	hsa05160:Hepatitis C	7	9,59	4,58E-04	EGFR, CDKN1A, PIK3CB, PIK3CD, RAF1, PIK3CA, PIK3R1	53	133	6879	6,83	0,068	0,016	0,5478
**KEGG_PATHWAY**	hsa04914:Progesterone-mediated oocyte maturation	6	8,22	4,69E-04	MAD2L1, PIK3CB, PIK3CD, RAF1, PIK3CA, PIK3R1	53	87	6879	8,95	0,070	0,016	0,5616
**KEGG_PATHWAY**	hsa04010:MAPK signaling pathway	9	12,33	5,54E-04	FGFR2, EGFR, FGFR1, PLA2G4A, CASP3, RAF1, ELK1, MAP2K7, TGFB2	53	253	6879	4,62	0,082	0,019	0,6622

**KEGG_PATHWAY**	hsa04912:GnRH signaling pathway	6	8,22	5,77E-04	EGFR, PLA2G4A, PTK2B, RAF1, ELK1, MAP2K7	53	91	6879	8,56	0,085	0,0019	0,6905
**KEGG_PATHWAY**	hsa04550:Signaling pathways regulating pluripotency of stem cells	7	9,59	6,02E-04	FGFR2, FGFR1, PIK3CB, PIK3CD, RAF1, PIK3CA, PIK3R1	53	140	6879	6,49	0,089	0,020	0,7194
**KEGG_PATHWAY**	hsa05203:Viral carcinogenesis	8	10,96	8,07E-04	CDKN1A, CASP3, CCND1, PIK3CB, PIK3CD, PIK3CA, MDM2, PIK3R1	53	205	6879	5,07	0,117	0,026	0,9637
**KEGG_PATHWAY**	hsa04915:Estrogen signaling pathway	6	8,22	8,49E-04	EGFR, PIK3CB, PIK3CD, RAF1, PIK3CA, PIK3R1	53	99	6879	7,87	0,123	0,027	1,0132
**KEGG_PATHWAY**	hsa04660:T cell receptor signaling pathway	6	8,22	8,88E-04	PIK3CB, PIK3CD, RAF1, PIK3CA, MAP2K7, PIK3R1	53	100	6879	7,79	0,128	0,027	1,0602
**KEGG_PATHWAY**	hsa05146:Amoebiasis	6	8,22	0,00116	CASP3, PIK3CB, PIK3CD, PIK3CA, PIK3R1, TGFB2	53	106	6879	7,35	0,163	0,035	1,3773
**KEGG_PATHWAY**	hsa04931:Insulin resistance	6	8,22	0,00126	PIK3CB, PIK3CD, PIK3CA, RPS6KB1, PTEN, PIK3R1	53	108	6879	7,21	0,176	0,037	1,4970

**KEGG_PATHWAY**	hsa04662:B cell receptor signaling pathway	5	6,855	0,00175	PIK3CB, PIK3CD, RAF1, PIK3CA, PIK3R1	53	69	6879	9,41	0,236	0,0051	2,0757
**KEGG_PATHWAY**	hsa04110:Cell cycle	6	8,222	0,00232	CDKN1A, CCND1, MAD2L1, MDM2, ATR, TGFB2	53	124	6879	6,28	0,300	0,0066	2,7449
**KEGG_PATHWAY**	hsa04960:Aldosterone-regulated sodium reabsorption	4	5,448	0,00307	PIK3CB, PIK3CD, PIK3CA, PIK3R1	53	39	6879	13,31	0,378	0,0086	3,6253
**KEGG_PATHWAY**	hsa04973:Carbohydrate digestion and absorption	4	5,448	0,00380	PIK3CB, PIK3CD, PIK3CA, PIK3R1	53	42	6879	12,36	0,444	0,0104	4,4642
**KEGG_PATHWAY**	hsa04921:Oxytocin signaling pathway	6	8,222	0,00525	EGFR, PLA2G4A, CDKN1A, CCND1, RAF1, ELK1	53	150	6879	5,19	0,556	0,0141	6,1246
**KEGG_PATHWAY**	hsa04932:Non-alcoholic fatty liver disease (NAFLD)	6	8,222	0,00540	CEBPA, CASP3, PIK3CB, PIK3CD, PIK3CA, PIK3R1	53	151	6879	5,16	0,566	0,0143	6,2934
**KEGG_PATHWAY**	hsa04930:Type II diabetes mellitus	4	5,448	0,00555	PIK3CB, PIK3CD, PIK3CA, PIK3R1	53	48	6879	10,82	0,575	0,0144	6,4534

**KEGG_PATHWAY**	hsa04750:Inflammatory mediator regulation of TRP channels	5	6,85	0,006222	PLA2G4A, PIK3CB, PIK3CD, PIK3CA, PIK3R1	53	98	6879	6,62	0,617	0,0159	7,2066
**KEGG_PATHWAY**	hsa04070:Phosphatidylinositol signaling system	5	6,85	0,006222	PIK3CB, PIK3CD, PIK3CA, PTEN, PIK3R1	53	98	6879	6,62	0,617	0,0159	7,2066
**KEGG_PATHWAY**	hsa05142:Chagas disease (American trypanosomiasis)	5	6,85	0,007765	PIK3CB, PIK3CD, PIK3CA, PIK3R1, TGFB2	53	104	6879	6,24	0,694	0,0192	8,8047
**KEGG_PATHWAY**	hsa04620:Toll-like receptor signaling pathway	5	6,85	0,008118	PIK3CB, PIK3CD, PIK3CA, MAP2K7, PIK3R1	53	106	6879	6,12	0,718	0,0202	9,3810
**KEGG_PATHWAY**	hsa04923:Regulation of lipolysis in adipocytes	4	5,48	0,008852	PIK3CB, PIK3CD, PIK3CA, PIK3R1	53	56	6879	9,27	0,732	0,0207	9,7545
**KEGG_PATHWAY**	hsa04725:Cholinergic synapse	5	6,85	0,009559	PIK3CB, BCL2, PIK3CD, PIK3CA, PIK3R1	53	111	6879	5,85	0,773	0,0229	10,9174
**KEGG_PATHWAY**	hsa05164:Influenza A	6	8,22	0,009973	PIK3CB, PIK3CD, RAF1, PIK3CA, MAP2K7, PIK3R1	53	174	6879	4,48	0,778	0,0229	11,0629
**KEGG_PA**	hsa04670:Leukocyte transendothelial migration	5	6,	0,01	PIK3CB, PTK2B, PIK3CD, PIK3CA, PIK3R1	53	115	6879	5,64	0,813	0,0251	12,2

**THWAY**			85	083								446
**KEGG_PATHWAY**	hsa00562:Inositol phosphate metabolism	4	5,48	0,016623	PIK3CB, PIK3CD, PIK3CA, PTEN	53	71	6879	7,31	0,920	0,0369	17,8224
**KEGG_PATHWAY**	hsa04024:cAMP signaling pathway	6	8,22	0,016632	PIK3CB, PIK3CD, RAF1, PIK3CA, PTCH1, PIK3R1	53	198	6879	3,93	0,921	0,0366	17,9131
**KEGG_PATHWAY**	hsa04611:Platelet activation	5	6,85	0,016638	PLA2G4A, PIK3CB, PIK3CD, PIK3CA, PIK3R1	53	130	6879	4,99	0,921	0,0362	17,9745
**KEGG_PATHWAY**	hsa05162:Measles	5	6,85	0,017767	CCND1, PIK3CB, PIK3CD, PIK3CA, PIK3R1	53	133	6879	4,88	0,936	0,0385	19,2559
**KEGG_PATHWAY**	hsa05100:Bacterial invasion of epithelial cells	4	5,48	0,020882	PIK3CB, PIK3CD, PIK3CA, PIK3R1	53	78	6879	6,66	0,961	0,0446	22,3077
**KEGG_PATHWAY**	hsa04630:Jak-STAT signaling pathway	5	6,85	0,023446	CCND1, PIK3CB, PIK3CD, PIK3CA, PIK3R1	53	145	6879	4,48	0,974	0,0495	24,7836

H. Signaling pathways in which the potential targets of miR-369-5p are involved (identified by DAVID)

Table 1: targets identified by BIOCARTA (DAVID tool)

Table 2: targets identified by KEGG (DAVID tool)

Category	Term	Count	%	PValue	Genes	List Total	Pop Hits	Pop Total	Fold Enrichment	Bonferroni	Benjamini	FDR
**BIOCARTA**	h_g2Pathway:Cell Cycle: G2/M Checkpoint	3	30	0,0033	TP53, MDM2, ATR	7	25	1625	27,86	0,079	0,0789	2,6330
**BIOCARTA**	h_plk3Pathway:Regulation of cell cycle progression by Plk3	2	20	0,0292	TP53, ATR	7	8	1625	58,04	0,524	0,3098	21,3856
**BIOCARTA**	h_p53Pathway:p53 Signaling Pathway	2	20	0,0612	TP53, MDM2	7	17	1625	27,31	0,794	0,4094	40,1146
**BIOCARTA**	h_telPathway:Telomeres, Telomerase, Cellular Aging, and Immortality	2	20	0,0647	KRAS, TP53	7	18	1625	25,79	0,812	0,3419	41,9036
**BIOCARTA**	h_arfPathway:Tumor Suppressor Arf Inhibits Ribosomal Biogenesis	2	20	0,0647	TP53, MDM2	7	18	1625	25,79	0,812	0,3419	41,9036
**BIOCARTA**	h_atmPathway:ATM Signaling Pathway	2	20	0,0752	TP53, MDM2	7	21	1625	22,11	0,858	0,3235	46,9624
**BIOCARTA**	h_atrbrcaPathway:Role of BRCA1, BRCA2 and ATR in Cancer Susceptibility	2	20	0,0786	TP53, ATR	7	22	1625	21,10	0,871	0,2892	48,5508
**BIOCARTA**	h_ctcfPathway:CTCF: First Multivalent Nuclear Factor	2	20	0,0890	TP53, MDM2	7	25	1625	18,57	0,903	0,2830	53,0415
**BIOCARTA**	h_p53hypoxiaPathway:Hypoxia and p53 in the Cardiovascular system	2	20	0,0924	TP53, MDM2	7	26	1625	17,86	0,911	0,2613	54,4513

Category	Term	Count	%	PValue	Genes	List Total	Pop Hits	Pop Total	Fold Enrichment	Bonferroni	Benjamini	FDR
**KEGG_PATHWAY**	hsa05206:MicroRNAs in cancer	6	60	2,35E-06	KRAS, CD44, EZH2, TP53, MDM2, STMN1	8	286	6879	18,04	1,74E-04	1,74E-04	0,0025
**KEGG_PATHWAY**	hsa05219:Bladder cancer	3	30	7,14E-04	KRAS, TP53, MDM2	8	41	6879	62,92	0,0515	0,0261	0,7451
**KEGG_PATHWAY**	hsa05205:Proteoglycans in cancer	4	40	7,77E-04	KRAS, CD44, TP53, MDM2	8	200	6879	17,20	0,0559	0,0190	0,8106
**KEGG_PATHWAY**	hsa05214:Glioma	3	30	0,0018	KRAS, TP53, MDM2	8	65	6879	39,69	0,1242	0,0326	1,8585
**KEGG_PATHWAY**	hsa04115:p53 signaling pathway	3	30	0,0019	TP53, MDM2, ATR	8	67	6879	38,50	0,1314	0,0278	1,9726
**KEGG_PATHWAY**	hsa05218:Melanoma	3	30	0,0021	KRAS, TP53, MDM2	8	71	6879	36,33	0,1462	0,0260	2,2104
**KEGG_PATHWAY**	hsa05220:Chronic myeloid leukemia	3	30	0,0022	KRAS, TP53, MDM2	8	72	6879	35,83	0,1500	0,0229	2,2718
**KEGG_PATHWAY**	hsa05215:Prostate cancer	3	30	0,0033	KRAS, TP53, MDM2	8	88	6879	29,31	0,2146	0,0297	3,3590
**KEGG_PATHWAY**	hsa04919:Thyroid hormone signaling pathway	3	30	0,0055	KRAS, TP53, MDM2	8	115	6879	22,43	0,3355	0,0444	5,6169
**KEGG_PATHWAY**	hsa05169:Epstein-Barr virus infection	3	30	0,0062	CD44, TP53, MDM2	8	122	6879	21,14	0,3680	0,0448	6,2832

**KEGG_PATHWAY**	hsa04110:Cell cycle	3	30	0,0064	TP53, MDM2, ATR	8	124	6879	20,80	0,3772	0,0421	6,4793
**KEGG_PATHWAY**	hsa05203:Viral carcinogenesis	3	30	0,0168	KRAS, TP53, MDM2	8	205	6879	12,58	0,7149	0,0993	16,2624
**KEGG_PATHWAY**	hsa04010:MAPK signaling pathway	3	30	0,0250	KRAS, TP53, STMN1	8	253	6879	10,20	0,8469	0,1344	23,3098
**KEGG_PATHWAY**	hsa05166:HTLV-I infection	3	30	0,0252	KRAS, TP53, ATR	8	254	6879	10,16	0,8490	0,1263	23,4630
**KEGG_PATHWAY**	hsa05216:Thyroid cancer	2	20	0,0292	KRAS, TP53	8	29	6879	59,30	0,8880	0,1358	26,6305
**KEGG_PATHWAY**	hsa04151:PI3K-Akt signaling pathway	3	30	0,0445	KRAS, TP53, MDM2	8	345	6879	7,48	0,9657	0,1900	37,9328
**KEGG_PATHWAY**	hsa05213:Endometrial cancer	2	20	0,0518	KRAS, TP53	8	52	6879	33,07	0,9804	0,2065	42,6610
**KEGG_PATHWAY**	hsa05223:Non-small cell lung cancer	2	20	0,0556	KRAS, TP53	8	56	6879	30,71	0,9855	0,2097	45,0721
**KEGG_PATHWAY**	hsa05200:Pathways in cancer	3	30	0,0565	KRAS, TP53, MDM2	8	393	6879	6,56	0,9865	0,2026	45,5790
**KEGG_PATHWAY**	hsa05210:Colorectal cancer	2	20	0,0614	KRAS, TP53	8	62	6879	27,74	0,9908	0,2091	48,5023
**KEGG_PATHWAY**	hsa05230:Central carbon metabolism in cancer	2	20	0,0634	KRAS, TP53	8	64	6879	26,87	0,9921	0,2060	49,5981
**KEGG_PATHWAY**	hsa05212:Pancreatic cancer	2	20	0,0643	KRAS, TP53	8	65	6879	26,46	0,9927	0,2004	50,1374
